# Transcriptomics-Based Evaluation of the Effects of Polyethylene Microplastics on *Pleurotus pulmonarius*

**DOI:** 10.3390/foods14213783

**Published:** 2025-11-04

**Authors:** Xin Yu, Bo Zhang, Shuyi Chen, Caijing Wan, Sumin Chen, Ying Wang, Lei Ye, Xiaolin Li

**Affiliations:** 1 Sichuan Institute of Edible Fungi, Sichuan Academy of Agricultural Sciences, Chengdu 610066, China; 2 Key Laboratory of Coarse Cereal Processing, Ministry of Agriculture and Rural Affairs, Sichuan Engineering & Technology Research Center of Coarse Cereal Industrialization, School of Food and Biological Engineering, Chengdu University, Chengdu 610106, China

**Keywords:** *Pleurotus pulmonarius*, microplastic, transcriptome, agronomic traits

## Abstract

Microplastics are widely distributed, but their potential impact on crops cannot be ig-nored. Most current studies focus on common crops such as rice and buckwheat and are mostly at the macro level. In this study, we explored for the first time the changes in agro-nomic traits of *Pleurotus pulmonarius* by PE-MPs with different concentrations and particle sizes and applied confocal scanning microscopy (CLSM) to observe the uptake of PE-MPs by *P. pulmonarius* hyphae and combined it with transcriptomics to reveal the stress mech-anism of PE-MPs at the molecular level. Results indicate that among the small-particle groups, only the A5 and A20 groups exhibited significantly lower fresh weight than the CK group. The A5 group was 33.83% lower than the control, while the A20 group was 63.21% lower than CK (*p* < 0.05). Both the A5 and A20 groups showed significantly lower dry weight than the CK group (*p* < 0.05). Cap thickness was only greater in the B5 and B10 groups, exceeding the control by 1.46 mm and 1.58 mm, respectively. Cap length was longer only in the A10 group, increasing by 7.85% compared to the control (*p* < 0.05). Cap width in the A5 and A20 groups was 25.44% and 6.65% lower than the control, respec-tively (*p* < 0.05). Transcriptomics showed that as the concentration of PE-MPs increased, *P. pulmonarius* responded to PE-MPs stress by up-regulating the expression of cell membrane composition and metal–ion binding-related genes, while as the particle size increased, *P. pulmonarius* resisted the toxic effects by up-regulating the coming carbon metabolism and amino acid metabolism. Compared with the CK group, 1706, 1378, and 792 DEGs were identified in the A5, B5, and B10 groups, respectively. A total of 1610 DEGs were identified between the A5 and B5 groups. Additionally, 295 DEGs were identified between the A5 and B10 groups, while 1424 DEGs were identified between the B5 and B10 groups. This study reveals the effects of PE-MPs on the agronomic traits of *P. pulmonarius* and their re-sponse mechanisms, further indicating their potential risk to edible fungi.

## 1. Introduction

Plastic materials are extensively utilized in our daily lives due to their versatility, lightweight, affordability, and resistance to corrosion [1]. Weathered plastic waste [2], light [3], and radiation [4] can decompose into microplastics with particle sizes < 5 mm. After microplastics (MPs) enter the soil, they have the ability to alter the physical, chemical, and biological properties of the soil, which can have a direct or indirect impact on plant growth and soil health [5]. In addition, plants can also affect their growth through direct contact with MPs. Qi et al. [6] found that plants exposed to MPs experienced delayed germination, as well as affected growth and nutrition. Bosker et al. [7] showed that MPs could accumulate in the stomata of the testa of *Lepidium sativum* during germination. As a small part of a plant, crops are eaten directly by humans [8]; therefore, harm is caused when MPs eventually make their way into the human body through enrichment in the food chain. Research has shown that microplastics can be detected in human feces [9], breast milk [10], blood [11], and urine [12] while growing edible fungi. The routes of exposure to microplastics may include microplastics in atmospheric precipitation, particles in the water pipe wall during the water addition process, and friction between fungus bags. This can result in the inclusion of residues of aged plastic particles. There are few studies on how microplastics enter crops. Therefore, it is necessary to study whether crops can absorb microplastics.

Polyethylene holds the title of being the plastic with the highest production volume globally. Its main uses include making films, packaging materials, wires, and cables. It is a thermoplastic resin material obtained by the polymerization and processing of ethylene as a raw material. However, with its high molecular weight, powerful hydrophobicity, and resistant properties, it has become a major danger to the environment [13,14,15,16]. Current reports have mainly focused on the effects of polyethylene on rice (*Oryza sativa* L.) [17], wheat (*Triticum aestivum* L.) [6], Chinese cabbage (*Brassica rapa var. glabra Regel.*) [18], red amaranth (*Amaranthus tricolor* L.) [19], and soybean (*Glycine max* (L.) *Merr.*) [20]. Toxic effects on crop physicochemical parameters and cytogenetics [21] have also been studied. Current studies have focused mainly on common crops, and the effects and toxicity of polyethylene on the growth of edible fungi have not been reported. It has been established that plants primarily absorb microplastics through their roots, with vascular tissues transporting these particles [22]. Research on macrofungi remains unexplored, and the specific alterations macrofungi undergo under microplastic stress remain unclear. Furthermore, studies have detected microplastics in zooplankton and plants, demonstrating their potential to accumulate through the food chain and enter the human body [23]. Consequently, investigating edible fungi presents a significant avenue for further research.

*P. pulmonarius* is one of the most common edible fungi. In addition to its high nutritional value, it is also a natural source of prebiotics [24] and antioxidants [25]. In a medical study, *P. pulmonarius* exhibited anti-inflammatory effects [26] and analgesic and antitumor activities [27]. The growth of *P. pulmonarius* mainly depends on the hyphae to absorb nutrients from the microbial bag. Therefore, it is more likely to absorb microplastics in the microbial bag. At present, studies of *P. pulmonarius* have focused mainly on nutrition and cultivation; no one has studied the effects of PE-MPs on its growth.

In this study, the effects of the degradation of PE-MPs by *P. pulmonarius* on the agronomic traits of the fruiting bodies of *P. pulmonarius* were investigated. In this study, we explored for the first time the changes in agronomic traits of *P. pulmonarius* by different concentrations and particle sizes of PE-MPs and applied confocal scanning microscopy (CLSM) to observe the uptake of PE-MPs by *P. pulmonarius* hyphae and to reveal the stress mechanism of PE-MPs at the molecular level in combination with transcriptomics. This study, for the first time, demonstrated the effects of PE-MPs on the agronomic traits of *P. pulmonarius* and the response mechanism, thereby suggesting their potential risk to edible fungi. Theoretical insights provided in this study are essential for a holistic understanding of the toxicity of PE-MPs towards *P. pulmonarius* and are instrumental in driving the progress of edible fungi.

## 2. Materials and Methodology

### 2.1. Experimental Materials

The PE-MPs utilized in this research were bought from Guangyuan Plastic Chemical Co., Ltd. (Guangzhou, China). Two particle sizes were used for the PE-MPs: 100 μm and 500 μm. The melting point ranged from 100 to 115 °C, with a density of 0.913 g/cm^3^. The MPs were disinfected with 75% ethanol and placed in a 55 °C oven overnight for future use [28]. The *P. pulmonarius* strain used in this study was obtained from the Institute of Edible Fungi, Sichuan Academy of Agricultural Sciences. Preliminary studies have shown that it can degrade HDPE, with a degradation rate of 5.99%.

Fluorescent PE microspheres that are monodisperse were bought from Zhongke Keyou Nanotechnology Co., Ltd. (Beijing, China). The monodisperse PE microspheres had a particle size of 500 ± 50 nm, a concentration of 10 mg/mL, a density of approximately 1.03 g/cm^3^, and a CV < 5%. Cyanine5 fluorescent dye was added to the monodisperse PE microspheres at an excitation wavelength of 632 nm and an emission wavelength of 680 nm. The samples were refrigerated at 2–8 °C in the dark. We diluted the concentration of PE microspheres to 125 g/mL.

### 2.2. Experimental Design

The raw materials of the mushroom sticks were provided by Xin Zhongyu Agriculture, Suining City, China. The experiment was conducted in Pengxi, Sichuan, from February to May 2024. The microbial sticks in this experiment had been inoculated in the previous stage and were all grown in the same environment at 25–27 °C, with a relative air humidity of 85–90%, and protected from light. The composition of the fungal inoculum package is as follows: corn cobs 70%, cottonseed hulls 15%, wheat bran 7%, soybean meal 7%, lime 0.5%, and gypsum 0.5%. Before the experiment, the pretreated microplastics were irradiated with a UV lamp to simulate natural conditions. When the hyphae in the microbial mixture grow to approximately 1/10 of the size of the microbial mixture, the microplastics are added to it. MPs were grouped according to particle size and concentration as follows: small particle size low concentration (100 μm, 5 g), small particle size medium concentration (100 μm, 10 g), small particle size high concentration (100 μm, 20 g), large particle size low concentration (500 μm, 5 g), large particle size medium concentration (500 μm, 10 g), and large particle size high concentration (500 μm, 20 g) and were named A5, A10, A20, B5, B10, and B20. No microplastics were added in the control group, named CK. All the packages were placed under the same conditions for growth. To avoid contamination during growth, 10 replicates were set for each treatment group. The above quantities represent the amount added per fungal packet.

### 2.3. Determination of the Absorption of PE-MPs by P. pulmonarius

A total of 50 mL of 125 g/mL PE fluorescent microspheres was added to the microbial packets with consistent growth status. The fungal sticks were grown at 24 °C and 80% air humidity in a ventilated and protected environment. Samples were collected after 10 days to explore whether they could be absorbed by mycelia. The blank control was without microspheres, and three replicates were set up in each group. Mycelium from *P. pulmonarius* was collected from different treatments at the same site and rinsed three times with sterile water. Then a small portion was taken with sterile forceps and placed on a slide. After adding 10% (*v*/*v*) glycerol dropwise, the coverslips were covered. The uptake of PE-MPs by *P. pulmonarius* hyphae was observed in 2D mode via CLSM (Olympus FV1000, Tokyo, Japan) [22].

### 2.4. Determination of Agronomic Traits

The mushrooms were picked according to the picking standards of commercial mushrooms. The mushrooms on each stick were used as samples, and agronomic traits such as fresh weight, dry weight, water content, cap thickness, cap length, cap width, stipe diameter, and stipe length were measured using an analytical balance and a Vernier caliper.

### 2.5. Sample Collection and RNA Extraction

With *P. pulmonarius* as the research object, based on differences in agronomic traits, the CK group, the 100 μm/5 g group (A5), the 500 μm/5 g group (B5), and the 500 μm/10 g group (B10) were selected as samples. The samples were placed in an incubator filled with ice packs for temporary storage and then transported to the laboratory for RNA extraction and sequencing. Fruiting bodies were processed to extract total RNA with the help of TRIzol^®^ reagent (Invitrogen, Carlsbad, CA, USA). The RNA concentration was subsequently determined via a spectrophotometer [29]. The integrity of the RNA was detected via an Agilent 2100 bioanalyzer (Agilent, Santa Clara, CA, USA) [30].

### 2.6. Library Preparation

mRNA was enriched from total RNA using Oligo dT beads, fragmented, and synthesized into cDNA [31]. Then, end repair, A-tail addition, adapter ligation, fragment selection, amplification, and purification were performed.

### 2.7. Sequencing, Functional Annotation, Identification, and Enrichment Analysis of DEGs

After library construction was completed, preliminary quantification was first performed using a Qubit 2.0 Fluorometer. The libraries were diluted to 1.5 ng/μL and then detected using the Agilent 2100 Bioanalyzer (Agilent, USA). For qualified libraries, the effective concentration was accurately quantified using qRT-PCR. Illumina sequencing was performed after pooling as required, and 150 bp double-ended read lengths were generated. Finally, an index of the reference genome was constructed using Hisat2 v2.0.5 and compared to the reference genome.

All genes were annotated against Gene Ontology (GO) and the Kyoto Encyclopedia of Genes and Genomes (KEGG) [32]. Clean reads using the RSEM method to complete the remapping and assembly of the transcriptome [33]. The expression level of each gene was calculated using the FPKM method [34]. Differential expression analysis using the DESeq2 R package (1.48.1) [35]. When |log2 (FoldChange)| ≥ 1 and padj ≤ 0.05, the cells were considered to be differentially expressed under two different conditions [36]. For the enrichment analysis of DEGs, the GOseq R package and the KOBAS Web server were used to perform GO enrichment analysis and KEGG enrichment analysis of DEGs, respectively.

### 2.8. Data Analysis

All samples included three replicates. Analysis of variance (ANOVA) was performed on the data using SPSS 21.0. The Tukey test was used to differentiate significantly different means. The means were then subjected to univariate analysis using the Duncan multiple range test to obtain the *p*-value. *p* < 0.05 was considered significant.

## 3. Results and Analysis

### 3.1. Uptake of PE Microspheres by Hyphae

**Figure 1:**
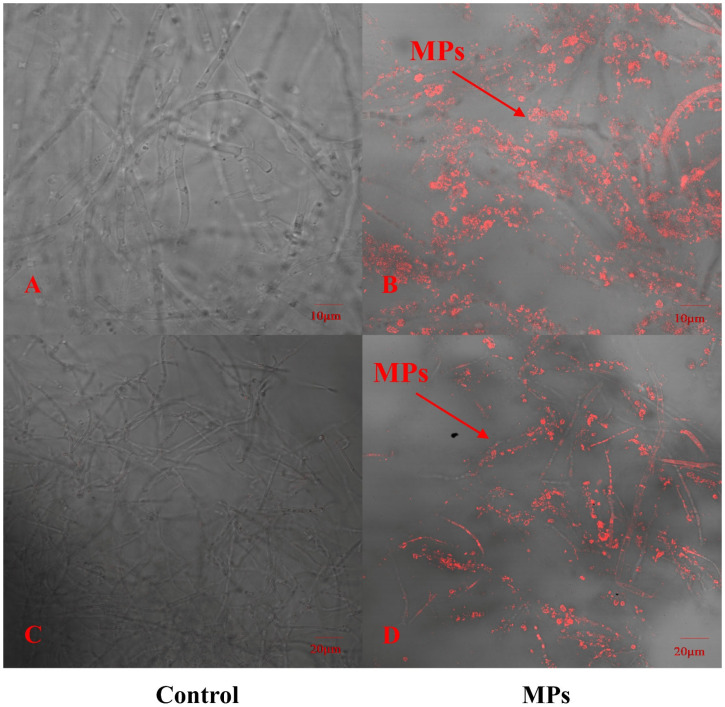
Distribution of fluorescent polyethylene microspheres on *P. pulmonarius* hyphae after 30 days of culture. (**A**) Controls were observed at a scale of 10 μm. (**B**) The experimental group was observed at a scale of 10 μm. (**C**) Controls were observed at a scale of 20 μm. (**D**) The experimental group was observed at a scale of 20 μm.

To explore whether PE-MPs can be absorbed by *P. pulmonarius*, this study used red fluorescently labeled PE microspheres combined with CLSM to detect the locations that were in contact with red fluorescent PE microspheres. In addition, we selected the same site where PE Micrococcus packs were not added to the sample as a control. As shown in Figure 1, the hyphae of *P. pulmonarius* in the blank control did not have any red fluorescent labeling, whereas many red fluorescent PE microspheres clearly appeared inside and on the surface of the *P. pulmonarius* hyphae exposed to the PE microspheres. With the help of CLSM, the images revealed that PE-MPs could enter the interior of the edible fungi through the hyphae to a certain extent.

### 3.2. Effects of Microplastics on Agronomic Traits

**Figure 2:**
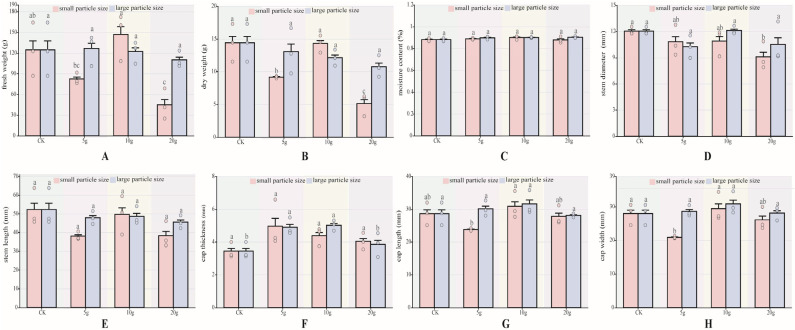
Agronomic traits of *P. pulmonarius* under stress of PE-MPs (*n* = 10). (**A**) Fresh weight. (**B**) Dry weight. (**C**) Moisture content. (**D**) Stem diameter. (**E**) Stem length. (**F**) Cap thickness. (**G**) Cap length. (**H**) Cap width. Different letters indicate significant differences between the samples (*p* < 0.05).

In this study, the fresh weight, dry weight, water content, cap thickness, cap length, cap width, stem diameter, and stem length of *P. pulmonarius* after maturity were measured. There are several differences in agronomic traits. For each agronomic trait index, data were analyzed according to particle size. The results showed that the moisture content (Figure 2C), stem length (Figure 2E) showed no significant differences between *P pulmonarius* treated with either large- or small-particle-size microplastics and the control group of both the large and small particle sizes of microplastic-added *P. pulmonarius* were not significantly different from the control. In terms of fresh weight (Figure 2A), no significant differences were observed among the large-particle-size groups (*p* < 0.05). Among the small-particle-size groups, only the A5 and A20 groups showed significantly lower fresh weights than the CK group (*p* < 0.05). Specifically, the A5 group recorded 82.54 mm, 33.83% lower than the control group, while the A20 group recorded the lowest value at 45.15 mm, 63.21% lower than the CK group (*p* < 0.05). For dry weight (Figure 2B), the A5 the A20 group was significantly lower than the CK group (*p* < 0.05).

Stem length showed no significant differences (Figure 2E) (*p* < 0.05). Cap thickness differed significantly only in the B5 and B10 groups, which were 1.46 mm and 1.58 mm thicker than the control group, respectively. Cap length (Figure 2G) was significantly longer only in the A10 group, at 7.85% longer than the control group (*p* < 0.05). Regarding cap width (Figure 2H), significant differences were observed only in the A5 and A20 groups (*p* < 0.05), with values 25.44% and 6.65% lower than the control group, respectively. 

### 3.3. Transcriptome Analysis of P. pulmonarius at Different Concentrations and Particle Sizes Under PE-MPs Stress

#### 3.3.1. Sequencing Data

**Figure 3:**
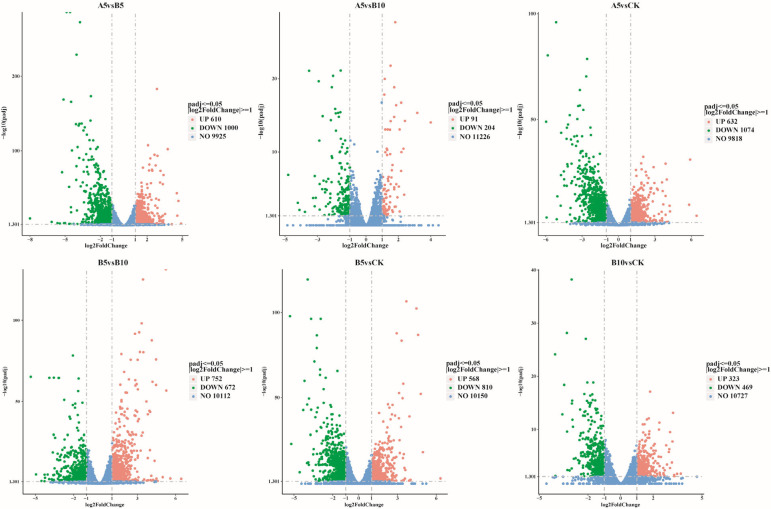
Comparative changes in DEGs. Differentially expressed genes were compared across different groups. Red indicates upregulated genes, green indicates downregulated genes, and blue indicates genes with no difference.

From the off-machine data of 12 samples, we obtained a total of 575,030,570 original reads. After data filtering, we finally obtained 545,562,676 clean reads (81.84G). The clean reads consisted of G and C bases that accounted for 53.86%, while bases with Phred values greater than 30 made up 97.02% on average (Table S1). PCA analysis showed significant differences between sample groups. PC1 and PC2 accounted for 50% and 50% of the sample variance, respectively (Figure 3).

#### 3.3.2. Analysis of Differentially Expressed Genes (DEGs)

**Figure 4:**
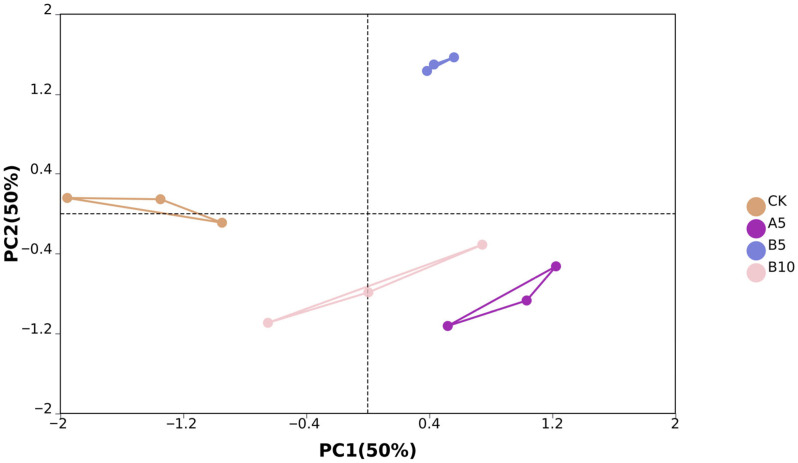
Sample principal component analysis. Principal component analysis was performed between the control group, A5 group, B5 group, and B10 group.

This study used DESeq2 software (1.48.1) to screen genes according to the criteria of |log2(FoldChange)| ≥ 1 and padj ≤ 0.05. Compared with the DEGs in the CK sample (Figure 4), 1706 DEGs were found in the A5, B5, and B10 samples (632 upregulated, 1074 downregulated), 1378 (568 upregulated, 810 downregulated), and 792 (323 upregulated, 469 downregulated) DEGs, respectively. In addition, 1610 DEGs (610 upregulated and 1000 downregulated) were found between the A5 and B5 samples. A total of 295 DEGs (91 upregulated and 204 downregulated) were found between the A5 and B10 samples, and 1424 DEGs (752 upregulated and 672 downregulated) were found between the B5 and B10 samples. When the particle size stayed the same and the concentration rose (Figure S1A), the amount of differentially expressed genes (DEGs) also rose. However, the number of upregulated DEGs tended to decrease, while the number of downregulated DEGs tended to increase. In addition, as the particle size increased (Figure S1B), the number of upregulated DEGs and downregulated DEGs tended to decrease.

#### 3.3.3. Cluster Analysis

We used the hierarchical clustering method to analyze the FPKM values of genes and performed normalization (Z score). The gene expression levels of the experimental group and the control group were not the same (Figure S2). The CK, B5, and B10 groups presented significant differences in the concentrations of PE-MPs.

**Figure 5:**
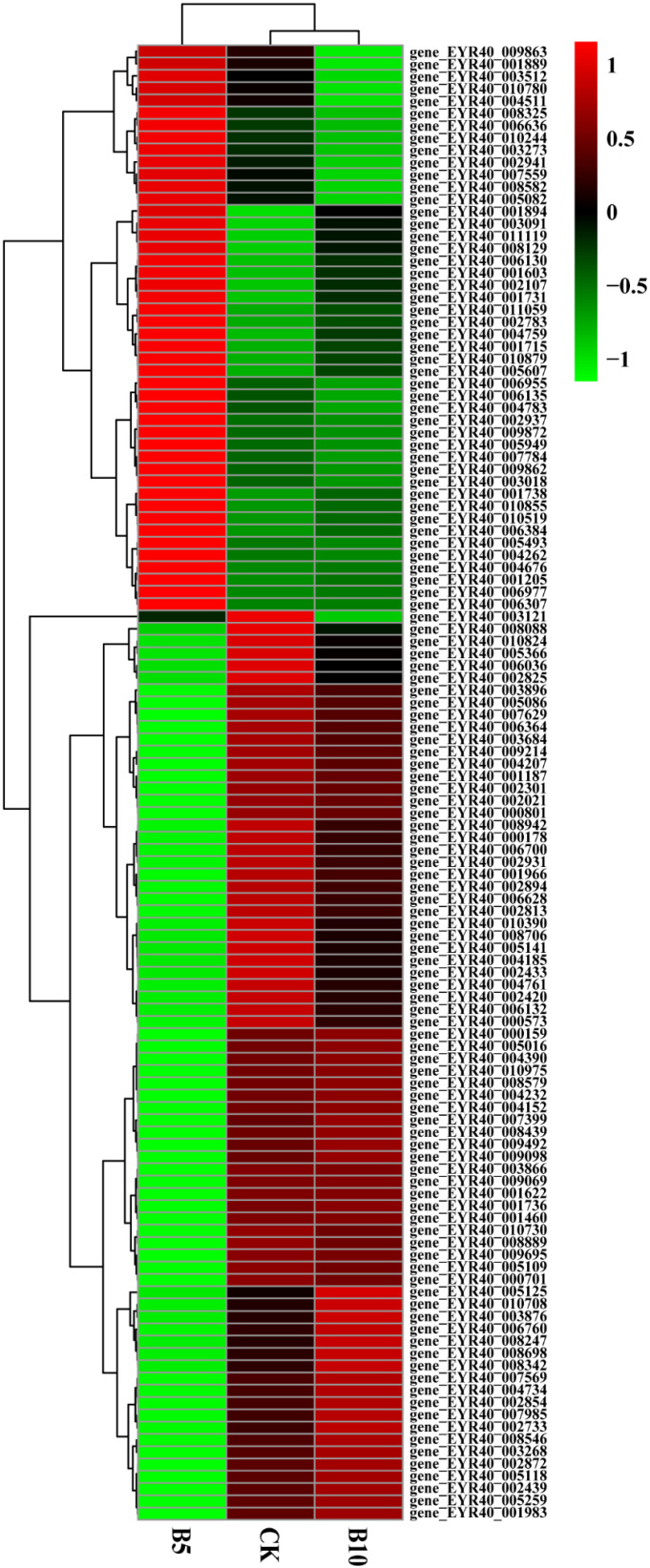
Heatmap of genes associated with growth. Cluster analysis was performed on the control group, B5 group, and B10 group. Red indicates gene upregulation, while green indicates gene downregulation.

In addition, we screened *P. pulmonarius* riae treated with microplastics of the same particle size and different concentrations. The levels of 74 genes initially decreased but later increased as the microplastic concentration increased (Figure 5). These genes play functions in different aspects of plant growth and development, including energy metabolism, antioxidant defense, amino acid synthesis, osmotic pressure regulation, signal transduction, lipid metabolism, and water management. Normal plant growth and adaptation to stress are critical. There were also 46 genes whose expression levels first increased but then decreased as the microplastic concentration increased. They are involved in cell wall synthesis, metabolic pathways, redox reactions, and signal transduction. They directly or indirectly affect plant growth and development.

#### 3.3.4. Functional Enrichment Analysis

We utilized the KEGG database to identify significantly enriched DEGs for pathway analysis in order to clarify the relationships between DEGs and metabolic pathways (Table S2). Function enrichment results are summarized in Table 1. With increasing particle size, DEGs related to biosynthesis of secondary metabolites, steroid biosynthesis, terpenoid backbone biosynthesis, pentose and glucuronate interconversions, butanoate metabolism, and tropane, piperidine, and pyridine alkaloid biosynthesis were significantly enriched (*p* < 0.05) (Figure S3A). As the concentration increased, DEGs related to the biosynthesis of secondary metabolites, terpenoid backbone biosynthesis, carbon metabolism, tryptophan metabolism, pyruvate metabolism, glyoxylate and dicarboxylate metabolism, and glycolysis/gluconeogenesis were significantly enriched (*p* < 0.05), and the expression of Bioscondylosis was highest (Figure S3B). A comparison of the CK group and the A5 group (Figure S3C) revealed that ribosome biosynthesis of secondary metabolites, the pentose phosphate pathway, DNA replication, steroid biosynthesis, glycolysis/gluconeogenesis, mismatch repair, terpenoid backbone biosynthesis, carbon metabolism, and carbonyl fixation in photosynthesis were significantly enriched (*p* < 0.05), with relatively high expression of ribosomes and biosynthesis of secondary metabolites. A comparison of the CK group and the B5 group (Figure S3D) revealed that biosynthesis of secondary metabolites, carbon metabolism, glycolysis/gluconeogenesis, the pentose phosphate pathway, biosynthesis of amino acids, cysteine and methionine metabolism, starch and sucrose metabolism, carbon fixation, biosynthesis of nucleotide sugars, pentose and glucuronate interconversions, fructose and mannose metabolism, glutathione metabolism, and biosynthesis of cofactor-related DEGs were significantly enriched (*p* < 0.05). Additionally, the expression of biosynthesis of secondary metabolites, carbon metabolism, and biosynthesis was relatively high. When comparing the CK group and the B10 group (Figure S3E), the differentially expressed genes (DEGs) related to the biosynthesis of secondary metabolites and glycolysis/gluconeogenesis were significantly enriched (*p* < 0.05). However, only the expression level of biosynthesis of secondary metabolites was relatively high.

In addition, we used the GO database to analyze the significantly enriched DEGs (Table S3). As the particle size increased (Figure S4A), carbohydrate metabolic processes, integral membrane components, intrinsic membrane components, extracellular regions, membrane parts, cofactor binding, iron ion binding, hydrolase activity, hydrolyzing O-glycosyl compounds, glycosidase activity, acting on glycosyl bonds, coenzyme binding, transition metal ion binding, oxidoreductase activity, acting on paired donors, incorporating or reducing molecular oxygen, heme binding, tetrapyrrole binding, and cation binding-related DEGs were significantly enriched (*p* < 0.05), and the expression levels of cofactor binding, cation binding, and metal ion binding were relatively high. With the same particle size, the concentration of PE-MPs changed (Figure S4B), and DEGs related to integral components of the membrane, intrinsic components of the membrane, coenzyme binding, cofactor binding, iron ion binding, oxidoreductase activity, heme binding, and tetrapyrrole binding were significantly enriched (*p* < 0.05), whereas the expression of cofactor binding, coenzyme binding, and integral components of the membrane was relatively greater. A comparison of the CK group and the A5 group (Figure S4C) revealed that the cellular amide metabolic process, translation, DNA replication, peptide biosynthetic process, amide biosynthetic process, peptide metabolic process, DNA-dependent DNA replication, DNA metabolic process, nonmembrane-bound organelles, intracellular nonmembrane-bound organelles, structural constituents of ribosomes, and structural molecule activities of the DEGs were significantly enriched (*p* < 0.05). The expression levels of the cellular amide metabolic process, nonmembrane-bound organelles, and intracellular nonmembrane-bound organelles were relatively high. When we compared the CK group and the B5 group (Figure S4D), we found that integral components of the membrane, intrinsic components of the membrane, membrane parts, cofactor binding, coenzyme binding, NADP binding, carbon-carbon lyase activity, oxidoreductase activity acting on the CH-OH group of donors, flavin adenine dinucleotide binding, oxidoreductase activity acting on the CH-OH group of donors with NAD or NADP as acceptors, and DEGs involved in FAD binding were significantly enriched (*p* < 0.05). Cofactor binding was expressed at the highest level. No significant enrichment of DEGs was observed in the CK group compared with the B10 group.

## 4. Discussion

### 4.1. Uptake of Microplastics by P. pulmonarius

This study revealed the enrichment of PE-MPs on the surface and inside of *P. pulmonarius* hyphae via confocal laser scanning microscopy (CLSM). Therefore, to a certain extent, PE-MPs can enter *P. pulmonarius* through hyphae. This discovery aligns with the results of past research. Submicrometer- and micrometer-sized particles of polystyrene and polymethylmethacrylate are able to penetrate the steles of Triticum aestivum and Lactuca sativa through crack entry mode at sites where lateral roots emerge [37]. Active cell division makes the plant’s apical meristem highly porous; therefore, microplastics can be captured by the root cap mucilage and enter the root. Because *P. pulmonarius* has no meristem, this study speculates that *P. pulmonarius* decomposes microplastics into small molecular substances through secreted enzymes and finally enters the interior of hyphae with the help of vectors such as transporters. This study investigated the uptake of PE microplastics by *P. pulmonarius*, but not their transport and distribution into the interior of *P. pulmonarius* or the main driving forces affecting the distribution of PE-MPs. Therefore, further research is needed to compensate for the deficiencies in the present study. According to the image findings, the PE fluorescent microsphere particle size ratio is smaller than the initial particle size. This may be due to degradation occurring during mushroom growth.

### 4.2. Effects of MPs on Agronomic Traits of P. pulmonarius

This study is the first to use different particle sizes and concentrations to stress *P. pulmonarius* growth. The results revealed significant differences only in four indicators: fresh weight, stem length, cap length, and cap width. Factors such as the dose of MPs, plant species, and stage of growth can influence their phytotoxicity [38]. Certain research has indicated that smaller particles have higher bioavailability, whereas increased concentrations of microplastics can impact plant growth. Yuan et al. [39] reported that the adsorption capacity of Pteris spores was negatively correlated with particle size. In other words, the smaller the NP dose, the easier the spores were adsorbed. The smaller particle size and higher concentration of *P. pulmonarius* may have caused obvious differences in agronomic traits. Wang et al. [40] reported that PE has no significant phytotoxic effect on corn (Z. mays), whereas under PS stress, even a low dose could have a negative effect on corn growth. Under 10% polylactic acid (PLA) stress, the biomass and chlorophyll content of corn leaves are significantly reduced [41]. PE did not cause phytotoxicity in corn, but it had a negative effect on the growth of *T. aestivum*, affecting the productivity aboveground and belowground [42]. This study only used PE-MPs and did not stress other types of MPs. We did not use the same material to stress the growth of other edible fungi, and the growth status of *P. pulmonarius* during different growth periods was not determined. Therefore, whether these factors are the result of the insignificant agronomic traits of *P. pulmonarius* remains to be further studied.

### 4.3. Cluster Analysis

In the present study, differentially expressed genes (DEGs) of *P. pulmonarius* grown under the stress of varying concentrations of PE-MPs were analyzed. The main significant changes were observed in genes involved in transporter proteins, detoxification and metabolism regulation, antioxidant and cell protection functions, as well as genes involved in metabolism, β-oxidation, and signal transduction of fatty acids. This fits with existing research to some extent. The upregulation of antioxidants is the first line of self-defense in plants [43]. Although only some indicators presented significant differences in agronomic traits, PE-MPs had a toxic effect on the growth of *P. pulmonarius*. Zhang et al. [44] found that exposure to PE triggered 31% of the plant antioxidant response. *P. pulmonarius* grows in an environment adapted to the stress of PE-MPs and thus may rely on multiple metabolic pathways to consume large amounts of energy. With increasing concentrations of PE-MPs from 5 to 10 g, the expression levels of stress response-related DEGs, such as cytochrome P450 monooxygenase and Baeyer–Villiger monooxygenase, tended to decrease. This suggests that *P. pulmonarius* may be able to tolerate the toxicity of PE-MPs through a stress response during increasing concentrations of PE-MPs. As the iron concentration increased from 5 g to 10 g, the level of high-affinity iron permease tended to increase. These findings indicate that the ion balance in *P. pulmonarius* is not disrupted under PE-MPs stress [45]. These findings also indicated that, at this concentration, *P. pulmonarius* could also maintain normal growth through self-regulation.

### 4.4. KEGG Functional Enrichment Analysis

In the KEGG functional enrichment analysis, the functional annotations related to biosynthesis of secondary metabolites and terpenoid backbone biosynthesis were significantly different, regardless of particle size or concentration. Secondary metabolites such as terpenoids are involved in the protective mechanism against PE-MP stress [46]. This finding was similar to the DEG results, indicating that PE-MP stress in *P. pulmonarius* stimulated stress defense [47]. It can maintain normal growth through self-regulation.

Under the stress of PE-MPs with different particle sizes, we observed an interesting phenomenon. Acetyl-CoA is an important enzyme involved in butanoate metabolism and is a product of pentose and glucuronate interconversions. According to the pathway diagram of butanoate metabolism, this metabolic pathway was found to be involved in the TCA cycle, amino acid metabolism, and fatty acid degradation pathways. Butyrate can be converted to pyruvate through glycolysis. In addition, D-glucose in the pentose and gluconate interconversion pathway can also be converted to D-glucose-6-phosphate via the action of the enzyme 1.2.1.12 (hexokinase). This enters the glycolytic pathway for the production of acetyl-CoA, and acetyl-CoA is the starting substance of the terpenoid backbone biosynthesis pathway. In addition, the presence of PE-MPs affected the metabolized levels of tryptophan and L-glutamic acid. This effect indicates a major perturbation in a key metabolic pathway [48]. Muhammad Asad Ullah Asad et al. [49] reported that L-glutamate is closely related to plant resistance mechanisms and participates in the synthesis of stress response proteins. This phenomenon also enhances the tolerance of *P. pulmonarius* to unfavorable conditions. Under PE-MP stress, *P. pulmonarius* further enhances the amino acid metabolism system to produce essential amino acids and other protective substances. Additionally, amino acid metabolism participates in the tricarboxylic acid cycle, which may provide energy for cell growth. As the concentration increased, pyruvate metabolism, glycolysis, etc., were significantly enriched. Wang et al. [50] stated that energy and carbon sources for nitrogen metabolism are provided by carbon metabolism. Thus, it is explained that to adapt to higher concentrations of stress, more energy may need to be provided.

### 4.5. GO Functional Enrichment Analysis

In the GO enrichment analysis, the enrichment function of the samples as a function of the PE-MPs concentration was mainly related to the CC and MF terms. The enriched cellular components were mainly related to the cell membrane. These results indicate that PE-MPs stress may affect the structure and components of the *P. pulmonarius* cell membrane and that the cell membrane also plays a key role in defending against external damage [51]. In terms of molecular function, it is mainly related to the reactions of coenzymes and metal ion binding. These findings indicate that a certain concentration of PE-MPs stimulated *P. pulmonarius* to exhibit cellular stress, relevant responses of secondary metabolic processes, and the potential influence of specific molecular function GO terms [52]. The GO-enriched functions mentioned above were also present in the *P. pulmonarius* functions that varied with particle size. However, there were also some functional enrichments related to metal ion binding, hydrolases, and carbohydrate metabolism. These results indicate that PE-MPs of different sizes may affect the transport of *P. pulmonarius* ions and require a large amount of capacity to maintain homeostasis.

Additionally, this study found that edible fungi can absorb microplastics, which have previously been detected in crops such as *Oryza sativa* L. and *Triticum aestivum* L. This could potentially accumulate through the food chain, ultimately entering the human body and affecting human health. However, for *P. pulmonarius*, it has been discovered that it can degrade microplastics, producing carbon dioxide and water. This capability outperforms other crops and simultaneously addresses environmental pollution issues. In future efforts to manage microplastic pollution, edible fungi could potentially be utilized as engineered strains. This discovery broadens the scope of edible fungi applications to a certain extent.

## 5. Conclusions

In this study, we investigated for the first time the effects of polyethylene microparticles (PE-MPs) of different concentrations and particle sizes on the agronomic traits of *P. pulmonarius*. We used confocal laser scanning microscopy (CLSM) to observe the absorption of PE-MPs by *P. pulmonarius* mycelium and combined transcriptomics technology to reveal the stress mechanisms of PE-MPs at the molecular level. Results indicate that among the small-particle groups, only the A5 and A20 groups exhib-ited significantly lower fresh weight than the CK group. The A5 group was 33.83% lower than the control, while the A20 group was 63.21% lower than CK (*p* < 0.05). Both the A5 and A20 groups showed significantly lower dry weight than the CK group (*p* < 0.05). Cap thickness was only greater in the B5 and B10 groups, exceeding the control by 1.46 mm and 1.58 mm, respectively. Cap length was longer only in the A10 group, increasing by 7.85% compared to the control (*p* < 0.05). Cap width in the A5 and A20 groups was 25.44% and 6.65% lower than the control, respectively (*p* < 0.05). Confocal laser scanning microscopy (CLSM) revealed that PE-MPs were significantly enriched on the surface and within the mycelium of *P. pulmonarius*. Additionally, transcriptomic analysis revealed that *P. pulmonarius* responds to PE-MP stress through gene regulation, including the expression of transport proteins, detoxification, and metabolic regulation, as well as antioxidant and cell protective functions. KEGG enrichment analysis results indicated that secondary metabolites (such as terpenoids) are involved in the protective mechanisms against PE-MPs stress. GO enrichment analysis indicated that *P. pulmonarius* maintains homeostasis through changes in cell membrane composition and glucose metabolism. This study first elucidates the effects of PE-MPs on the agronomic traits of *P. pulmonarius* and their response mechanisms, providing a theoretical basis for further understanding their toxicity to edible fungi.

## Figures and Tables

**Table 1:** KEGG and GO enrichment summary table. This includes A5 vs. B5, B5 vs. B10, CK vs. A5, CK vs. B5, CK vs. B10.

Group	KEGG Enrichment	GO Enrichment
A5 vs. B5	Biosynthesis of secondary metabolites	cation binding
	Steroid biosynthesis	tetrapyrrole binding
	Terpenoid backbone biosynthesis	heme binding
	Pentose and glucuronate interconversions	oxidoreductase activity, acting on paired donors, with incorporation or reduction in molecular oxygen
	Butanoate metabolism	transition metal ion binding
	Tropane, piperidine, and pyridine alkaloid biosynthesis	coenzyme binding
		coenzyme binding
		hydrolase activity, acting on glycosyl bonds hydrolase activity, hydrolyzing O-glycosyl compounds
		iron ion binding
		cofactor binding
		membrane part
		extracellular region
		intrinsic component of membrane
		carbohydrate metabolic process
		integral component of membrane
B5 vs. B10	Glycolysis/Gluconeogenesis	tetrapyrrole binding
	Glyoxylate and dicarboxylate metabolism	heme binding
	Pyruvate metabolism	oxidoreductase activity, acting on paired donors, with incorporation or reduction in molecular oxygen
	Tryptophan metabolism	iron ion binding
	Carbon metabolism	coenzyme binding
	Terpenoid backbone biosynthesis	cofactor binding
	Biosynthesis of secondary metabolites	intrinsic component of membrane
		integral component of membrane
CK vs. A5	Carbon fixation in photosynthetic organisms	structural molecule activity
	Carbon metabolism	structural constituent of ribosome
	Terpenoid backbone biosynthesis	intracellular non-membrane-bounded organelle
	Mismatch repair	non-membrane-bounded organelle
	Glycolysis/Gluconeogenesis	ribonucleoprotein complex
	Steroid biosynthesis	ribosome
	DNA replication	DNA metabolic process
	Pentose phosphate pathway	DNA-dependent DNA replication
	Biosynthesis of secondary metabolites	peptide metabolic process
	Ribosome	amide biosynthetic process
		peptide biosynthetic process
		DNA replication
		translation
		cellular amide metabolic process
CK vs. B5	Biosynthesis of cofactors	FAD binding
	Glutathione metabolism	oxidoreductase activity, acting on the CH-OH group of donors
	Fructose and mannose metabolism	flavin adenine dinucleotide binding
	Pentose and glucuronate interconversions	oxidoreductase activity, acting on the CH-OH group of donors, NAD or NADP as acceptor
	Biosynthesis of nucleotide sugars	carbon-carbon lyase activity
	Carbon fixation in photosynthetic organisms	NADP binding
	Starch and sucrose metabolism	coenzyme binding
	Cysteine and methionine metabolism	cofactor binding
	Biosynthesis of amino acids	membrane part
	Pentose phosphate pathway	intrinsic component of membrane
	Glycolysis/Gluconeogenesis	integral component of membrane
	Carbon metabolism	
	Biosynthesis of secondary metabolites	
CK vs. B10	Glycolysis/Gluconeogenesis	
	Biosynthesis of secondary metabolites	

## Data Availability

The original contributions presented in the study are included in the article/Supplementary Materials, further inquiries can be directed to the corresponding author.

## Supplementary Materials

The following supporting information can be downloaded at: https://www.mdpi.com/article/10.3390/foods14213783/s1, Figure S1: (A) Changes in co-expressed DEGs with particle size. (B) Changes in co-expressed DEGs as a function of concentration; Figure S2: Differential expression of DEGs as a function of concentration; Figure S3: Bubble diagram of KEGG enrichment; Figure S4: Bubble diagram of GO enrichment. Table S1: Alignment results of each sample; Table S2: List of significantly enriched results for the differential gene KEGG; Table S3: List of Significantly Enriched Results for Differential Gene GO; Table S4: Summary Table of Agronomic Parameters.
