# Supplementary material for: Transcriptomics-Based Evaluation of the Effects of Polyethylene Microplastics on Pleurotus pulmonarius
Source: Foods. 2025 Nov 4;14(21):3783. doi: 10.3390/foods14213783 (PMC12609591; doi:10.3390/foods14213783)
Supplement: Supplementary file 1 [file foods-14-03783-s001.zip › foods-3946607-supplementary.pdf]

Transcriptomics-Based Evaluation of the Effects of Polyethylene Microplastics on *Pleurotus pulmonarius*

Xin Yu [1,2], Bo Zhang [1], Shuyi Chen [2], Caijing Wan [2], Sumin Chen [2], Ying Wang [2], Lei Ye [1] and Xiaolin Li [1,*]

[1] Sichuan Institute of Edible Fungi, Sichuan Academy of Agricultural Sciences, Chengdu 610066, China; m18481822277@163.com (X.Y.); bozhang5658@foxmail.com (B.Z.); yeleidewangyi@163.com (L.Y.)

[2] Key Laboratory of Coarse Cereal Processing, Ministry of Agriculture and Rural Affairs, Sichuan Engineering & Technology Research Center of Coarse Cereal Industrialization, School of Food and Biological Engineering, Chengdu University, Chengdu 610106, China; 18882882771@163.com (S.C.); 13206009521@163.com (C.W.); chensumin76@163.com (S.C.); 19938308064@163.com (Y.W.)

* Correspondence: lixiaolin@scsaas.cn; Tel.: +86-15208358662

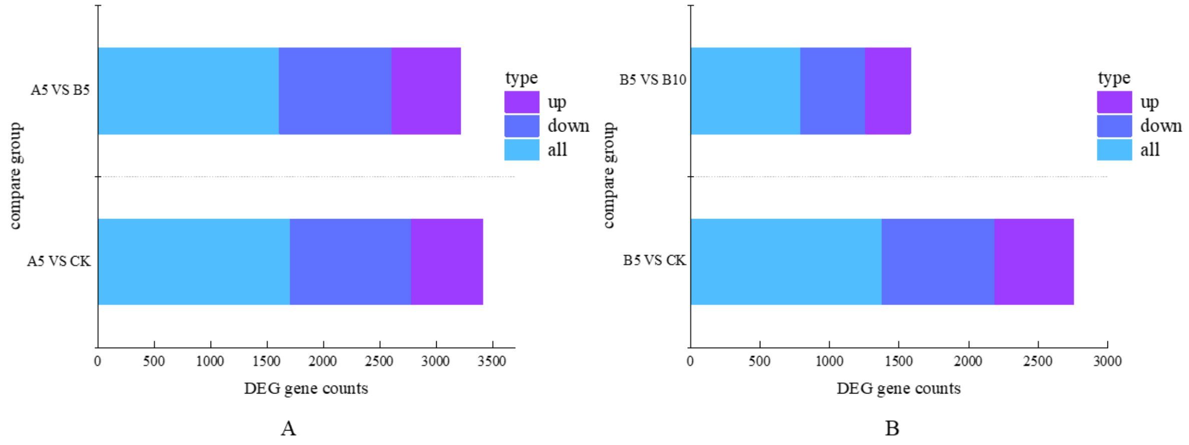

Figure S1.（A）Changes in co-expressed DEGs with particle size. (B) Changes in co-expressed DEGs as a function of concentration

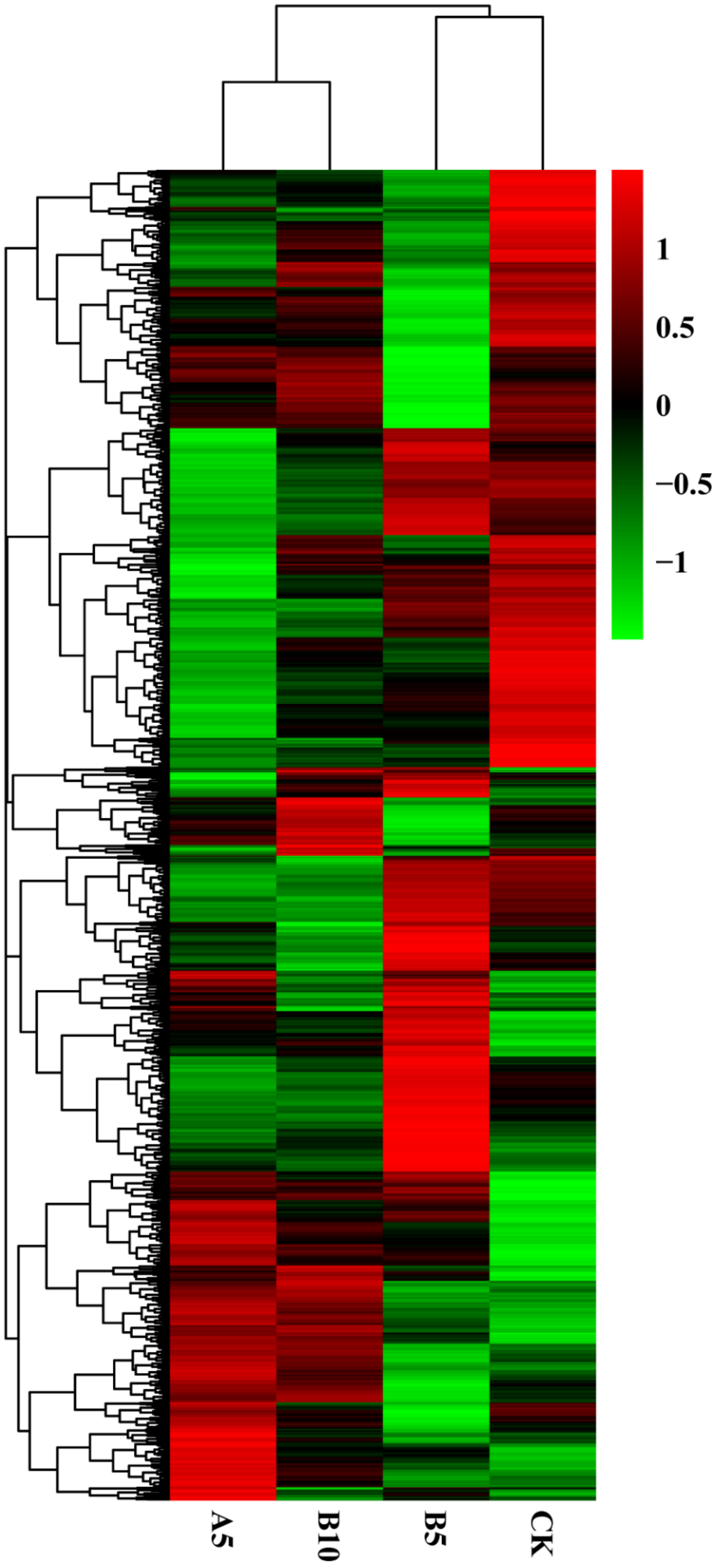

Figure S2. Differential expression of DEGs as a function of concentration

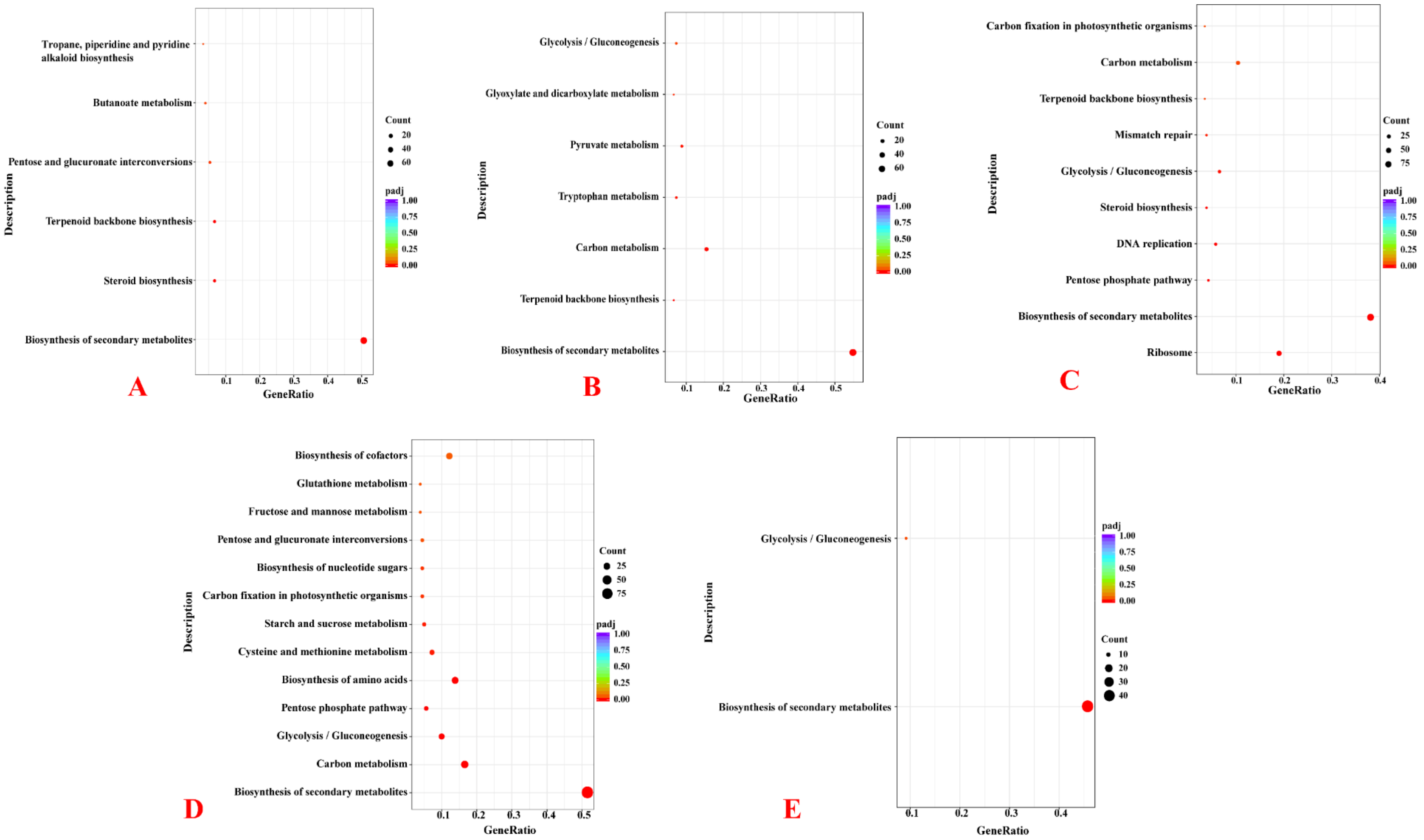

Figure S3. Bubble diagram of KEGG enrichment. (A) A5 vs B5. (B) B5 vs B10. (C) CK vs A5. (D) CK vs B5. (E) CK vs B10.

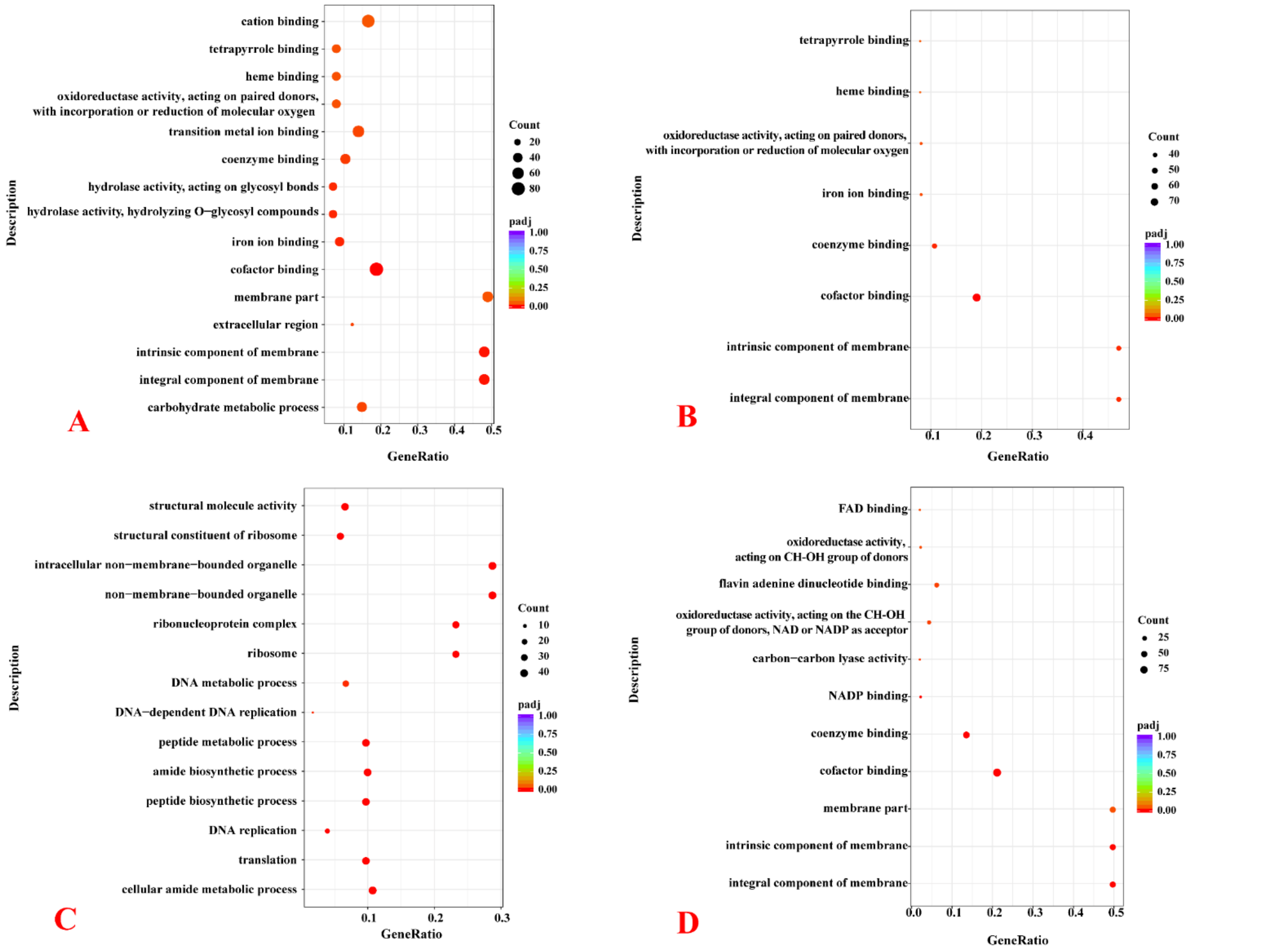

Figure S4. Bubble diagram of GO enrichment. (A) A5 vs B5. (B) B5 vs B10. (C) CK vs A5. (D) CK vs B5.

Table S1. Alignment results of each sample.

sample	raw_reads	raw_bases	clean_reads	clean_bases	error_rate	Q20	Q30	GC_pct
CK1	47491092	7.12G	45198466	6.78G	0.01	99.03	97.11	53.99
CK2	49930076	7.49G	46654468	7.0G	0.01	98.92	96.91	53.97
CK3	47592912	7.14G	45466948	6.82G	0.01	98.98	97.02	53.91
A5_1	49909060	7.49G	47527908	7.13G	0.01	99.01	97.04	53.61
A5_2	48344590	7.25G	46081478	6.91G	0.01	98.96	96.95	53.6
A5_3	47877146	7.18G	45291042	6.79G	0.01	99	96.98	53.62
B5_1	47652776	7.15G	45186418	6.78G	0.01	98.92	96.86	54.11
B5_2	47555148	7.13G	45143588	6.77G	0.01	98.98	97.01	54.15
B5_3	46184812	6.93G	44107184	6.62G	0.01	98.98	96.99	54.09
B10_1	48520134	7.28G	46179890	6.93G	0.01	98.93	96.92	53.72
B10_2	47004668	7.05G	44795970	6.72G	0.01	99.01	97.09	53.72
B10_3	46968156	7.05G	43929316	6.59G	0.01	99.15	97.34	53.77

CK, A5, B5, and B10 indicate that the concentrations of PE-MPs were 0g, 100 μm,5g, 500 μm,5g, and 500 μm,10g, respectively. Three biological replicates were designed for each treatment.

Table S2. List of significantly enriched results for the differential gene KEGG

KEGGID	Description	geneID	geneName	keggID	Count	Up	Up_Gene_id	Down	Down_Gene_id
**KEGG enrichment between A5 and B5 samples**									
ath01110	Biosynthesis of secondary metabolites	gene_EYR40_008586/gene_EYR40_008395/gene_EYR40_005369/gene_EYR40_004142/gene_EYR40_000111/gene_EYR40_006409/gene_EYR40_001974/gene_EYR40_010711/novel.610/gene_EYR40_003804/gene_EYR40_009845/gene_EYR40_004362/gene_EYR40_00	EYR40_008586/ERG1_1/ERG10/EYR40_004142/ACS1/MVD1/CHS2_1/ERG6/-/EYR40_003804/ERG7/ERG5/IDI1/PAN6/EYR40_006810/ERG26/ERG4/ERG24_1/HMG1/KGD1/EYR40_002735/SDH1_1/ERG11/SAM2/GUT2/ACL1/ZWF1/ERG25/BTS1/-/EYR40_009	ath:AT4G11820/ath:AT1G58440/ath:AT5G48230/ath:AT3G16910/ath:AT5G36880/ath:AT2G38700/ath:AT1G23800/ath:AT5G13710/ath:AT4G34640/ath:AT1G65060/ath:AT2G07050/ath:AT2G28860/ath:AT3G02780/ath:AT5G48840/ath:AT2G20340/ath:AT2G26260/ath:AT3	74	51	gene_EYR40_008586/gene_EYR40_008395/gene_EYR40_005369/gene_EYR40_004142/gene_EYR40_000111/gene_EYR40_006409/gene_EYR40_010711/novel.610/gene_EYR40_009845/gene_EYR40_004362/gene_EYR40_003985/gene_EYR40_008609/gene_EYR40_006	23	gene_EYR40_001974/gene_EYR40_003804/gene_EYR40_000333/gene_EYR40_007257/novel.193/gene_EYR40_009367/gene_EYR40_000693/gene_EYR40_002476/gene_EYR40_003086/gene_EYR40_002900/gene_EYR40_002845/gene_EYR40_004481/gene_EYR40_009

KEGGID	Description	geneID	geneName	keggID	Count	Up	Up_Gene_id	Down	Down_Gene_id
		3985/gene_EYR40_008609/gene_EYR40_006810/gene_EYR40_009498/gene_EYR40_008651/gene_EYR40_007981/gene_EYR40_002871/gene_EYR40_009773/gene_EYR40_002735/gene_EYR40_000333/gene_EYR40_008002/gene_EYR40_000905/gene_EYR40_004496/gene_EYR40_007898/ge	367/EYR40_002023/EYR40_002439/EYR40_004662/EYR40_003938/CYB5D1/INO1/EYR40_007985/EYR40_000693/EYR40_002476/TRP4/EYR40_002900/EYR40_010868/PYK1/ALD5_1/HIS5/MET6/EYR40_009595/EYR40_001983/EYR40_004481/EYR40_009275/EYR40_006900/EYR40_002707/-	G52940/ath:AT3G52940/ath:AT1G76490/ath:AT3G55410/ath:AT1G24360/ath:AT5G66760/ath:AT1G11680/ath:AT2G36880/ath:AT3G10370/ath:AT3G06650/ath:AT5G40760/ath:AT1G07420/ath:AT1G78510/ath:AT5G04330/ath:AT3G48560/ath:AT5G11720/ath:AT1G48130/ath:AT3G30775/ath:AT			810/gene_EYR40_009498/gene_EYR40_008651/gene_EYR40_007981/gene_EYR40_002871/gene_EYR40_009773/gene_EYR40_002735/gene_EYR40_008002/gene_EYR40_000905/gene_EYR40_004496/gene_EYR40_007898/gene_EYR40_005655/gene_EYR40_002425/gene_EYR40_002023/gen		275/gene_EYR40_006900/gene_EYR40_002707/novel.80/gene_EYR40_001458/gene_EYR40_003898/gene_EYR40_003449/gene_EYR40_008786/gene_EYR40_006394/gene_EYR40_009740/gene_EYR40_002669

KEGGID	Description	geneID	geneName	keggID	Count	Up	Up_Gene_id	Down	Down_Gene_id
		ne_EYR40_005655/gene_EYR40_002425/gene_EYR40_007257/novel.193/gene_EYR40_009367/gene_EYR40_002023/gene_EYR40_002439/gene_EYR40_004662/gene_EYR40_003938/gene_EYR40_004126/gene_EYR40_007279/gene_EYR40_007985/gene_EYR40_000693/gene_EYR40_002476	/PRO3/EYR40_001458/EYR40_003881/EYR40_007165/RPE1/EYR40_003449/EYR40_008786/EYR40_007106/EYR40_006647/EYR40_000113/EYR40_010880/EYR40_006394/EYR40_009740/EYR40_009629/EYR40_003963/EYR40_002931/EYR40_001793/ADH1_2/EYR40_001133/ERG20_1	1G17745/ath:AT3G14420/ath:AT2G22240/ath:AT2G42790/ath:AT1G15080/ath:AT4G35160/ath:AT5G17990/ath:AT1G65060/ath:AT1G05160/ath:AT5G63680/ath:AT1G23800/ath:AT1G71920/ath:AT5G17920/ath:AT1G20480/ath:AT5G07990/ath:AT5G06060/ath:AT1G24360/ath:AT3G53260/ath:			e_EYR40_002439/gene_EYR40_004662/gene_EYR40_003938/gene_EYR40_004126/gene_EYR40_007279/gene_EYR40_007985/gene_EYR40_010868/gene_EYR40_003896/gene_EYR40_001984/gene_EYR40_005259/gene_EYR40_009595/gene_EYR40_001983/gene_EYR40_000701/gene_EYR		

KEGGID	Description	geneID	geneName	keggID	Count	Up	Up_Gene_id	Down	Down_Gene_id
		/gene_EYR40_003086/gene_EYR40_002900/gene_EYR40_010868/gene_EYR40_003896/gene_EYR40_001984/gene_EYR40_002845/gene_EYR40_005259/gene_EYR40_009595/gene_EYR40_001983/gene_EYR40_004481/gene_EYR40_009275/gene_EYR40_006900/gene_EYR40_002707/novel.		AT3G03980/ath:AT4G31500/ath:AT5G14800/ath:AT1G74540/ath:AT5G06060/ath:AT3G61530/ath:AT1G63290/ath:AT4G13010/ath:AT5G08335/ath:AT1G74540/ath:AT5G08335/ath:AT3G12900/ath:AT2G37790/ath:AT1G31670/ath:AT5G08335/ath:AT4G25970/ath:AT2G29320/ath:AT5G17330/at			40_003881/gene_EYR40_007165/gene_EYR40_007106/gene_EYR40_006647/gene_EYR40_000113/gene_EYR40_010880/gene_EYR40_009629/gene_EYR40_003963/gene_EYR40_002931/gene_EYR40_001793/gene_EYR40_010824/gene_EYR40_001133		

KEGGID	Description	geneID	geneName	keggID	Count	Up	Up_Gene_id	Down	Down_Gene_id
		80/gene_EYR40_000701/gene_EYR40_001458/gene_EYR40_003881/gene_EYR40_007165/gene_EYR40_003898/gene_EYR40_003449/gene_EYR40_008786/gene_EYR40_007106/gene_EYR40_006647/gene_EYR40_000113/gene_EYR40_010880/gene_EYR40_006394/gene_EYR40_009740/gene		h:AT5G07990/ath:AT4G37980/ath:AT1G65960/ath:AT4G17190					

KEGGID	Description	geneID	geneName	keggID	Count	Up	Up_Gene_id	Down	Down_Gene_id
		_EYR40_009629/gene_EYR40_003963/gene_EYR40_002931/gene_EYR40_001793/gene_EYR40_010824/gene_EYR40_001133/gene_EYR40_002669							
ath00100	Steroid biosynthesis	gene_EYR40_008395/gene_EYR40_010711/novel.610/gene_EYR40_009845/gene_EYR40_004362/gene_EYR40_009498/gene_EYR40_008651	ERG1_1/ERG6/-/ERG7/ERG5/ERG26/ERG4/ERG24_1/ERG11/ERG25	ath:AT1G58440/ath:AT5G13710/ath:AT4G34640/ath:AT2G07050/ath:AT2G28860/ath:AT2G26260/ath:AT3G52940/ath:AT3G52940/ath:AT1G1	10	10	gene_EYR40_008395/gene_EYR40_010711/novel.610/gene_EYR40_009845/gene_EYR40_004362/gene_EYR40_009498/gene_EYR40_008651	0	

KEGGID	Description	geneID	geneName	keggID	Count	Up	Up_Gene_id	Down	Down_Gene_id
		/gene_EYR40_007981/gene_EYR40_008002/gene_EYR40_002425		1680/ath:AT1G07420			/gene_EYR40_007981/gene_EYR40_008002/gene_EYR40_002425		
ath00900	Terpenoid backbone biosynthesis	gene_EYR40_008586/gene_EYR40_005369/gene_EYR40_006409/gene_EYR40_003985/gene_EYR40_002871/gene_EYR40_007257/gene_EYR40_008786/gene_EYR40_006647/gene_EYR40_009740/gene_EYR40_002669	EYR40_008586/ERG10/MVD1/IDI1/HMG1/BTS1/EYR40_008786/EYR40_006647/EYR40_009740/ERG20_1	ath:AT4G11820/ath:AT5G48230/ath:AT2G38700/ath:AT3G02780/ath:AT1G76490/ath:AT1G78510/ath:AT5G08335/ath:AT5G08335/ath:AT5G08335/ath:AT4G17190	10	6	gene_EYR40_008586/gene_EYR40_005369/gene_EYR40_006409/gene_EYR40_003985/gene_EYR40_002871/gene_EYR40_006647	4	gene_EYR40_007257/gene_EYR40_008786/gene_EYR40_009740/gene_EYR40_002669

KEGGID	Description	geneID	geneName	keggID	Count	Up	Up_Gene_id	Down	Down_Gene_id
ath00040	Pentose and glucuronate interconversions	gene_EYR40_008676/gene_EYR40_000519/gene_EYR40_002894/gene_EYR40_003898/gene_EYR40_003253/gene_EYR40_006121/gene_EYR40_010880/gene_EYR40_010195	EYR40_008676/UGD1/EYR40_002894/RPE1/EYR40_003253/EYR40_006121/EYR40_010880/EYR40_010195	ath:AT5G51970/ath:AT1G26570/ath:AT5G51970/ath:AT1G63290/ath:AT5G27530/ath:AT4G22080/ath:AT2G37790/ath:AT4G35670	8	5	gene_EYR40_008676/gene_EYR40_000519/gene_EYR40_002894/gene_EYR40_003253/gene_EYR40_010880	3	gene_EYR40_003898/gene_EYR40_006121/gene_EYR40_010195
ath00650	Butanoate metabolism	gene_EYR40_008586/gene_EYR40_005369/gene_EYR40_004142/gene_EYR40_009367/gene_EYR40_002931/gene_E	EYR40_008586/ERG10/EYR40_004142/EYR40_009367/EYR40_002931/EYR40_001133	ath:AT4G11820/ath:AT5G48230/ath:AT3G16910/ath:AT3G48560/ath:AT5G17330/ath:AT1G65960	6	5	gene_EYR40_008586/gene_EYR40_005369/gene_EYR40_004142/gene_EYR40_002931/gene_EYR40_001133	1	gene_EYR40_009367

KEGGID	Description	geneID	geneName	keggID	Count	Up	Up_Gene_id	Down	Down_Gene_id
		YR40_001133							
ath00960	Tropane, piperidine and pyridine alkaloid biosynthesis	gene_EYR40_002845/gene_EYR40_004481/gene_EYR40_003881/gene_EYR40_006394/gene_EYR40_003963	HIS5/EYR40_004481/EYR40_003881/EYR40_006394/EYR40_003963	ath:AT1G71920/ath:AT5G06060/ath:AT5G06060/ath:AT1G31670/ath:AT2G29320	5	2	gene_EYR40_003881/gene_EYR40_003963	3	gene_EYR40_002845/gene_EYR40_004481/gene_EYR40_006394
**KEGG enrichment between B5 and B10 samples**									
ath01110	Biosynthesis of secondary metabolites	gene_EYR40_008586/gene_EYR40_004142/gene_EYR40_000111/gene_EYR40_002023/gene_EYR40_003392/gene_EYR40_008395/gene_EY	EYR40_008586/EYR40_004142/ACS1/EYR40_002023/EYR40_003392/ERG1_1/ERG6/EYR40_003804/HMG1/GUT2/ALD5_1/EYR40_009367/ZW	ath:AT4G11820/ath:AT3G16910/ath:AT5G36880/ath:AT5G11720/ath:AT2G42490/ath:AT1G58440/ath:AT5G13710/ath:AT1G65060/ath:AT1G7	74	17	gene_EYR40_003804/gene_EYR40_009367/gene_EYR40_001974/gene_EYR40_002900/gene_EYR40_010848/gene_EYR40_008361/gene_EY	57	gene_EYR40_008586/gene_EYR40_004142/gene_EYR40_000111/gene_EYR40_002023/gene_EYR40_003392/gene_EYR40_008395/gene_EY

KEGGID	Description	geneID	geneName	keggID	Count	Up	Up_Gene_id	Down	Down_Gene_id
		R40_010711/gene_EYR40_003804/gene_EYR40_002871/gene_EYR40_004496/gene_EYR40_001984/gene_EYR40_009367/gene_EYR40_005655/gene_EYR40_009498/gene_EYR40_006409/gene_EYR40_005369/gene_EYR40_001974/gene_EYR40_008709/gene_EYR40_004126/gene_EYR40_00	F1/ERG26/MVD1/ERG10/CHS2_1/CYS4/CYB5D1/EYR40_002900/EYR40_010848/ERG20_2/SAM2/EYR40_002439/EYR40_009595/EYR40_010868/EYR40_007985/GLK1/EYR40_010251/GPH1/EYR40_001793/EYR40_002476/EYR40_003938/EYR40_001856/POT1/PRO3/EYR40_006480/PYK1/MET	6490/ath:AT3G10370/ath:AT1G23800/ath:AT3G48560/ath:AT5G40760/ath:AT2G26260/ath:AT2G38700/ath:AT5G48230/ath:AT1G23800/ath:AT3G04940/ath:AT3G14420/ath:AT1G65060/ath:AT1G09400/ath:AT4G17190/ath:AT2G36880/ath:AT1G48130/ath:AT1G20480/ath:AT1G05160/ath:AT2			R40_002476/gene_EYR40_003086/gene_EYR40_006900/gene_EYR40_001458/gene_EYR40_007257/novel.80/gene_EYR40_002485/gene_EYR40_009740/gene_EYR40_002117/gene_EYR40_002707/gene_EYR40_002669		R40_010711/gene_EYR40_002871/gene_EYR40_004496/gene_EYR40_001984/gene_EYR40_005655/gene_EYR40_009498/gene_EYR40_006409/gene_EYR40_005369/gene_EYR40_008709/gene_EYR40_004126/gene_EYR40_000905/gene_EYR40_002439/gene_EYR40_009595/gene_EYR40_01

KEGGID	Description	geneID	geneName	keggID	Count	Up	Up_Gene_id	Down	Down_Gene_id
		2900/gene_EYR40_010848/gene_EYR40_008361/gene_EYR40_000905/gene_EYR40_002439/gene_EYR40_009595/gene_EYR40_010868/gene_EYR40_007985/gene_EYR40_005681/gene_EYR40_010251/gene_EYR40_010089/gene_EYR40_001793/gene_EYR40_002476/gene_EYR40_003938/ge	6/TRP4/EYR40_006900/EYR40_001458/EYR40_001983/EYR40_010880/BTS1/EYR40_003963/-/EYR40_002485/EYR40_000113/EYR40_002733/EYR40_006647/EYR40_003186/LEU2/EYR40_009740/EYR40_010085/ADE10/CAT1/PGI1/EYR40_002117/EYR40_002707/ADH1_2/6PGD/SOL1/EYR40_00	G42790/ath:AT2G19860/ath:AT4G16760/ath:AT3G46970/ath:AT5G07990/ath:AT4G35160/ath:AT1G17745/ath:AT1G20630/ath:AT2G33150/ath:AT5G14800/ath:AT4G05160/ath:AT5G63680/ath:AT5G17920/ath:AT5G17990/ath:AT3G53260/ath:AT1G74540/ath:AT5G07990/ath:AT2G37790/ath:AT					0868/gene_EYR40_007985/gene_EYR40_005681/gene_EYR40_010251/gene_EYR40_010089/gene_EYR40_001793/gene_EYR40_003938/gene_EYR40_001856/gene_EYR40_005944/gene_EYR40_000701/gene_EYR40_006480/gene_EYR40_003896/gene_EYR40_005259/gene_EYR40_001983/ge

KEGGID	Description	geneID	geneName	keggID	Count	Up	Up_Gene_id	Down	Down_Gene_id
		ne_EYR40_001856/gene_EYR40_005944/gene_EYR40_000701/gene_EYR40_006480/gene_EYR40_003896/gene_EYR40_005259/gene_EYR40_003086/gene_EYR40_006900/gene_EYR40_001458/gene_EYR40_001983/gene_EYR40_010880/gene_EYR40_007257/gene_EYR40_003963/novel.80/	0159/PYC1/EYR40_002931/ADE1/ERG20_1/EYR40_007106/EYR40_003302/EYR40_006626/EYR40_005016/EYR40_000112/ALTA7	1G78510/ath:AT2G29320/ath:AT4G31500/ath:AT1G73050/ath:AT3G12900/ath:AT3G57050/ath:AT5G08335/ath:AT3G03980/ath:AT1G31180/ath:AT5G08335/ath:AT2G06050/ath:AT2G35040/ath:AT1G20630/ath:AT5G42740/ath:AT3G03980/ath:AT3G03980/ath:AT4G37980/ath:AT5G41670/ath:					ne_EYR40_010880/gene_EYR40_003963/gene_EYR40_000113/gene_EYR40_002733/gene_EYR40_006647/gene_EYR40_003186/gene_EYR40_005141/gene_EYR40_010085/gene_EYR40_009051/gene_EYR40_006009/gene_EYR40_006397/gene_EYR40_010824/gene_EYR40_001841/gene_EY

KEGGID	Description	geneID	geneName	keggID	Count	Up	Up_Gene_id	Down	Down_Gene_id
		gene_EYR40_002485/gene_EYR40_000113/gene_EYR40_002733/gene_EYR40_006647/gene_EYR40_003186/gene_EYR40_005141/gene_EYR40_009740/gene_EYR40_010085/gene_EYR40_009051/gene_EYR40_006009/gene_EYR40_006397/gene_EYR40_002117/gene_EYR40_002707/gene_E		AT5G24400/ath:AT5G36700/ath:AT5G35360/ath:AT5G17330/ath:AT3G21110/ath:AT4G17190/ath:AT1G74540/ath:AT5G40850/ath:AT4G36250/ath:AT2G37790/ath:AT3G12900/ath:AT4G27270					R40_004207/gene_EYR40_000159/gene_EYR40_008942/gene_EYR40_002931/gene_EYR40_004961/gene_EYR40_007106/gene_EYR40_003302/gene_EYR40_006626/gene_EYR40_005016/gene_EYR40_000112/gene_EYR40_002752

KEGGID	Description	geneID	geneName	keggID	Count	Up	Up_Gene_id	Down	Down_Gene_id
		YR40_010824/gene_EYR40_001841/gene_EYR40_004207/gene_EYR40_000159/gene_EYR40_008942/gene_EYR40_002931/gene_EYR40_004961/gene_EYR40_002669/gene_EYR40_007106/gene_EYR40_003302/gene_EYR40_006626/gene_EYR40_005016/gene_EYR40_000112/gene_EYR40_0							

KEGGID	Description	geneID	geneName	keggID	Count	Up	Up_Gene_id	Down	Down_Gene_id
		02752							
ath00900	Terpenoid backbone biosynthesis	gene_EYR40_008586/gene_EYR40_002871/gene_EYR40_006409/gene_EYR40_005369/gene_EYR40_008361/gene_EYR40_007257/gene_EYR40_006647/gene_EYR40_009740/gene_EYR40_002669	EYR40_008586/HMG1/MVD1/ERG10/ERG20_2/BTS1/EYR40_006647/EYR40_009740/ERG20_1	ath:AT4G11820/ath:AT1G76490/ath:AT2G38700/ath:AT5G48230/ath:AT4G17190/ath:AT1G78510/ath:AT5G08335/ath:AT5G08335/ath:AT4G17190	9	4	gene_EYR40_008361/gene_EYR40_007257/gene_EYR40_009740/gene_EYR40_002669	5	gene_EYR40_008586/gene_EYR40_002871/gene_EYR40_006409/gene_EYR40_005369/gene_EYR40_006647
ath01200	Carbon metabolism	gene_EYR40_004142/gene_EYR40_000111/gene_EYR40_005655/gene_EYR40_00	EYR40_004142/ACS1/ZWF1/ERG10/CYS4/CYB5D1/EYR40_007985/EYR40_00866	ath:AT3G16910/ath:AT5G36880/ath:AT5G40760/ath:AT5G48230/ath:AT3G04940/at	21	1	gene_EYR40_009683	20	gene_EYR40_004142/gene_EYR40_000111/gene_EYR40_005655/gene_EYR40_00

KEGGID	Description	geneID	geneName	keggID	Count	Up	Up_Gene_id	Down	Down_Gene_id
		5369/gene_EYR40_008709/gene_EYR40_004126/gene_EYR40_007985/gene_EYR40_008668/gene_EYR40_005681/gene_EYR40_010251/gene_EYR40_009683/gene_EYR40_003938/gene_EYR40_001856/gene_EYR40_003896/gene_EYR40_006009/gene_EYR40_006397/gene_EYR40_001841/ge	8/GLK1/EYR40_010251/EYR40_009683/EYR40_003938/EYR40_001856/PYK1/CAT1/PGI1/6PGD/SOL1/EYR40_000159/PYC1/FDH1_2	h:AT3G14420/ath:AT2G42790/ath:AT3G59970/ath:AT2G19860/ath:AT4G16760/ath:AT2G19900/ath:AT1G17745/ath:AT1G20630/ath:AT5G63680/ath:AT1G20630/ath:AT5G42740/ath:AT5G41670/ath:AT5G24400/ath:AT5G36700/ath:AT5G35360/ath:AT5G14780					5369/gene_EYR40_008709/gene_EYR40_004126/gene_EYR40_007985/gene_EYR40_008668/gene_EYR40_005681/gene_EYR40_010251/gene_EYR40_003938/gene_EYR40_001856/gene_EYR40_003896/gene_EYR40_006009/gene_EYR40_006397/gene_EYR40_001841/gene_EYR40_004207/ge

KEGGID	Description	geneID	geneName	keggID	Count	Up	Up_Gene_id	Down	Down_Gene_id
		ne_EYR40_004207/gene_EYR40_000159/gene_EYR40_008942/gene_EYR40_006535							ne_EYR40_000159/gene_EYR40_008942/gene_EYR40_006535
ath00380	Tryptophan metabolism	gene_EYR40_001984/gene_EYR40_005369/gene_EYR40_001974/gene_EYR40_002476/gene_EYR40_001856/gene_EYR40_000419/novel.80/gene_EYR40_003763/gene_EYR40_006009/gene_EYR40_0	ALD5_1/ERG10/CHS2_1/EYR40_002476/EYR40_001856/EYR40_000419/-/EYR40_003763/CAT1/EYR40_006626	ath:AT1G23800/ath:AT5G48230/ath:AT1G23800/ath:AT4G35160/ath:AT1G20630/ath:AT4G34880/ath:AT4G31500/ath:AT1G08980/ath:AT1G20630/ath:AT4G36250	10	4	gene_EYR40_001974/gene_EYR40_002476/gene_EYR40_000419/novel.80	6	gene_EYR40_001984/gene_EYR40_005369/gene_EYR40_001856/gene_EYR40_003763/gene_EYR40_006009/gene_EYR40_006626

KEGGID	Description	geneID	geneName	keggID	Count	Up	Up_Gene_id	Down	Down_Gene_id
		06626							
ath00620	Pyruvate metabolism	gene_EYR40_004142/gene_EYR40_000111/gene_EYR40_001984/gene_EYR40_005369/gene_EYR40_001974/gene_EYR40_006294/gene_EYR40_009683/gene_EYR40_003896/gene_EYR40_010880/gene_EYR40_008942/gene_EYR40_006626/gene_EYR40_005016	EYR40_004142/ACS1/ALD5_1/ERG10/CHS2_1/GLO1/EYR40_009683/PYK1/EYR40_010880/PYC1/EYR40_006626/EYR40_005016	ath:AT3G16910/ath:AT5G36880/ath:AT1G23800/ath:AT5G48230/ath:AT1G23800/ath:AT1G08110/ath:AT2G19900/ath:AT5G63680/ath:AT2G37790/ath:AT5G35360/ath:AT4G36250/ath:AT2G37790	12	2	gene_EYR40_001974/gene_EYR40_009683	10	gene_EYR40_004142/gene_EYR40_000111/gene_EYR40_001984/gene_EYR40_005369/gene_EYR40_006294/gene_EYR40_003896/gene_EYR40_010880/gene_EYR40_008942/gene_EYR40_006626/gene_EYR40_005016

KEGG enrichment between CK vs A5 samples

KEGGID	Description	geneID	geneName	keggID	Count	Up	Up_Gene_id	Down	Down_Gene_id
ath03010	Ribosome	gene_EYR40_009786/gene_EYR40_008990/novel.72/gene_EYR40_006753/gene_EYR40_004739/gene_EYR40_008411/gene_EYR40_005367/gene_EYR40_007796/gene_EYR40_000160/gene_EYR40_005781/novel.987/novel.17/novel.672/gene_EYR40_004787/gene_EYR40_008890/gene_EYR40_0	RPL44/RPL30/-/RPL12/RPL19C/RPL8B/RPS1/RPP0/RPL34/RPS17/-/-/-/EYR40_004787/RPS4/RPL26B/RPL25/EYR40_003980/EYR40_006514/RPL43/EYR40_003344/EYR40_002395/RPL20/RPL3/EYR40_005275/RPL31/RPL5/RPS14/RPL35/RPS26/RPS9/RPL2A/EYR40_001823/RPS19/RP	ath:AT3G23390/ath:AT1G77940/ath:AT2G36170/ath:AT5G60670/ath:AT4G02230/ath:AT2G47610/ath:AT4G34670/ath:AT3G09200/ath:AT1G26880/ath:AT2G04390/ath:AT3G02190/ath:AT2G19750/ath:AT1G23410/ath:AT1G08360/ath:AT5G07090/ath:AT5G67510/ath:AT3G55280/ath:AT4G308	49	0		49	gene_EYR40_009786/gene_EYR40_008990/novel.72/gene_EYR40_006753/gene_EYR40_004739/gene_EYR40_008411/gene_EYR40_005367/gene_EYR40_007796/gene_EYR40_000160/gene_EYR40_005781/novel.987/novel.17/novel.672/gene_EYR40_004787/gene_EYR40_008890/gene_EYR40_0

KEGGID	Description	geneID	geneName	keggID	Count	Up	Up_Gene_id	Down	Down_Gene_id
		00981/gene_EYR40_009787/gene_EYR40_003980/gene_EYR40_006514/gene_EYR40_009075/gene_EYR40_003344/gene_EYR40_002395/gene_EYR40_000364/gene_EYR40_005589/gene_EYR40_005275/gene_EYR40_000166/gene_EYR40_005223/gene_EYR40_001824/gene_EYR40_005642/	S16/EYR40_004866/RPL11/RPL13/RPS29/EYR40_004013/EYR40_005726/-/RPL28/EYR40_005227/RPS22/RPS2/EYR40_006263/EYR40_004858/RPS15	00/ath:AT5G59240/ath:AT3G60245/ath:AT1G09690/ath:AT3G10090/ath:AT1G29965/ath:AT1G61580/ath:AT4G10450/ath:AT5G56710/ath:AT5G39740/ath:AT2G36160/ath:AT3G55170/ath:AT2G40590/ath:AT5G15200/ath:AT2G18020/ath:AT3G11510/ath:AT3G02080/ath:AT5G18380/ath:AT2G2					00981/gene_EYR40_009787/gene_EYR40_003980/gene_EYR40_006514/gene_EYR40_009075/gene_EYR40_003344/gene_EYR40_002395/gene_EYR40_000364/gene_EYR40_005589/gene_EYR40_005275/gene_EYR40_000166/gene_EYR40_005223/gene_EYR40_001824/gene_EYR40_005642/

KEGGID	Description	geneID	geneName	keggID	Count	Up	Up_Gene_id	Down	Down_Gene_id
		gene_EYR40_006315/gene_EYR40_003343/gene_EYR40_005093/gene_EYR40_001823/gene_EYR40_003570/gene_EYR40_003941/gene_EYR40_004866/gene_EYR40_008565/gene_EYR40_003934/gene_EYR40_004430/gene_EYR40_004013/gene_EYR40_005726/novel.410/gene_EYR40_01085		0450/ath:AT2G42740/ath:AT3G49010/ath:AT3G43980/ath:AT3G04920/ath:AT5G27850/ath:AT2G37270/ath:AT1G70600/ath:AT5G52650/ath:AT1G07770/ath:AT1G58983/ath:AT1G04480/ath:AT2G32060/ath:AT1G04270					gene_EYR40_006315/gene_EYR40_003343/gene_EYR40_005093/gene_EYR40_001823/gene_EYR40_003570/gene_EYR40_003941/gene_EYR40_004866/gene_EYR40_008565/gene_EYR40_003934/gene_EYR40_004430/gene_EYR40_004013/gene_EYR40_005726/novel.410/gene_EYR40_01085

KEGGID	Description	geneID	geneName	keggID	Count	Up	Up_Gene_id	Down	Down_Gene_id
		6/gene_EYR40_005227/gene_EYR40_005782/gene_EYR40_006651/gene_EYR40_006263/gene_EYR40_004858/gene_EYR40_008794							6/gene_EYR40_005227/gene_EYR40_005782/gene_EYR40_006651/gene_EYR40_006263/gene_EYR40_004858/gene_EYR40_008794
ath01110	Biosynthesis of secondary metabolites	gene_EYR40_002735/gene_EYR40_001191/gene_EYR40_006780/gene_EYR40_001974/gene_EYR40_005369/gene_EYR40_007257/gene_EYR40_008609	EYR40_002735/NDK1/PCK1_1/CHS2_1/ERG10/BTS1/PAN6/EYR40_003404/TAL1_2/ADH1_2/IDI1/PDC1/ACL1/EYR40_004142/EYR40_003449/HIS5/E	ath:AT1G24360/ath:AT4G09320/ath:AT4G37870/ath:AT1G23800/ath:AT5G48230/ath:AT1G78510/ath:AT5G48840/ath:AT1G74030/ath:AT1G12230/ath:	98	44	gene_EYR40_002735/gene_EYR40_005369/gene_EYR40_008609/gene_EYR40_003985/gene_EYR40_007898/gene_EYR40_004142/gene_EYR40_008361	54	gene_EYR40_001191/gene_EYR40_006780/gene_EYR40_001974/gene_EYR40_007257/gene_EYR40_003404/gene_EYR40_009230/gene_EYR40_010824

KEGGID	Description	geneID	geneName	keggID	Count	Up	Up_Gene_id	Down	Down_Gene_id
		/gene_EYR40_003404/gene_EYR40_009230/gene_EYR40_010824/gene_EYR40_003985/gene_EYR40_009513/gene_EYR40_007898/gene_EYR40_004142/gene_EYR40_003449/gene_EYR40_002845/gene_EYR40_008361/gene_EYR40_008395/gene_EYR40_010175/gene_EYR40_011013/gene_	RG20_2/ERG1_1/EYR40_010175/TPS2/HEM13/ERG7/EYR40_002629/MVD1/LEU2/EYR40_006810/EYR40_000721/EYR40_010752/EYR40_008586/EYR40_008120/GLC3/EYR40_009302/EYR40_003804/TPS1/EYR40_004481/EYR40_001570/ERG24_2/EYR40_010439/ERG24_1/EYR40_009514/PMI1	AT4G37980/ath:AT3G02780/ath:AT5G01320/ath:AT3G06650/ath:AT3G16910/ath:AT4G13010/ath:AT1G71920/ath:AT4G17190/ath:AT1G58440/ath:AT5G07990/ath:AT1G78580/ath:AT1G03475/ath:AT2G07050/ath:AT4G17260/ath:AT2G38700/ath:AT1G31180/ath:AT2G20340/ath:AT5G11520/a			/gene_EYR40_008395/gene_EYR40_010175/gene_EYR40_009845/gene_EYR40_006409/gene_EYR40_006810/gene_EYR40_008586/gene_EYR40_008120/gene_EYR40_001570/gene_EYR40_008403/gene_EYR40_007981/gene_EYR40_009514/gene_EYR40_000905/novel.610/gene_EYR40_01071		/gene_EYR40_009513/gene_EYR40_003449/gene_EYR40_002845/gene_EYR40_011013/gene_EYR40_002971/gene_EYR40_002629/gene_EYR40_005141/gene_EYR40_000721/gene_EYR40_010752/gene_EYR40_005153/gene_EYR40_009302/gene_EYR40_003804/gene_EYR40_007797/gene_

KEGGID	Description	geneID	geneName	keggID	Count	Up	Up_Gene_id	Down	Down_Gene_id
		EYR40_002971/gene_EYR40_009845/gene_EYR40_002629/gene_EYR40_006409/gene_EYR40_005141/gene_EYR40_006810/gene_EYR40_000721/gene_EYR40_010752/gene_EYR40_008586/gene_EYR40_008120/gene_EYR40_005153/gene_EYR40_009302/gene_EYR40_003804/gene_EYR40_	/SAM2/-/ERG6/EYR40_005882/EYR40_001856/ERG11/EYR40_008786/FBP1/UGP1_2/HIS7/EYR40_007776/TRP4/EYR40_006900/EYR40_000613/EYR40_009367/ADE10/EYR40_000113/CYB2_2/ERG9/EYR40_009275/ACS1/ERG4/EYR40_010481/SOL1/6PGD/ALTA7/PGM2/ERG26/EYR40	th:AT1G31230/ath:AT4G11820/ath:AT5G07990/ath:AT2G36390/ath:AT5G03690/ath:AT1G65060/ath:AT1G78580/ath:AT5G06060/ath:AT5G11720/ath:AT3G52940/ath:AT1G79530/ath:AT3G52940/ath:AT1G74550/ath:AT3G02570/ath:AT2G36880/ath:AT4G34640/ath:AT5G13710/ath:AT1G74550			1/gene_EYR40_005882/gene_EYR40_008002/gene_EYR40_006900/gene_EYR40_000113/gene_EYR40_007083/gene_EYR40_000111/gene_EYR40_008651/gene_EYR40_009498/gene_EYR40_009678/gene_EYR40_000112/gene_EYR40_003881/gene_EYR40_002871/gene_EYR40_009429/gene		EYR40_004481/gene_EYR40_010439/gene_EYR40_008405/gene_EYR40_001856/gene_EYR40_008786/gene_EYR40_000810/gene_EYR40_006516/gene_EYR40_003937/gene_EYR40_007776/gene_EYR40_003086/gene_EYR40_000613/gene_EYR40_009367/gene_EYR40_009051/gene_EYR40_

KEGGID	Description	geneID	geneName	keggID	Count	Up	Up_Gene_id	Down	Down_Gene_id
		007797/gene_EYR40_004481/gene_EYR40_001570/gene_EYR40_008403/gene_EYR40_010439/gene_EYR40_007981/gene_EYR40_009514/gene_EYR40_008405/gene_EYR40_000905/novel.610/gene_EYR40_010711/gene_EYR40_005882/gene_EYR40_001856/gene_EYR40_008002/gene_EYR	_009678/EYR40_000112/EYR40_003881/HMG1/ARO7/EYR40_009864/TAL1_1/ADH1_1/TKL1/PGK1/RPE1/HOM3_2/FDH1_1/DOR14/ADE1/CAR2/EYR40_003417/EYR40_008264/HOM3_1/CAT1/EYR40_004907/EYR40_003799/PYC1/EYR40_010085/PGI1/EYR40_007545/EYR40_002485/EYR40	/ath:AT1G20630/ath:AT1G11680/ath:AT5G08335/ath:AT1G43670/ath:AT3G03250/ath:AT4G26900/ath:AT1G76680/ath:AT5G17990/ath:AT3G53260/ath:AT3G08590/ath:AT3G48560/ath:AT2G35040/ath:AT3G12900/ath:AT3G14415/ath:AT4G34640/ath:AT1G24360/ath:AT5G36880/ath:AT3G529			_EYR40_001564/gene_EYR40_003137/gene_EYR40_010324/gene_EYR40_008264/gene_EYR40_003130/gene_EYR40_004907/gene_EYR40_003799/gene_EYR40_007545/gene_EYR40_002485/gene_EYR40_006647		009846/gene_EYR40_009275/gene_EYR40_010481/gene_EYR40_004207/gene_EYR40_001841/gene_EYR40_002752/gene_EYR40_008608/gene_EYR40_009864/gene_EYR40_001028/gene_EYR40_001125/gene_EYR40_001182/gene_EYR40_003898/gene_EYR40_005558/gene_EYR40_004536

KEGGID	Description	geneID	geneName	keggID	Count	Up	Up_Gene_id	Down	Down_Gene_id
		40_008786/gene_EYR40_000810/gene_EYR40_006516/gene_EYR40_003937/gene_EYR40_007776/gene_EYR40_003086/gene_EYR40_006900/gene_EYR40_000613/gene_EYR40_009367/gene_EYR40_009051/gene_EYR40_000113/gene_EYR40_007083/gene_EYR40_009846/gene_EYR40_009	_006626/EYR40_006647	40/ath:AT1G79530/ath:AT5G24400/ath:AT5G41670/ath:AT4G27270/ath:AT5G51820/ath:AT2G26260/ath:AT4G24040/ath:AT3G12900/ath:AT5G06060/ath:AT1G76490/ath:AT3G29200/ath:AT2G24850/ath:AT1G12230/ath:AT4G37990/ath:AT2G45290/ath:AT1G79550/ath:AT1G63290/ath:AT3G0					/gene_EYR40_004961/gene_EYR40_003417/gene_EYR40_006009/gene_EYR40_008942/gene_EYR40_010085/gene_EYR40_006397/gene_EYR40_006626

KEGGID	Description	geneID	geneName	keggID	Count	Up	Up_Gene_id	Down	Down_Gene_id
		275/gene_EYR40_000111/gene_EYR40_008651/gene_EYR40_010481/gene_EYR40_004207/gene_EYR40_001841/gene_EYR40_002752/gene_EYR40_008608/gene_EYR40_009498/gene_EYR40_009678/gene_EYR40_000112/gene_EYR40_003881/gene_EYR40_002871/gene_EYR40_009429/gen		2020/ath:AT5G43940/ath:AT5G41670/ath:AT3G21110/ath:AT5G46180/ath:AT3G02870/ath:AT1G74540/ath:AT5G13280/ath:AT1G20630/ath:AT1G76690/ath:AT4G05160/ath:AT5G35360/ath:AT2G06050/ath:AT5G42740/ath:AT3G08860/ath:AT1G73050/ath:AT4G36250/ath:AT5G08335					

KEGGID	Description	geneID	geneName	keggID	Count	Up	Up_Gene_id	Down	Down_Gene_id
		e_EYR40_009864/gene_EYR40_001028/gene_EYR40_001564/gene_EYR40_001125/gene_EYR40_001182/gene_EYR40_003898/gene_EYR40_003137/gene_EYR40_005558/gene_EYR40_004536/gene_EYR40_004961/gene_EYR40_010324/gene_EYR40_003417/gene_EYR40_008264/gene_EYR							

KEGGID	Description	geneID	geneName	keggID	Count	Up	Up_Gene_id	Down	Down_Gene_id
		40_003130/gene_EYR40_006009/gene_EYR40_004907/gene_EYR40_003799/gene_EYR40_008942/gene_EYR40_010085/gene_EYR40_006397/gene_EYR40_007545/gene_EYR40_002485/gene_EYR40_006626/gene_EYR40_006647							
ath00030	Pentose phosphate pathway	gene_EYR40_009230/gene_EYR40_009302/gene_EYR40_0	TAL1_2/EYR40_009302/FBP1/SOL1/6PGD/PGM2/TAL1_1	ath:AT1G12230/ath:AT5G03690/ath:AT1G43670/ath:AT5	11	0		11	gene_EYR40_009230/gene_EYR40_009302/gene_EYR40_0

KEGGID	Description	geneID	geneName	keggID	Count	Up	Up_Gene_id	Down	Down_Gene_id
		00810/gene_EYR40_004207/gene_EYR40_001841/gene_EYR40_008608/gene_EYR40_001028/gene_EYR40_001125/gene_EYR40_003898/gene_EYR40_004536/gene_EYR40_006397	/TKL1/RPE1/DOR14/PGI1	G24400/ath:AT5G41670/ath:AT5G51820/ath:AT1G12230/ath:AT2G45290/ath:AT1G63290/ath:AT5G41670/ath:AT5G42740					00810/gene_EYR40_004207/gene_EYR40_001841/gene_EYR40_008608/gene_EYR40_001028/gene_EYR40_001125/gene_EYR40_003898/gene_EYR40_004536/gene_EYR40_006397
ath03030	DNA replication	gene_EYR40_001082/gene_EYR40_000562/gene_EYR40_001920/gene_EYR40_000744/gene_EYR40_002	EYR40_001082/MCM7/POL2/EYR40_000744/RFA1_2/EYR40_003982/RFA1_1/MCM3/POL3/MCM5/EY	ath:AT1G21690/ath:AT4G02060/ath:AT1G08260/ath:AT1G67320/ath:AT4G19130/ath:AT1G08130/ath:A	15	14	gene_EYR40_001082/gene_EYR40_000562/gene_EYR40_001920/gene_EYR40_000744/gene_EYR40_003	1	gene_EYR40_002255

KEGGID	Description	geneID	geneName	keggID	Count	Up	Up_Gene_id	Down	Down_Gene_id
		255/gene_EYR40_003982/gene_EYR40_000883/gene_EYR40_004337/gene_EYR40_008958/gene_EYR40_004298/gene_EYR40_000252/gene_EYR40_010764/gene_EYR40_008614/gene_EYR40_008110/gene_EYR40_000153	R40_000252/EYR40_010764/RFC3/RFC5/POL30	T5G61000/ath:AT5G46280/ath:AT5G63960/ath:AT2G07690/ath:AT2G16440/ath:AT5G41880/ath:AT1G77470/ath:AT5G27740/ath:AT2G29570			982/gene_EYR40_000883/gene_EYR40_004337/gene_EYR40_008958/gene_EYR40_004298/gene_EYR40_000252/gene_EYR40_010764/gene_EYR40_008614/gene_EYR40_008110/gene_EYR40_000153		
ath00100	Steroid biosynthesis	gene_EYR40_008395/gene_EYR40_009845/gene_EYR40_008403/gene	ERG1_1/ERG7/ERG24_2/ERG24_1/-/ERG6/ERG	ath:AT1G58440/ath:AT2G07050/ath:AT3G52940/ath:AT3G52940/ath:	10	9	gene_EYR40_008395/gene_EYR40_009845/gene_EYR40_008403/gene	1	gene_EYR40_009846

KEGGID	Description	geneID	geneName	keggID	Count	Up	Up_Gene_id	Down	Down_Gene_id
		_EYR40_007981/novel.610/gene_EYR40_010711/gene_EYR40_008002/gene_EYR40_009846/gene_EYR40_008651/gene_EYR40_009498	11/ERG9/ERG4/ERG26	AT4G34640/ath:AT5G13710/ath:AT1G11680/ath:AT4G34640/ath:AT3G52940/ath:AT2G26260			_EYR40_007981/novel.610/gene_EYR40_010711/gene_EYR40_008002/gene_EYR40_008651/gene_EYR40_009498		
ath00010	Glycolysis / Gluconeogenesis	gene_EYR40_006780/gene_EYR40_001974/gene_EYR40_003404/gene_EYR40_009513/gene_EYR40_004142/gene_EYR40_002629/gene_EYR40_009302	PCK1_1/CHS2_1/EYR40_003404/PDC1/EYR40_004142/EYR40_002629/EYR40_009302/EYR40_010439/FBP1/EYR40_000613/ACS1/EYR40_010481/PGM2	ath:AT4G37870/ath:AT1G23800/ath:AT1G74030/ath:AT5G01320/ath:AT3G16910/ath:AT4G17260/ath:AT5G03690/ath:AT1G79530/ath:AT1G43670/ath:	17	2	gene_EYR40_004142/gene_EYR40_000111	15	gene_EYR40_006780/gene_EYR40_001974/gene_EYR40_003404/gene_EYR40_009513/gene_EYR40_002629/gene_EYR40_009302/gene_EYR40_010439

KEGGID	Description	geneID	geneName	keggID	Count	Up	Up_Gene_id	Down	Down_Gene_id
		/gene_EYR40_010439/gene_EYR40_000810/gene_EYR40_000613/gene_EYR40_000111/gene_EYR40_010481/gene_EYR40_008608/gene_EYR40_001182/gene_EYR40_005558/gene_EYR40_006397/gene_EYR40_006626	/PGK1/FDH1_1/PGI1/EYR40_006626	AT3G08590/ath:AT5G36880/ath:AT1G79530/ath:AT5G51820/ath:AT1G79550/ath:AT5G43940/ath:AT5G42740/ath:AT4G36250					/gene_EYR40_000810/gene_EYR40_000613/gene_EYR40_010481/gene_EYR40_008608/gene_EYR40_001182/gene_EYR40_005558/gene_EYR40_006397/gene_EYR40_006626
ath03430	Mismatch repair	gene_EYR40_001082/gene_EYR40_010696/gene_EYR40_002255/gene	EYR40_001082/MSH6/RFA1_2/EYR40_003982/RFA1_1/POL3/MSH3/	ath:AT1G21690/ath:AT3G24495/ath:AT4G19130/ath:AT1G08130/ath:	10	9	gene_EYR40_001082/gene_EYR40_010696/gene_EYR40_003982/gene	1	gene_EYR40_002255

KEGGID	Description	geneID	geneName	keggID	Count	Up	Up_Gene_id	Down	Down_Gene_id
		_EYR40_003982/gene_EYR40_000883/gene_EYR40_008958/gene_EYR40_000688/gene_EYR40_008614/gene_EYR40_008110/gene_EYR40_000153	RFC3/RFC5/POL30	AT5G61000/ath:AT5G63960/ath:AT4G25540/ath:AT1G77470/ath:AT5G27740/ath:AT2G29570			_EYR40_000883/gene_EYR40_008958/gene_EYR40_000688/gene_EYR40_008614/gene_EYR40_008110/gene_EYR40_000153		
ath00900	Terpenoid backbone biosynthesis	gene_EYR40_005369/gene_EYR40_007257/gene_EYR40_003985/gene_EYR40_008361/gene_EYR40_006409/gene_EYR40_008586/gene_EY	ERG10/BTS1/IDI1/ERG20_2/MVD1/EYR40_008586/EYR40_008786/HMG1/EYR40_006647	ath:AT5G48230/ath:AT1G78510/ath:AT3G02780/ath:AT4G17190/ath:AT2G38700/ath:AT4G11820/ath:AT5G08335/ath:AT1G764	9	7	gene_EYR40_005369/gene_EYR40_003985/gene_EYR40_008361/gene_EYR40_006409/gene_EYR40_008586/gene_EYR40_00287	2	gene_EYR40_007257/gene_EYR40_008786

KEGGID	Description	geneID	geneName	keggID	Count	Up	Up_Gene_id	Down	Down_Gene_id
		R40_008786/gene_EYR40_002871/gene_EYR40_006647		90/ath:AT5G08335			1/gene_EYR40_006647		
ath01200	Carbon metabolism	gene_EYR40_006780/gene_EYR40_005369/gene_EYR40_003404/gene_EYR40_009230/gene_EYR40_004142/gene_EYR40_000721/gene_EYR40_009302/gene_EYR40_010439/gene_EYR40_001856/gene_EYR40_000810/gene_EYR40_0	PCK1_1/ERG10/EYR40_003404/TAL1_2/EYR40_004142/EYR40_000721/EYR40_009302/EYR40_010439/EYR40_001856/FBP1/EYR40_000613/CYB2_2/ACS1/EYR40_010481/SOL1/6PGD/EYR40_001259/TAL1_1/TKL1/PGK1/RPE1/FDH1_	ath:AT4G37870/ath:AT5G48230/ath:AT1G74030/ath:AT1G12230/ath:AT3G16910/ath:AT5G11520/ath:AT5G03690/ath:AT1G79530/ath:AT1G20630/ath:AT1G43670/ath:AT3G08590/ath:AT3G14415/ath:AT5G36880/ath:AT1G79530/ath:	27	5	gene_EYR40_005369/gene_EYR40_004142/gene_EYR40_007083/gene_EYR40_000111/gene_EYR40_001259	22	gene_EYR40_006780/gene_EYR40_003404/gene_EYR40_009230/gene_EYR40_000721/gene_EYR40_009302/gene_EYR40_010439/gene_EYR40_001856/gene_EYR40_000810/gene_EYR40_000613/gene_EYR40_010481/gene_EYR40_0

KEGGID	Description	geneID	geneName	keggID	Count	Up	Up_Gene_id	Down	Down_Gene_id
		00613/gene_EYR40_007083/gene_EYR40_000111/gene_EYR40_010481/gene_EYR40_004207/gene_EYR40_001841/gene_EYR40_001259/gene_EYR40_001028/gene_EYR40_001125/gene_EYR40_001182/gene_EYR40_003898/gene_EYR40_005558/gene_EYR40_004536/gene_EYR40_003819/	1/DOR14/EYR40_003819/CAT1/PYC1/PGI1	AT5G24400/ath:AT5G41670/ath:AT4G20930/ath:AT1G12230/ath:AT2G45290/ath:AT1G79550/ath:AT1G63290/ath:AT5G43940/ath:AT5G41670/ath:AT2G41530/ath:AT1G20630/ath:AT5G35360/ath:AT5G42740					04207/gene_EYR40_001841/gene_EYR40_001028/gene_EYR40_001125/gene_EYR40_001182/gene_EYR40_003898/gene_EYR40_005558/gene_EYR40_004536/gene_EYR40_003819/gene_EYR40_006009/gene_EYR40_008942/gene_EYR40_006397

KEGGID	Description	geneID	geneName	keggID	Count	Up	Up_Gene_id	Down	Down_Gene_id
		gene_EYR40_006009/gene_EYR40_008942/gene_EYR40_006397							
ath00710	Carbon fixation in photosynthetic organisms	gene_EYR40_006780/gene_EYR40_000721/gene_EYR40_009302/gene_EYR40_010439/gene_EYR40_000810/gene_EYR40_010481/gene_EYR40_001125/gene_EYR40_001182/gene_EYR40_003898	PCK1_1/EYR40_000721/EYR40_009302/EYR40_010439/FBP1/EYR40_010481/TKL1/PGK1/RPE1	ath:AT4G37870/ath:AT5G11520/ath:AT5G03690/ath:AT1G79530/ath:AT1G43670/ath:AT1G79530/ath:AT2G45290/ath:AT1G79550/ath:AT1G63290	9	0		9	gene_EYR40_006780/gene_EYR40_000721/gene_EYR40_009302/gene_EYR40_010439/gene_EYR40_000810/gene_EYR40_010481/gene_EYR40_001125/gene_EYR40_001182/gene_EYR40_003898
**KEGG enrichment between B5 and CK samples**									
ath01110	Biosynthesi	gene_EYR4	ADH1_2/E	ath:AT4G37	94	23	gene_EYR4	71	gene_EYR4

KEGGID	Description	geneID	geneName	keggID	Count	Up	Up_Gene_id	Down	Down_Gene_id
	s of secondary metabolites	0_010824/gene_EYR40_006626/gene_EYR40_006900/gene_EYR40_005655/gene_EYR40_010880/gene_EYR40_002707/gene_EYR40_010089/gene_EYR40_006516/gene_EYR40_010439/gene_EYR40_009513/gene_EYR40_001191/gene_EYR40_002439/gene_EYR40_001984/gene_EYR40_00708	YR40_006626/EYR40_006900/ZWF1/EYR40_010880/EYR40_002707/GPH1/UGP1_2/EYR40_010439/PDC1/NDK1/EYR40_002439/ALD5_1/CYB2_2/LEU2/EYR40_001793/EYR40_003404/EYR40_001458/PGM2/GLC3/MET6/6PGD/PCK1_1/EYR40_000721/EYR40_002629/EYR40_009514/SOL1/HE	980/ath:AT4G36250/ath:AT3G53260/ath:AT5G40760/ath:AT2G37790/ath:AT3G03980/ath:AT3G46970/ath:AT3G03250/ath:AT1G79530/ath:AT5G01320/ath:AT4G09320/ath:AT1G48130/ath:AT1G23800/ath:AT3G14415/ath:AT1G31180/ath:AT5G07990/ath:AT1G74030/ath:AT1G74540/ath:AT5G5			0_006900/gene_EYR40_002707/gene_EYR40_007083/gene_EYR40_001458/gene_EYR40_009514/gene_EYR40_008120/gene_EYR40_006989/gene_EYR40_005882/gene_EYR40_009573/gene_EYR40_002485/gene_EYR40_002476/novel.80/gene_EYR40_001570/gene_EYR40_010324/gene_EYR		0_010824/gene_EYR40_006626/gene_EYR40_005655/gene_EYR40_010880/gene_EYR40_010089/gene_EYR40_006516/gene_EYR40_010439/gene_EYR40_009513/gene_EYR40_001191/gene_EYR40_002439/gene_EYR40_001984/gene_EYR40_005141/gene_EYR40_001793/gene_EYR40_00340

KEGGID	Description	geneID	geneName	keggID	Count	Up	Up_Gene_id	Down	Down_Gene_id
		3/gene_EYR40_005141/gene_EYR40_001793/gene_EYR40_003404/gene_EYR40_001458/gene_EYR40_008608/gene_EYR40_005153/gene_EYR40_005259/gene_EYR40_001841/gene_EYR40_006780/gene_EYR40_000721/gene_EYR40_002629/gene_EYR40_009514/gene_EYR40_004207/gene	M13/ALTA7/FDH1_1/EYR40_010868/EYR40_007985/GLK1/CAT1/EYR40_000613/PGI1/EYR40_010481/EYR40_008120/PYK1/TAL1_2/PYC1/ERG8/DOR14/TAL1_1/PMI1/EYR40_005882/ADE10/TKL1/EYR40_003938/EYR40_004318/ARG4/TRP5/TPS1/ARG1/ALD5_2/MVD1/PGK1/EYR40_	1820/ath:AT2G36390/ath:AT5G17920/ath:AT5G41670/ath:AT4G37870/ath:AT5G11520/ath:AT4G17260/ath:AT1G74550/ath:AT5G24400/ath:AT1G03475/ath:AT4G27270/ath:AT5G43940/ath:AT1G05160/ath:AT2G42790/ath:AT2G19860/ath:AT1G20630/ath:AT3G08590/ath:AT5G42740/ath:AT1			40_008361/gene_EYR40_001814/gene_EYR40_008592/gene_EYR40_007082/gene_EYR40_003799/gene_EYR40_003130/gene_EYR40_003137/gene_EYR40_002735/gene_EYR40_004907		4/gene_EYR40_008608/gene_EYR40_005153/gene_EYR40_005259/gene_EYR40_001841/gene_EYR40_006780/gene_EYR40_000721/gene_EYR40_002629/gene_EYR40_004207/gene_EYR40_002971/gene_EYR40_002752/gene_EYR40_005558/gene_EYR40_010868/gene_EYR40_007985/gene

KEGGID	Description	geneID	geneName	keggID	Count	Up	Up_Gene_id	Down	Down_Gene_id
		_EYR40_002971/gene_EYR40_002752/gene_EYR40_005558/gene_EYR40_010868/gene_EYR40_007985/gene_EYR40_005681/gene_EYR40_006009/gene_EYR40_000613/gene_EYR40_006397/gene_EYR40_010481/gene_EYR40_008120/gene_EYR40_003896/gene_EYR40_009230/gene_EYR4	009573/HEM12/EYR40_007776/EYR40_002485/EYR40_002476/PRO3/-/LEU1/EYR40_009302/TPS2/EYR40_001570/ADE1/EYR40_010752/EYR40_003103/EYR40_006210/CAR2/EYR40_000886/ERG20_2/EYR40_001814/EYR40_009864/EYR40_008592/EYR40_000159/EYR40_002931/EYR40_001983	G79530/ath:AT5G07990/ath:AT5G63680/ath:AT1G12230/ath:AT5G35360/ath:AT1G31910/ath:AT5G41670/ath:AT1G12230/ath:AT3G02570/ath:AT1G74550/ath:AT2G35040/ath:AT2G45290/ath:AT1G17745/ath:AT4G39980/ath:AT5G10920/ath:AT4G27070/ath:AT1G78580/ath:AT4G24830/ath:AT					_EYR40_005681/gene_EYR40_006009/gene_EYR40_000613/gene_EYR40_006397/gene_EYR40_010481/gene_EYR40_003896/gene_EYR40_009230/gene_EYR40_008942/gene_EYR40_004536/gene_EYR40_001028/gene_EYR40_008405/gene_EYR40_009051/gene_EYR40_001125/gene_EYR4

KEGGID	Description	geneID	geneName	keggID	Count	Up	Up_Gene_id	Down	Down_Gene_id
		0_008942/gene_EYR40_006989/gene_EYR40_004536/gene_EYR40_001028/gene_EYR40_008405/gene_EYR40_005882/gene_EYR40_009051/gene_EYR40_001125/gene_EYR40_003938/gene_EYR40_004318/gene_EYR40_000718/gene_EYR40_000921/gene_EYR40_007797/gene_EYR40_00881	/CYB2_1/EYR40_003417/EYR40_003799/EYR40_002283/HOM3_1/EYR40_005016/EYR40_002733/EYR40_007165/EYR40_001133/HOM3_2/EYR40_002735/EYR40_004481/EYR40_004907	3G24503/ath:AT2G38700/ath:AT1G79550/ath:AT1G21400/ath:AT2G40490/ath:AT1G76680/ath:AT1G73050/ath:AT4G35160/ath:AT5G14800/ath:AT4G31500/ath:AT4G13430/ath:AT5G03690/ath:AT1G78580/ath:AT5G11720/ath:AT3G21110/ath:AT1G31230/ath:AT1G74540/ath:AT1G74260/ath:					0_003938/gene_EYR40_004318/gene_EYR40_000718/gene_EYR40_000921/gene_EYR40_007797/gene_EYR40_008814/gene_EYR40_005381/gene_EYR40_006409/gene_EYR40_001182/gene_EYR40_005075/gene_EYR40_007776/gene_EYR40_000701/gene_EYR40_005436/gene_EYR40_00930

KEGGID	Description	geneID	geneName	keggID	Count	Up	Up_Gene_id	Down	Down_Gene_id
		4/gene_EYR40_005381/gene_EYR40_006409/gene_EYR40_001182/gene_EYR40_009573/gene_EYR40_005075/gene_EYR40_007776/gene_EYR40_002485/gene_EYR40_002476/gene_EYR40_000701/novel.80/gene_EYR40_005436/gene_EYR40_009302/gene_EYR40_011013/gene_EYR40_00		AT5G46180/ath:AT5G26710/ath:AT4G17190/ath:AT5G07990/ath:AT2G24850/ath:AT3G02600/ath:AT5G36700/ath:AT5G17330/ath:AT5G07990/ath:AT3G14415/ath:AT3G02870/ath:AT4G05160/ath:AT1G07440/ath:AT5G13280/ath:AT2G37790/ath:AT3G57050/ath:AT3G61530/ath:AT1G65960/at					2/gene_EYR40_011013/gene_EYR40_004961/gene_EYR40_010752/gene_EYR40_003103/gene_EYR40_006210/gene_EYR40_000886/gene_EYR40_009864/gene_EYR40_000159/gene_EYR40_002931/gene_EYR40_001983/gene_EYR40_003417/gene_EYR40_002283/gene_EYR40_005016/gene

KEGGID	Description	geneID	geneName	keggID	Count	Up	Up_Gene_id	Down	Down_Gene_id
		1570/gene_EYR40_004961/gene_EYR40_010752/gene_EYR40_003103/gene_EYR40_006210/gene_EYR40_010324/gene_EYR40_000886/gene_EYR40_008361/gene_EYR40_001814/gene_EYR40_009864/gene_EYR40_008592/gene_EYR40_000159/gene_EYR40_002931/gene_EYR40_001983/ge		h:AT3G02020/ath:AT1G24360/ath:AT5G06060/ath:AT1G76690					_EYR40_002733/gene_EYR40_007165/gene_EYR40_001133/gene_EYR40_004481

KEGGID	Description	geneID	geneName	keggID	Count	Up	Up_Gene_id	Down	Down_Gene_id
		ne_EYR40_007082/gene_EYR40_003417/gene_EYR40_003799/gene_EYR40_002283/gene_EYR40_003130/gene_EYR40_005016/gene_EYR40_002733/gene_EYR40_007165/gene_EYR40_001133/gene_EYR40_003137/gene_EYR40_002735/gene_EYR40_004481/gene_EYR40_004907							

KEGGID	Description	geneID	geneName	keggID	Count	Up	Up_Gene_id	Down	Down_Gene_id
ath01200	Carbon metabolism	gene_EYR40_005655/gene_EYR40_010439/gene_EYR40_007083/gene_EYR40_003404/gene_EYR40_001841/gene_EYR40_006780/gene_EYR40_000721/gene_EYR40_004207/gene_EYR40_005558/gene_EYR40_007985/gene_EYR40_005681/gene_EYR40_006009/gene_EYR40_000613/gene_E	ZWF1/EYR40_010439/CYB2_2/EYR40_003404/6PGD/PCK1_1/EYR40_000721/SOL1/FDH1_1/EYR40_007985/GLK1/CAT1/EYR40_000613/PGI1/EYR40_010481/PYK1/TAL1_2/PYC1/DOR14/TAL1_1/TKL1/EYR40_003938/PGK1/EYR40_009302/EYR40_003819/EYR40_001259/EYR40_000159/FD	ath:AT5G40760/ath:AT1G79530/ath:AT3G14415/ath:AT1G74030/ath:AT5G41670/ath:AT4G37870/ath:AT5G11520/ath:AT5G24400/ath:AT5G43940/ath:AT2G42790/ath:AT2G19860/ath:AT1G20630/ath:AT3G08590/ath:AT5G42740/ath:AT1G79530/ath:AT5G63680/ath:AT1G12230/ath:AT5G35360	30	4	gene_EYR40_007083/gene_EYR40_001259/gene_EYR40_007082/gene_EYR40_009683	26	gene_EYR40_005655/gene_EYR40_010439/gene_EYR40_003404/gene_EYR40_001841/gene_EYR40_006780/gene_EYR40_000721/gene_EYR40_004207/gene_EYR40_005558/gene_EYR40_007985/gene_EYR40_005681/gene_EYR40_006009/gene_EYR40_000613/gene_EYR40_006397/gene_E

KEGGID	Description	geneID	geneName	keggID	Count	Up	Up_Gene_id	Down	Down_Gene_id
		YR40_006397/gene_EYR40_010481/gene_EYR40_003896/gene_EYR40_009230/gene_EYR40_008942/gene_EYR40_004536/gene_EYR40_001028/gene_EYR40_001125/gene_EYR40_003938/gene_EYR40_001182/gene_EYR40_009302/gene_EYR40_003819/gene_EYR40_001259/gene_EYR40_0	H1_2/CYB2_1/EYR40_009683	/ath:AT5G41670/ath:AT1G12230/ath:AT2G45290/ath:AT1G17745/ath:AT1G79550/ath:AT5G03690/ath:AT2G41530/ath:AT4G20930/ath:AT5G36700/ath:AT5G14780/ath:AT3G14415/ath:AT2G19900					YR40_010481/gene_EYR40_003896/gene_EYR40_009230/gene_EYR40_008942/gene_EYR40_004536/gene_EYR40_001028/gene_EYR40_001125/gene_EYR40_003938/gene_EYR40_001182/gene_EYR40_009302/gene_EYR40_003819/gene_EYR40_000159/gene_EYR40_006535

KEGGID	Description	geneID	geneName	keggID	Count	Up	Up_Gene_id	Down	Down_Gene_id
		00159/gene_EYR40_006535/gene_EYR40_007082/gene_EYR40_009683							
ath00010	Glycolysis / Gluconeogenesis	gene_EYR40_006626/gene_EYR40_010880/gene_EYR40_010439/gene_EYR40_009513/gene_EYR40_001984/gene_EYR40_003404/gene_EYR40_008608/gene_EYR40_006780/gene_EYR40_002629/gene_EYR40_	EYR40_006626/EYR40_010880/EYR40_010439/PDC1/ALD5_1/EYR40_003404/PGM2/PCK1_1/EYR40_002629/FDH1_1/GLK1/EYR40_000613/PGI1/EYR40_010481/PYK1/PGK1/EYR40_009302/EYR40_005016	ath:AT4G36250/ath:AT2G37790/ath:AT1G79530/ath:AT5G01320/ath:AT1G23800/ath:AT1G74030/ath:AT5G51820/ath:AT4G37870/ath:AT4G17260/ath:AT5G43940/ath:AT2G19860/ath:AT3G08590/ath:AT5G42740	18	0		18	gene_EYR40_006626/gene_EYR40_010880/gene_EYR40_010439/gene_EYR40_009513/gene_EYR40_001984/gene_EYR40_003404/gene_EYR40_008608/gene_EYR40_006780/gene_EYR40_002629/gene_EYR40_

KEGGID	Description	geneID	geneName	keggID	Count	Up	Up_Gene_id	Down	Down_Gene_id
		005558/gene_EYR40_005681/gene_EYR40_000613/gene_EYR40_006397/gene_EYR40_010481/gene_EYR40_003896/gene_EYR40_001182/gene_EYR40_009302/gene_EYR40_005016		/ath:AT1G79530/ath:AT5G63680/ath:AT1G79550/ath:AT5G03690/ath:AT2G37790					005558/gene_EYR40_005681/gene_EYR40_000613/gene_EYR40_006397/gene_EYR40_010481/gene_EYR40_003896/gene_EYR40_001182/gene_EYR40_009302/gene_EYR40_005016
ath00030	Pentose phosphate pathway	gene_EYR40_005655/gene_EYR40_008608/gene_EYR40_001841/gene_EYR40_004207/gene_EYR40_006	ZWF1/PGM2/6PGD/SOL1/PGI1/TAL1_2/DOR14/TAL1_1/TKL1/EYR40_009302	ath:AT5G40760/ath:AT5G51820/ath:AT5G41670/ath:AT5G24400/ath:AT5G42740/ath:AT1G12230/ath:AT5	10	0		10	gene_EYR40_005655/gene_EYR40_008608/gene_EYR40_001841/gene_EYR40_004207/gene_EYR40_006

KEGGID	Description	geneID	geneName	keggID	Count	Up	Up_Gene_id	Down	Down_Gene_id
		397/gene_EYR40_009230/gene_EYR40_004536/gene_EYR40_001028/gene_EYR40_001125/gene_EYR40_009302		G41670/ath:AT1G12230/ath:AT2G45290/ath:AT5G03690					397/gene_EYR40_009230/gene_EYR40_004536/gene_EYR40_001028/gene_EYR40_001125/gene_EYR40_009302
ath01230	Biosynthesis of amino acids	gene_EYR40_010439/gene_EYR40_005141/gene_EYR40_003404/gene_EYR40_005259/gene_EYR40_000721/gene_EYR40_007985/gene_EYR40_000613/gene_EYR40_010481/	EYR40_010439/LEU2/EYR40_003404/MET6/EYR40_000721/EYR40_007985/EYR40_000613/EYR40_010481/PYK1/TAL1_2/TAL1_1/TKL1/EYR40_003938/EYR40_004318/ARG4/TR	ath:AT1G79530/ath:AT1G31180/ath:AT1G74030/ath:AT5G17920/ath:AT5G11520/ath:AT2G42790/ath:AT3G08590/ath:AT1G79530/ath:AT5G63680/ath:AT1G12230/ath:AT1G122	25	2	gene_EYR40_003130/gene_EYR40_003137	23	gene_EYR40_010439/gene_EYR40_005141/gene_EYR40_003404/gene_EYR40_005259/gene_EYR40_000721/gene_EYR40_007985/gene_EYR40_000613/gene_EYR40_010481/

KEGGID	Description	geneID	geneName	keggID	Count	Up	Up_Gene_id	Down	Down_Gene_id
		gene_EYR40_003896/gene_EYR40_009230/gene_EYR40_001028/gene_EYR40_001125/gene_EYR40_003938/gene_EYR40_004318/gene_EYR40_000718/gene_EYR40_000921/gene_EYR40_008814/gene_EYR40_001182/gene_EYR40_000701/gene_EYR40_005436/gene_EYR40_009302/gene_E	P5/ARG1/PGK1/PRO3/LEU1/EYR40_009302/EYR40_010752/HOM3_1/EYR40_002733/HOM3_2	30/ath:AT2G45290/ath:AT1G17745/ath:AT4G39980/ath:AT5G10920/ath:AT4G27070/ath:AT4G24830/ath:AT1G79550/ath:AT5G14800/ath:AT4G13430/ath:AT5G03690/ath:AT1G31230/ath:AT5G13280/ath:AT3G57050/ath:AT3G02020					gene_EYR40_003896/gene_EYR40_009230/gene_EYR40_001028/gene_EYR40_001125/gene_EYR40_003938/gene_EYR40_004318/gene_EYR40_000718/gene_EYR40_000921/gene_EYR40_008814/gene_EYR40_001182/gene_EYR40_000701/gene_EYR40_005436/gene_EYR40_009302/gene_E

KEGGID	Description	geneID	geneName	keggID	Count	Up	Up_Gene_id	Down	Down_Gene_id
		YR40_010752/gene_EYR40_003130/gene_EYR40_002733/gene_EYR40_003137							YR40_010752/gene_EYR40_002733
ath01110	Biosynthesis of secondary metabolites	gene_EYR40_006900/gene_EYR40_008023/gene_EYR40_006780/gene_EYR40_009514/gene_EYR40_007257/gene_EYR40_004142/gene_EYR40_010752/gene_EYR40_002971/gene_EYR40_002735/ge	EYR40_006900/EYR40_008023/PCK1_1/EYR40_009514/BTS1/EYR40_004142/EYR40_010752/HEM13/EYR40_002735/EYR40_000113/PDC1/EYR40_009367/TPS1/HIS5/TPS2/SAM2/EYR40_008586/HO	ath:AT3G53260/ath:AT1G74540/ath:AT4G37870/ath:AT1G74550/ath:AT1G78510/ath:AT3G16910/ath:AT1G31230/ath:AT1G03475/ath:AT1G24360/ath:AT3G12900/ath:AT5G01320/ath:AT3G48560/ath:	45	20	gene_EYR40_006900/gene_EYR40_008023/gene_EYR40_009514/gene_EYR40_004142/gene_EYR40_002735/gene_EYR40_000113/gene_EYR40_000905/gene_EYR40_008586/gene_EYR40_003130/ge	25	gene_EYR40_006780/gene_EYR40_007257/gene_EYR40_010752/gene_EYR40_002971/gene_EYR40_009513/gene_EYR40_009367/gene_EYR40_007797/gene_EYR40_002845/gene_EYR40_011013/ge

KEGGID	Description	geneID	geneName	keggID	Count	Up	Up_Gene_id	Down	Down_Gene_id
		ne_EYR40_000113/gene_EYR40_009513/gene_EYR40_009367/gene_EYR40_007797/gene_EYR40_002845/gene_EYR40_011013/gene_EYR40_000905/gene_EYR40_008586/gene_EYR40_003130/gene_EYR40_000112/gene_EYR40_000111/gene_EYR40_003449/gene_EYR40_004496/gene_EY	M3_1/EYR40_000112/ACS1/EYR40_003449/GUT2/PMI1/ERG7/EYR40_004481/ADH1_2/EYR40_000537/EYR40_000613/NDK1/EYR40_002629/EYR40_003404/EYR40_007776/EYR40_009573/TAL1_2/ERG1_1/CYB2_2/UGP1_2/ADE10/EYR40_009302/HOM3_2/EYR40_003963/TAL1_1/EYR40_0	AT1G78580/ath:AT1G71920/ath:AT1G78580/ath:AT2G36880/ath:AT4G11820/ath:AT5G13280/ath:AT3G12900/ath:AT5G36880/ath:AT4G13010/ath:AT3G10370/ath:AT3G02570/ath:AT2G07050/ath:AT5G06060/ath:AT4G37980/ath:AT1G55510/ath:AT3G08590/ath:AT4G09320/ath:AT4G17260/at			ne_EYR40_000112/gene_EYR40_000111/gene_EYR40_004496/gene_EYR40_009845/gene_EYR40_000537/gene_EYR40_009573/gene_EYR40_008395/gene_EYR40_007083/gene_EYR40_003137/gene_EYR40_003963/gene_EYR40_003799		ne_EYR40_003449/gene_EYR40_008405/gene_EYR40_004481/gene_EYR40_010824/gene_EYR40_000613/gene_EYR40_001191/gene_EYR40_002629/gene_EYR40_003404/gene_EYR40_007776/gene_EYR40_009230/gene_EYR40_006516/gene_EYR40_009051/gene_EYR40_009302/gene_EY

KEGGID	Description	geneID	geneName	keggID	Count	Up	Up_Gene_id	Down	Down_Gene_id
		R40_008405/gene_EYR40_009845/gene_EYR40_004481/gene_EYR40_010824/gene_EYR40_000537/gene_EYR40_000613/gene_EYR40_001191/gene_EYR40_002629/gene_EYR40_003404/gene_EYR40_007776/gene_EYR40_009573/gene_EYR40_009230/gene_EYR40_008395/gene_EYR40_00	03799/FBP1/EYR40_009740	h:AT1G74030/ath:AT1G76680/ath:AT1G21400/ath:AT1G12230/ath:AT1G58440/ath:AT3G14415/ath:AT3G03250/ath:AT2G35040/ath:AT5G03690/ath:AT3G02020/ath:AT2G29320/ath:AT1G12230/ath:AT4G05160/ath:AT1G43670/ath:AT5G08335					R40_001028/gene_EYR40_000810/gene_EYR40_009740

KEGGID	Description	geneID	geneName	keggID	Count	Up	Up_Gene_id	Down	Down_Gene_id
		7083/gene_EYR40_006516/gene_EYR40_009051/gene_EYR40_009302/gene_EYR40_003137/gene_EYR40_003963/gene_EYR40_001028/gene_EYR40_003799/gene_EYR40_000810/gene_EYR40_009740							
ath00010	Glycolysis / Gluconeogenesis	gene_EYR40_006780/gene_EYR40_004142/gene_EYR40_009513/gene_EYR40_00	PCK1_1/EYR40_004142/PDC1/ACS1/EYR40_000613/EYR40_002629/EYR40_003404	ath:AT4G37870/ath:AT3G16910/ath:AT5G01320/ath:AT5G36880/ath:AT3G08590/at	9	2	gene_EYR40_004142/gene_EYR40_000111	7	gene_EYR40_006780/gene_EYR40_009513/gene_EYR40_000613/gene_EYR40_00

KEGGID	Description	geneID	geneName	keggID	Count	Up	Up_Gene_id	Down	Down_Gene_id
		0111/gene_EYR40_000613/gene_EYR40_002629/gene_EYR40_003404/gene_EYR40_009302/gene_EYR40_000810	/EYR40_009302/FBP1	h:AT4G17260/ath:AT1G74030/ath:AT5G03690/ath:AT1G43670					2629/gene_EYR40_003404/gene_EYR40_009302/gene_EYR40_000810
**KEGG enrichment between B10 and CK samples**									
ath01110	Biosynthesis of secondary metabolites	gene_EYR40_006900/gene_EYR40_008023/gene_EYR40_006780/gene_EYR40_009514/gene_EYR40_007257/gene_EYR40_004142/gene_EYR40_010752/gene_EYR	EYR40_006900/EYR40_008023/PCK1_1/EYR40_009514/BTS1/EYR40_004142/EYR40_010752/HEM13/EYR40_002735/EYR40_000113/PDC1/EYR40_009367/TPS1/HIS	ath:AT3G53260/ath:AT1G74540/ath:AT4G37870/ath:AT1G74550/ath:AT1G78510/ath:AT3G16910/ath:AT1G31230/ath:AT1G03475/ath:AT1G24360/ath:AT3G12900/at	45	20	gene_EYR40_006900/gene_EYR40_008023/gene_EYR40_009514/gene_EYR40_004142/gene_EYR40_002735/gene_EYR40_000113/gene_EYR40_000905/gene_EYR	25	gene_EYR40_006780/gene_EYR40_007257/gene_EYR40_010752/gene_EYR40_002971/gene_EYR40_009513/gene_EYR40_009367/gene_EYR40_007797/gene_EYR

KEGGID	Description	geneID	geneName	keggID	Count	Up	Up_Gene_id	Down	Down_Gene_id
		40_002971/gene_EYR40_002735/gene_EYR40_000113/gene_EYR40_009513/gene_EYR40_009367/gene_EYR40_007797/gene_EYR40_002845/gene_EYR40_011013/gene_EYR40_000905/gene_EYR40_008586/gene_EYR40_003130/gene_EYR40_000112/gene_EYR40_000111/gene_EYR40_003	5/TPS2/SAM2/EYR40_008586/HOM3_1/EYR40_000112/ACS1/EYR40_003449/GUT2/PMI1/ERG7/EYR40_004481/ADH1_2/EYR40_000537/EYR40_000613/NDK1/EYR40_002629/EYR40_003404/EYR40_007776/EYR40_009573/TAL1_2/ERG1_1/CYB2_2/UGP1_2/ADE10/EYR40_009302/HOM3	h:AT5G01320/ath:AT3G48560/ath:AT1G78580/ath:AT1G71920/ath:AT1G78580/ath:AT2G36880/ath:AT4G11820/ath:AT5G13280/ath:AT3G12900/ath:AT5G36880/ath:AT4G13010/ath:AT3G10370/ath:AT3G02570/ath:AT2G07050/ath:AT5G06060/ath:AT4G37980/ath:AT1G55510/ath:AT3G08590			40_008586/gene_EYR40_003130/gene_EYR40_000112/gene_EYR40_000111/gene_EYR40_004496/gene_EYR40_009845/gene_EYR40_000537/gene_EYR40_009573/gene_EYR40_008395/gene_EYR40_007083/gene_EYR40_003137/gene_EYR40_003963/gene_EYR40_003799		40_002845/gene_EYR40_011013/gene_EYR40_003449/gene_EYR40_008405/gene_EYR40_004481/gene_EYR40_010824/gene_EYR40_000613/gene_EYR40_001191/gene_EYR40_002629/gene_EYR40_003404/gene_EYR40_007776/gene_EYR40_009230/gene_EYR40_006516/gene_EYR40_009

KEGGID	Description	geneID	geneName	keggID	Count	Up	Up_Gene_id	Down	Down_Gene_id
		449/gene_EYR40_004496/gene_EYR40_008405/gene_EYR40_009845/gene_EYR40_004481/gene_EYR40_010824/gene_EYR40_000537/gene_EYR40_000613/gene_EYR40_001191/gene_EYR40_002629/gene_EYR40_003404/gene_EYR40_007776/gene_EYR40_009573/gene_EYR40_009230/gen	_2/EYR40_003963/TAL1_1/EYR40_003799/FBP1/EYR40_009740	/ath:AT4G09320/ath:AT4G17260/ath:AT1G74030/ath:AT1G76680/ath:AT1G21400/ath:AT1G12230/ath:AT1G58440/ath:AT3G14415/ath:AT3G03250/ath:AT2G35040/ath:AT5G03690/ath:AT3G02020/ath:AT2G29320/ath:AT1G12230/ath:AT4G05160/ath:AT1G43670/ath:AT5G08335					051/gene_EYR40_009302/gene_EYR40_001028/gene_EYR40_000810/gene_EYR40_009740

KEGGID	Description	geneID	geneName	keggID	Count	Up	Up_Gene_id	Down	Down_Gene_id
		e_EYR40_008395/gene_EYR40_007083/gene_EYR40_006516/gene_EYR40_009051/gene_EYR40_009302/gene_EYR40_003137/gene_EYR40_003963/gene_EYR40_001028/gene_EYR40_003799/gene_EYR40_000810/gene_EYR40_009740							
ath00010	Glycolysis / Gluconeogenesis	gene_EYR40_006780/gene_EYR40_004142/gen	PCK1_1/EYR40_004142/PDC1/ACS1/EYR40_00	ath:AT4G37870/ath:AT3G16910/ath:AT5G01320	9	2	gene_EYR40_004142/gene_EYR40_000111	7	gene_EYR40_006780/gene_EYR40_009513/gen

KEGGID	Description	geneID	geneName	keggID	Count	Up	Up_Gene_id	Down	Down_Gene_id
		e_EYR40_009513/gene_EYR40_000111/gene_EYR40_000613/gene_EYR40_002629/gene_EYR40_003404/gene_EYR40_009302/gene_EYR40_000810	0613/EYR40_002629/EYR40_003404/EYR40_009302/FBP1	/ath:AT5G36880/ath:AT3G08590/ath:AT4G17260/ath:AT1G74030/ath:AT5G03690/ath:AT1G43670					e_EYR40_000613/gene_EYR40_002629/gene_EYR40_003404/gene_EYR40_009302/gene_EYR40_000810
ath01110	Biosynthesis of secondary metabolites	gene_EYR40_006900/gene_EYR40_008023/gene_EYR40_006780/gene_EYR40_009514/gene_EYR40_007257/gene_EYR40_004142/gene_EY	EYR40_006900/EYR40_008023/PCK1_1/EYR40_009514/BTS1/EYR40_004142/EYR40_010752/HEM13/EYR40_002735/EYR40_000113/PDC1/E	ath:AT3G53260/ath:AT1G74540/ath:AT4G37870/ath:AT1G74550/ath:AT1G78510/ath:AT3G16910/ath:AT1G31230/ath:AT1G03475/ath:AT1G2	45	20	gene_EYR40_006900/gene_EYR40_008023/gene_EYR40_009514/gene_EYR40_004142/gene_EYR40_002735/gene_EYR40_000113/gene_EY	25	gene_EYR40_006780/gene_EYR40_007257/gene_EYR40_010752/gene_EYR40_002971/gene_EYR40_009513/gene_EYR40_009367/gene_EY

KEGGID	Description	geneID	geneName	keggID	Count	Up	Up_Gene_id	Down	Down_Gene_id
		R40_010752/gene_EYR40_002971/gene_EYR40_002735/gene_EYR40_000113/gene_EYR40_009513/gene_EYR40_009367/gene_EYR40_007797/gene_EYR40_002845/gene_EYR40_011013/gene_EYR40_000905/gene_EYR40_008586/gene_EYR40_003130/gene_EYR40_000112/gene_EYR40_00	YR40_009367/TPS1/HIS5/TPS2/SAM2/EYR40_008586/HOM3_1/EYR40_000112/ACS1/EYR40_003449/GUT2/PMI1/ERG7/EYR40_004481/ADH1_2/EYR40_000537/EYR40_000613/NDK1/EYR40_002629/EYR40_003404/EYR40_007776/EYR40_009573/TAL1_2/ERG1_1/CYB2_2/UGP1_2/ADE10/	4360/ath:AT3G12900/ath:AT5G01320/ath:AT3G48560/ath:AT1G78580/ath:AT1G71920/ath:AT1G78580/ath:AT2G36880/ath:AT4G11820/ath:AT5G13280/ath:AT3G12900/ath:AT5G36880/ath:AT4G13010/ath:AT3G10370/ath:AT3G02570/ath:AT2G07050/ath:AT5G06060/ath:AT4G37980/ath:AT1			R40_000905/gene_EYR40_008586/gene_EYR40_003130/gene_EYR40_000112/gene_EYR40_000111/gene_EYR40_004496/gene_EYR40_009845/gene_EYR40_000537/gene_EYR40_009573/gene_EYR40_008395/gene_EYR40_007083/gene_EYR40_003137/gene_EYR40_003963/gene_EYR40_00		R40_007797/gene_EYR40_002845/gene_EYR40_011013/gene_EYR40_003449/gene_EYR40_008405/gene_EYR40_004481/gene_EYR40_010824/gene_EYR40_000613/gene_EYR40_001191/gene_EYR40_002629/gene_EYR40_003404/gene_EYR40_007776/gene_EYR40_009230/gene_EYR40_00

KEGGID	Description	geneID	geneName	keggID	Count	Up	Up_Gene_id	Down	Down_Gene_id
		0111/gene_EYR40_003449/gene_EYR40_004496/gene_EYR40_008405/gene_EYR40_009845/gene_EYR40_004481/gene_EYR40_010824/gene_EYR40_000537/gene_EYR40_000613/gene_EYR40_001191/gene_EYR40_002629/gene_EYR40_003404/gene_EYR40_007776/gene_EYR40_009573/ge	EYR40_009302/HOM3_2/EYR40_003963/TAL1_1/EYR40_003799/FBP1/EYR40_009740	G55510/ath:AT3G08590/ath:AT4G09320/ath:AT4G17260/ath:AT1G74030/ath:AT1G76680/ath:AT1G21400/ath:AT1G12230/ath:AT1G58440/ath:AT3G14415/ath:AT3G03250/ath:AT2G35040/ath:AT5G03690/ath:AT3G02020/ath:AT2G29320/ath:AT1G12230/ath:AT4G05160/ath:AT1G43670/ath:AT			3799		6516/gene_EYR40_009051/gene_EYR40_009302/gene_EYR40_001028/gene_EYR40_000810/gene_EYR40_009740

KEGGID	Description	geneID	geneName	keggID	Count	Up	Up_Gene_id	Down	Down_Gene_id
		ne_EYR40_009230/gene_EYR40_008395/gene_EYR40_007083/gene_EYR40_006516/gene_EYR40_009051/gene_EYR40_009302/gene_EYR40_003137/gene_EYR40_003963/gene_EYR40_001028/gene_EYR40_003799/gene_EYR40_000810/gene_EYR40_009740		5G08335					

KEGGID: KEGG pathway number,Description: Functional description corresponding to KEGG pathway number,geneID: Differential gene ID annotated to KEGG pathway number,geneName: Differential gene name annotated to KEGG pathway number,keggID: Differential gene keggID

annotated to KEGG pathway number. Count: number of differential genes annotated to KEGG pathway number,Up: number of up-regulated differential genes associated with this Term,Up_Gene_id: ID of up-regulated differential genes associated with this Term,Down: number of down-regulated differential genes associated with this Term,Down_Gene_id: ID of down-regulated differential genes associated with this Term. Down_Gene_id: down-regulated differential gene id associated with the Term

Table S3. List of Significantly Enriched Results for Differential Gene GO

Category	GOID	Description	geneID	geneName	Count	Up	Up_Gene_id	Down	Down_Gene_id
**GO enrichment between A5 and B5 samples**									
BP	GO:0005975	carbohydrate metabolic process	gene_EYR40_008178/gene_EYR40_004178/gene_EYR40_001127/gene_EYR40_007113/gene_EYR40_009219/gene_EYR40_005636/gene_EYR40_004599/gene_EYR40_009076/gene_EYR40_005655/gene_EYR40_006825/gene_EYR40_002023/gene_EYR40_008	D25_4/EYR40_004178/EYR40_001127/EYR40_007113/NAG2/EYR40_005636/EYR40_004599/CBH1_11/ZWF1/EYR40_006825/EYR40_002023/CBH1_10/INO1/EYR40_011040/P	45	27	gene_EYR40_008178/gene_EYR40_007113/gene_EYR40_009219/gene_EYR40_005636/gene_EYR40_004599/gene_EYR40_005655/gene_EYR40_006825/gene_EYR40_002023/	18	gene_EYR40_004178/gene_EYR40_001127/gene_EYR40_009076/gene_EYR40_008545/gene_EYR40_011040/gene_EYR40_005104/gene_EYR40_009079/gene_EYR40_002335/ge

Category	GOID	Description	geneID	geneName	Count	Up	Up_Gene_id	Down	Down_Gene_id
			545/gene_EYR40_007279/gene_EYR40_011040/gene_EYR40_003896/gene_EYR40_005104/gene_EYR40_002850/gene_EYR40_004072/gene_EYR40_009344/gene_EYR40_009079/gene_EYR40_002335/gene_EYR40_009098/gene_EYR40_009966/gene_EYR40_000019/gene_EYR40_001194/gene_EYR40_000965/gene_EYR40_010283/gene_EYR40_005657/gene_EYR40_003898/gene_EYR40_003253/gene_EYR40_008698/gene_EY	YK1/EYR40_005104/EYR40_002850/EYR40_004072/EYR40_009344/EYR40_009079/XYL5_2/GUT1/EYR40_009966/EYR40_000019/PGM3/EYR40_000965/EYR40_010283/CBH1_5/RPE1/EYR40_003253/EYR40_008698/EXG3/CEL1_8/EYR40_009768/EYR40_009556/EYR40_006395/EYR40_010195/			gene_EYR40_007279/gene_EYR40_003896/gene_EYR40_002850/gene_EYR40_004072/gene_EYR40_009344/gene_EYR40_009098/gene_EYR40_009966/gene_EYR40_000019/gene_EYR40_001194/gene_EYR40_003253/gene_EYR40_008698/gene_EYR40_000714/gene_EYR40_009945/gene_E		ne_EYR40_000965/gene_EYR40_010283/gene_EYR40_005657/gene_EYR40_003898/gene_EYR40_009768/gene_EYR40_006395/gene_EYR40_010195/gene_EYR40_009108/gene_EYR40_006750/gene_EYR40_004793

Category	GOID	Description	geneID	geneName	Count	Up	Up_Gene_id	Down	Down_Gene_id
			R40_000714/gene_EYR40_009945/gene_EYR40_009768/gene_EYR40_009556/gene_EYR40_006395/gene_EYR40_010195/gene_EYR40_002317/gene_EYR40_004279/gene_EYR40_009108/gene_EYR40_006750/gene_EYR40_003410/gene_EYR40_002651/gene_EYR40_004793/gene_EYR40_009086	XYL5_1/CHT1_2/D25_5/EYR40_006750/EYR40_003410/EYR40_002651/EYR40_004793/CBH1_13			YR40_009556/gene_EYR40_002317/gene_EYR40_004279/gene_EYR40_003410/gene_EYR40_002651/gene_EYR40_009086		
CC	GO:0016021	integral component of membrane	gene_EYR40_010044/gene_EYR40_008395/gene_EYR40_009863/gene_EYR40_009862/gene_EYR40_008326/gene_EYR	EYR40_010044/ERG1_1/EYR40_009863/EYR40_009862/EYR40_008326/EYR40_010	51	19	gene_EYR40_008395/gene_EYR40_006760/gene_EYR40_002030/gene_EYR40_00	32	gene_EYR40_010044/gene_EYR40_009863/gene_EYR40_009862/gene_EYR40_00

Category	GOID	Description	geneID	geneName	Count	Up	Up_Gene_id	Down	Down_Gene_id
			40_010842/gene_EYR40_005038/gene_EYR40_006760/gene_EYR40_002030/gene_EYR40_005479/gene_EYR40_008424/gene_EYR40_009134/gene_EYR40_003471/gene_EYR40_005453/gene_EYR40_010935/gene_EYR40_001006/gene_EYR40_007431/gene_EYR40_003681/gene_EYR40_003020/gene_EYR40_000469/gene_EYR40_007818/gene_EYR40_007606/gene_EYR40_007944/gene_EYR40_001432/gene_EYR40_003248/ge	842/EYR40_005038/EYR40_006760/EYR40_002030/EYR40_005479/EYR40_008424/EYR40_009134/EYR40_003471/EYR40_005453/SEC62_2/EYR40_001006/EYR40_007431/EYR40_003681/EYR40_003020/EYR40_000469/PHO84_2/EYR40_007606/EYR40_007944/EYR40_001432/EYR40_003248/EYR40			9134/gene_EYR40_003471/gene_EYR40_010935/gene_EYR40_001006/gene_EYR40_007431/gene_EYR40_007606/gene_EYR40_004839/gene_EYR40_006693/gene_EYR40_002411/gene_EYR40_003560/gene_EYR40_006647/gene_EYR40_011133/gene_EYR40_008568/gene_EYR40_005087/ge		8326/gene_EYR40_010842/gene_EYR40_005038/gene_EYR40_005479/gene_EYR40_008424/gene_EYR40_005453/gene_EYR40_003681/gene_EYR40_003020/gene_EYR40_000469/gene_EYR40_007818/gene_EYR40_007944/gene_EYR40_001432/gene_EYR40_003248/gene_EYR40_004303/gene

Category	GOID	Description	geneID	geneName	Count	Up	Up_Gene_id	Down	Down_Gene_id
			ne_EYR40_004839/gene_EYR40_004303/gene_EYR40_010360/gene_EYR40_006360/gene_EYR40_009912/gene_EYR40_010155/gene_EYR40_006133/gene_EYR40_006693/gene_EYR40_003609/gene_EYR40_008126/gene_EYR40_002411/gene_EYR40_003560/gene_EYR40_004880/gene_EYR40_008523/gene_EYR40_008786/gene_EYR40_006361/gene_EYR40_006647/gene_EYR40_002267/gene_EYR40_011133/gene_EYR40_009	_004839/EYR40_004303/EYR40_010360/EYR40_006360/EYR40_009912/EYR40_010155/MDR1_1/STE3_2/EYR40_003609/EYR40_008126/EYR40_002411/EYR40_003560/EYR40_004880/EYR40_008523/EYR40_008786/EYR40_006361/EYR40_006647/HXT1_1/EYR40_011133/EYR40_009740/EYR40_00			ne_EYR40_003559/gene_EYR40_008240		_EYR40_010360/gene_EYR40_006360/gene_EYR40_009912/gene_EYR40_010155/gene_EYR40_006133/gene_EYR40_003609/gene_EYR40_008126/gene_EYR40_004880/gene_EYR40_008523/gene_EYR40_008786/gene_EYR40_006361/gene_EYR40_002267/gene_EYR40_009740/gene_EYR40_0

Category	GOID	Description	geneID	geneName	Count	Up	Up_Gene_id	Down	Down_Gene_id
			740/gene_EYR40_008581/gene_EYR40_008568/gene_EYR40_008009/gene_EYR40_005087/gene_EYR40_003559/gene_EYR40_008240	8581/EYR40_008568/EYR40_008009/EYR40_005087/EYR40_003559/EYR40_008240					08581/gene_EYR40_008009
CC	GO:0031224	intrinsic component of membrane	gene_EYR40_010044/gene_EYR40_008395/gene_EYR40_009863/gene_EYR40_009862/gene_EYR40_008326/gene_EYR40_010842/gene_EYR40_005038/gene_EYR40_006760/gene_EYR40_002030/gene_EYR40_005479/gene_EYR40_008424/gene_EYR40_009134/gene_EYR40_003471/gene_E	EYR40_010044/ERG1_1/EYR40_009863/EYR40_009862/EYR40_008326/EYR40_010842/EYR40_005038/EYR40_006760/EYR40_002030/EYR40_005479/EYR40_008424/EYR40_009134/EYR40_003471/EYR	51	19	gene_EYR40_008395/gene_EYR40_006760/gene_EYR40_002030/gene_EYR40_009134/gene_EYR40_003471/gene_EYR40_010935/gene_EYR40_001006/gene_EYR40_007431/gene_EYR40_007606/ge	32	gene_EYR40_010044/gene_EYR40_009863/gene_EYR40_009862/gene_EYR40_008326/gene_EYR40_010842/gene_EYR40_005038/gene_EYR40_005479/gene_EYR40_008424/gene_EYR40_005453/gen

Category	GOID	Description	geneID	geneName	Count	Up	Up_Gene_id	Down	Down_Gene_id
			YR40_005453/gene_EYR40_010935/gene_EYR40_001006/gene_EYR40_007431/gene_EYR40_003681/gene_EYR40_003020/gene_EYR40_000469/gene_EYR40_007818/gene_EYR40_007606/gene_EYR40_007944/gene_EYR40_001432/gene_EYR40_003248/gene_EYR40_004839/gene_EYR40_004303/gene_EYR40_010360/gene_EYR40_006360/gene_EYR40_009912/gene_EYR40_010155/gene_EYR40_006133/gene_EYR40_006693/	40_005453/SEC62_2/EYR40_001006/EYR40_007431/EYR40_003681/EYR40_003020/EYR40_000469/PHO84_2/EYR40_007606/EYR40_007944/EYR40_001432/EYR40_003248/EYR40_004839/EYR40_004303/EYR40_010360/EYR40_006360/EYR40_009912/EYR40_010155/MDR1_1/STE3_2/EYR40_00360			ne_EYR40_004839/gene_EYR40_006693/gene_EYR40_002411/gene_EYR40_003560/gene_EYR40_006647/gene_EYR40_011133/gene_EYR40_008568/gene_EYR40_005087/gene_EYR40_003559/gene_EYR40_008240		e_EYR40_003681/gene_EYR40_003020/gene_EYR40_000469/gene_EYR40_007818/gene_EYR40_007944/gene_EYR40_001432/gene_EYR40_003248/gene_EYR40_004303/gene_EYR40_010360/gene_EYR40_006360/gene_EYR40_009912/gene_EYR40_010155/gene_EYR40_006133/gene_EYR40_

Category	GOID	Description	geneID	geneName	Count	Up	Up_Gene_id	Down	Down_Gene_id
			gene_EYR40_003609/gene_EYR40_008126/gene_EYR40_002411/gene_EYR40_003560/gene_EYR40_004880/gene_EYR40_008523/gene_EYR40_008786/gene_EYR40_006361/gene_EYR40_006647/gene_EYR40_002267/gene_EYR40_011133/gene_EYR40_009740/gene_EYR40_008581/gene_EYR40_008568/gene_EYR40_008009/gene_EYR40_005087/gene_EYR40_003559/gene_EYR40_008240	9/EYR40_008126/EYR40_002411/EYR40_003560/EYR40_004880/EYR40_008523/EYR40_008786/EYR40_006361/EYR40_006647/HXT1_1/EYR40_011133/EYR40_009740/EYR40_008581/EYR40_008568/EYR40_008009/EYR40_005087/EYR40_003559/EYR40_008240					003609/gene_EYR40_008126/gene_EYR40_004880/gene_EYR40_008523/gene_EYR40_008786/gene_EYR40_006361/gene_EYR40_002267/gene_EYR40_009740/gene_EYR40_008581/gene_EYR40_008009
CC	GO:0005576	extracellular	gene_EYR40_004178/gene_EYR40	EYR40_004178/CBH1_	13	5	gene_EYR40_004760/ge	8	gene_EYR40_004178/ge

Category	GOID	Description	geneID	geneName	Count	Up	Up_Gene_id	Down	Down_Gene_id
		region	_009076/gene_EYR40_009079/gene_EYR40_005657/gene_EYR40_004760/gene_EYR40_009945/gene_EYR40_009768/gene_EYR40_006395/gene_EYR40_004279/novel.51/gene_EYR40_006750/gene_EYR40_003410/gene_EYR40_009086	11/EYR40_009079/CBH1_5/EYR40_004760/CEL1_8/EYR40_009768/EYR40_006395/CHT1_2/-/EYR40_006750/EYR40_003410/CBH1_13			ne_EYR40_009945/gene_EYR40_004279/gene_EYR40_003410/gene_EYR40_009086		ne_EYR40_009076/gene_EYR40_009079/gene_EYR40_005657/gene_EYR40_009768/gene_EYR40_006395/novel.51/gene_EYR40_006750
CC	GO:0044425	membrane part	gene_EYR40_010044/gene_EYR40_008395/gene_EYR40_009863/gene_EYR40_009862/gene_EYR40_008326/gene_EYR40_010842/gene_EYR40_005038/gene_EYR40_006760/gene_EYR40_	EYR40_010044/ERG1_1/EYR40_009863/EYR40_009862/EYR40_008326/EYR40_010842/EYR40_005038/EYR40_006760/EYR40_002	52	20	gene_EYR40_008395/gene_EYR40_006760/gene_EYR40_002030/gene_EYR40_009134/gene_EYR40_003471/gene_EYR40_01093	32	gene_EYR40_010044/gene_EYR40_009863/gene_EYR40_009862/gene_EYR40_008326/gene_EYR40_010842/gene_EYR40_00503

Category	GOID	Description	geneID	geneName	Count	Up	Up_Gene_id	Down	Down_Gene_id
			002030/gene_EYR40_005479/gene_EYR40_008424/gene_EYR40_009134/gene_EYR40_003471/gene_EYR40_005453/gene_EYR40_010935/gene_EYR40_001006/gene_EYR40_007431/gene_EYR40_003681/gene_EYR40_003020/gene_EYR40_000469/gene_EYR40_007818/gene_EYR40_007606/gene_EYR40_007944/gene_EYR40_001432/gene_EYR40_003248/gene_EYR40_004839/gene_EYR40_004303/gene_EYR40_010360/gene_	030/EYR40_005479/EYR40_008424/EYR40_009134/EYR40_003471/EYR40_005453/SEC62_2/EYR40_001006/EYR40_007431/EYR40_003681/EYR40_003020/EYR40_000469/PHO84_2/EYR40_007606/EYR40_007944/EYR40_001432/EYR40_003248/EYR40_004839/EYR40_004303/EYR40_010360/EYR40_			5/gene_EYR40_001006/gene_EYR40_007431/gene_EYR40_007606/gene_EYR40_004839/gene_EYR40_006693/gene_EYR40_002411/gene_EYR40_007793/gene_EYR40_003560/gene_EYR40_006647/gene_EYR40_011133/gene_EYR40_008568/gene_EYR40_005087/gene_EYR40_003559/gene		8/gene_EYR40_005479/gene_EYR40_008424/gene_EYR40_005453/gene_EYR40_003681/gene_EYR40_003020/gene_EYR40_000469/gene_EYR40_007818/gene_EYR40_007944/gene_EYR40_001432/gene_EYR40_003248/gene_EYR40_004303/gene_EYR40_010360/gene_EYR40_006360/gene_E

Category	GOID	Description	geneID	geneName	Count	Up	Up_Gene_id	Down	Down_Gene_id
			EYR40_006360/gene_EYR40_009912/gene_EYR40_010155/gene_EYR40_006133/gene_EYR40_006693/gene_EYR40_003609/gene_EYR40_008126/gene_EYR40_002411/gene_EYR40_007793/gene_EYR40_003560/gene_EYR40_004880/gene_EYR40_008523/gene_EYR40_008786/gene_EYR40_006361/gene_EYR40_006647/gene_EYR40_002267/gene_EYR40_011133/gene_EYR40_009740/gene_EYR40_008581/gene_EYR40_00856	006360/EYR40_009912/EYR40_010155/MDR1_1/STE3_2/EYR40_003609/EYR40_008126/EYR40_002411/VPS28/EYR40_003560/EYR40_004880/EYR40_008523/EYR40_008786/EYR40_006361/EYR40_006647/HXT1_1/EYR40_011133/EYR40_009740/EYR40_008581/EYR40_008568/EYR40_008009/EYR			_EYR40_008240		YR40_009912/gene_EYR40_010155/gene_EYR40_006133/gene_EYR40_003609/gene_EYR40_008126/gene_EYR40_004880/gene_EYR40_008523/gene_EYR40_008786/gene_EYR40_006361/gene_EYR40_002267/gene_EYR40_009740/gene_EYR40_008581/gene_EYR40_008009

Category	GOID	Description	geneID	geneName	Count	Up	Up_Gene_id	Down	Down_Gene_id
			8/gene_EYR40_008009/gene_EYR40_005087/gene_EYR40_003559/gene_EYR40_008240	40_005087/EYR40_003559/EYR40_008240					
MF	GO:0048037	cofactor binding	gene_EYR40_000292/gene_EYR40_008395/gene_EYR40_009103/gene_EYR40_006266/gene_EYR40_004362/gene_EYR40_003345/gene_EYR40_005082/gene_EYR40_008227/gene_EYR40_006810/gene_EYR40_000516/gene_EYR40_002937/gene_EYR40_007767/gene_EYR40_004262/gene_EYR40_002871/gene_EYR40_00800	EYR40_000292/ERG1_1/EYR40_009103/EYR40_006266/ERG5/EYR40_003345/EYR40_005082/EYR40_008227/EYR40_006810/LCB2/EYR40_002937/EYR40_007767/EYR40_004262/HMG1/ERG11/EYR40_007209/EYR40_004116	84	41	gene_EYR40_008395/gene_EYR40_004362/gene_EYR40_003345/gene_EYR40_008227/gene_EYR40_006810/gene_EYR40_000516/gene_EYR40_007767/gene_EYR40_002871/gene_EYR40_008002/gene_EYR40_007209/gen	43	gene_EYR40_000292/gene_EYR40_009103/gene_EYR40_006266/gene_EYR40_005082/gene_EYR40_002937/gene_EYR40_004262/novel.193/gene_EYR40_009367/gene_EYR40_005602/gene_EYR40_006607/gene_EYR40_00

Category	GOID	Description	geneID	geneName	Count	Up	Up_Gene_id	Down	Down_Gene_id
			2/gene_EYR40_007209/gene_EYR40_004116/gene_EYR40_007898/gene_EYR40_005655/novel.193/gene_EYR40_009367/gene_EYR40_003938/gene_EYR40_005602/gene_EYR40_006607/gene_EYR40_010868/gene_EYR40_008889/gene_EYR40_009812/gene_EYR40_004548/gene_EYR40_001709/gene_EYR40_000266/gene_EYR40_002845/novel.671/gene_EYR40_005784/gene_EYR40_011048/gene_EYR40_007800/gene_EYR40_0	/ACL1/ZWF1/-/EYR40_009367/EYR40_003938/EYR40_005602/EYR40_006607/EYR40_010868/EYR40_008889/EYR40_009812/EYR40_004548/EYR40_001709/EYR40_000266/HIS5/-/EYR40_005784/EYR40_011048/DIT2_3/EYR40_007794/EYR40_001983/EYR40_006278/-/EYR40_006			e_EYR40_004116/gene_EYR40_007898/gene_EYR40_005655/gene_EYR40_003938/gene_EYR40_010868/gene_EYR40_008889/gene_EYR40_009812/gene_EYR40_007800/gene_EYR40_001983/gene_EYR40_000519/gene_EYR40_008770/gene_EYR40_000871/gene_EYR40_008971/gene_EYR		4548/gene_EYR40_001709/gene_EYR40_000266/gene_EYR40_002845/novel.671/gene_EYR40_005784/gene_EYR40_011048/gene_EYR40_007794/gene_EYR40_006278/novel.311/gene_EYR40_006606/gene_EYR40_001477/gene_EYR40_002772/gene_EYR40_002483/gene_EYR40_006307/gene

Category	GOID	Description	geneID	geneName	Count	Up	Up_Gene_id	Down	Down_Gene_id
			07794/gene_EYR40_001983/gene_EYR40_006278/novel.311/gene_EYR40_006606/gene_EYR40_001477/gene_EYR40_002772/gene_EYR40_002483/gene_EYR40_000519/gene_EYR40_006307/gene_EYR40_010542/gene_EYR40_000298/gene_EYR40_009683/gene_EYR40_007536/gene_EYR40_008770/novel.80/gene_EYR40_006678/gene_EYR40_000871/gene_EYR40_008971/gene_EYR40_001458/gene_EYR40_003261/gene_EYR	606/EYR40_001477/EYR40_002772/EYR40_002483/UGD1/EYR40_006307/EYR40_010542/EYR40_000298/EYR40_009683/EYR40_007536/EYR40_008770/-/EYR40_006678/EYR40_000871/EYR40_008971/EYR40_001458/EYR40_003261/EYR40_004873/EYR40_002791/EYR40_003361/EYR40_004361/			40_004873/gene_EYR40_003361/gene_EYR40_004361/gene_EYR40_002527/gene_EYR40_007106/gene_EYR40_000308/gene_EYR40_002241/gene_EYR40_009394/gene_EYR40_009837/gene_EYR40_001704/gene_EYR40_002931/gene_EYR40_001793/gene_EYR40_001622/gene_EYR40_009		_EYR40_010542/gene_EYR40_000298/gene_EYR40_009683/gene_EYR40_007536/novel.80/gene_EYR40_006678/gene_EYR40_001458/gene_EYR40_003261/gene_EYR40_002791/gene_EYR40_010740/gene_EYR40_004699/gene_EYR40_002794/gene_EYR40_006394/gene_EYR40_003270/gene

Category	GOID	Description	geneID	geneName	Count	Up	Up_Gene_id	Down	Down_Gene_id
			40_004873/gene_EYR40_002791/gene_EYR40_003361/gene_EYR40_004361/gene_EYR40_010740/gene_EYR40_002527/gene_EYR40_004699/gene_EYR40_007106/gene_EYR40_000308/gene_EYR40_002794/gene_EYR40_002241/gene_EYR40_009394/gene_EYR40_009837/gene_EYR40_001704/gene_EYR40_006394/gene_EYR40_003270/gene_EYR40_006414/gene_EYR40_008385/gene_EYR40_002931/gene_EYR40_001793/ge	VPL2_3/EYR40_002527/GRE2_2/EYR40_007106/EYR40_000308/EYR40_002794/EYR40_002241/HST2/EYR40_009837/EYR40_001704/EYR40_006394/EYR40_003270/EYR40_006414/EYR40_008385/EYR40_002931/EYR40_001793/EYR40_001622/EYR40_009813/EYR40_002917/ADH1_2/EYR40_00			813/gene_EYR40_010824/gene_EYR40_000255/gene_EYR40_001133/gene_EYR40_004535		_EYR40_006414/gene_EYR40_008385/gene_EYR40_002917/gene_EYR40_001487

Category	GOID	Description	geneID	geneName	Count	Up	Up_Gene_id	Down	Down_Gene_id
			ne_EYR40_001622/gene_EYR40_009813/gene_EYR40_002917/gene_EYR40_010824/gene_EYR40_000255/gene_EYR40_001133/gene_EYR40_004535/gene_EYR40_001487	0255/EYR40_001133/EYR40_004535/EYR40_001487					
MF	GO:0005506	iron ion binding	gene_EYR40_003611/gene_EYR40_009103/gene_EYR40_006266/gene_EYR40_004362/gene_EYR40_008227/gene_EYR40_004262/gene_EYR40_008002/gene_EYR40_007209/gene_EYR40_007640/gene_EYR40_002425/novel.193/gene_EYR40_011040/gene_E	EYR40_003611/EYR40_009103/EYR40_006266/ERG5/EYR40_008227/EYR40_004262/ERG11/EYR40_007209/ERG3/ERG25/-/EYR40_011040/EYR40_010868/EYR40_009812/	39	19	gene_EYR40_003611/gene_EYR40_004362/gene_EYR40_008227/gene_EYR40_008002/gene_EYR40_007209/gene_EYR40_007640/gene_EYR40_002425/gene_EYR40_010868/	20	gene_EYR40_009103/gene_EYR40_006266/gene_EYR40_004262/novel.193/gene_EYR40_011040/novel.311/gene_EYR40_001477/gene_EYR40_002483/gene_EYR40_000298/gene

Category	GOID	Description	geneID	geneName	Count	Up	Up_Gene_id	Down	Down_Gene_id
			YR40_010868/gene_EYR40_009812/gene_EYR40_007800/gene_EYR40_001983/novel.311/gene_EYR40_001477/gene_EYR40_002483/gene_EYR40_000298/gene_EYR40_003202/novel.80/gene_EYR40_006678/gene_EYR40_001458/gene_EYR40_003261/gene_EYR40_002791/gene_EYR40_004361/gene_EYR40_007106/gene_EYR40_000308/gene_EYR40_002794/gene_EYR40_001926/gene_EYR40_002241/gene_EYR40_003270/g	DIT2_3/EYR40_001983/-/EYR40_001477/EYR40_002483/EYR40_000298/EYR40_003202/-/EYR40_006678/EYR40_001458/EYR40_003261/EYR40_002791/EYR40_004361/EYR40_007106/EYR40_000308/EYR40_002794/EYR40_001926/EYR40_002241/EYR40_003270/EYR40_006414/			gene_EYR40_009812/gene_EYR40_007800/gene_EYR40_001983/gene_EYR40_004361/gene_EYR40_007106/gene_EYR40_000308/gene_EYR40_002241/gene_EYR40_001793/gene_EYR40_009813/gene_EYR40_010824/gene_EYR40_000255		_EYR40_003202/novel.80/gene_EYR40_006678/gene_EYR40_001458/gene_EYR40_003261/gene_EYR40_002791/gene_EYR40_002794/gene_EYR40_001926/gene_EYR40_003270/gene_EYR40_006414/gene_EYR40_002917

Category	GOID	Description	geneID	geneName	Count	Up	Up_Gene_id	Down	Down_Gene_id
			ene_EYR40_006414/gene_EYR40_001793/gene_EYR40_009813/gene_EYR40_002917/gene_EYR40_010824/gene_EYR40_000255	EYR40_001793/EYR40_009813/EYR40_002917/ADH1_2/EYR40_000255					
MF	GO:0004553	hydrolase activity, hydrolyzing O-glycosyl compounds	gene_EYR40_001127/gene_EYR40_007113/gene_EYR40_009219/gene_EYR40_004599/gene_EYR40_009076/gene_EYR40_002023/gene_EYR40_008545/gene_EYR40_005104/gene_EYR40_004072/gene_EYR40_009344/gene_EYR40_010850/gene_EYR40_009079/gene_EYR40_002335/gene_E	EYR40_001127/EYR40_007113/NAG2/EYR40_004599/CBH1_11/EYR40_002023/CBH1_10/EYR40_005104/EYR40_004072/EYR40_009344/EYR40_010850/EYR40_009079/XYL5_2/EYR40_009966/EYR40_	31	16	gene_EYR40_007113/gene_EYR40_009219/gene_EYR40_004599/gene_EYR40_002023/gene_EYR40_004072/gene_EYR40_009344/gene_EYR40_009966/gene_EYR40_003253/gene_EYR40_008698/ge	15	gene_EYR40_001127/gene_EYR40_009076/gene_EYR40_008545/gene_EYR40_005104/gene_EYR40_010850/gene_EYR40_009079/gene_EYR40_002335/gene_EYR40_000965/gene_EYR40_005657/gen

Category	GOID	Description	geneID	geneName	Count	Up	Up_Gene_id	Down	Down_Gene_id
			YR40_009966/gene_EYR40_000965/gene_EYR40_005657/gene_EYR40_003253/gene_EYR40_008698/gene_EYR40_000714/gene_EYR40_007193/gene_EYR40_006395/gene_EYR40_010195/gene_EYR40_011010/gene_EYR40_002317/gene_EYR40_004279/gene_EYR40_010671/gene_EYR40_006750/gene_EYR40_003410/gene_EYR40_002651/gene_EYR40_004793/gene_EYR40_009086	000965/CBH1_5/EYR40_003253/EYR40_008698/EXG3/EYR40_007193/EYR40_006395/EYR40_010195/MNS1B_2/XYL5_1/CHT1_2/EYR40_010671/EYR40_006750/EYR40_003410/EYR40_002651/EYR40_004793/CBH1_13			ne_EYR40_000714/gene_EYR40_011010/gene_EYR40_002317/gene_EYR40_004279/gene_EYR40_003410/gene_EYR40_002651/gene_EYR40_009086		e_EYR40_007193/gene_EYR40_006395/gene_EYR40_010195/gene_EYR40_010671/gene_EYR40_006750/gene_EYR40_004793
MF	GO:0016798	hydrolase	gene_EYR40_001127/gene_EYR40	EYR40_001127/EYR40_	31	16	gene_EYR40_007113/ge	15	gene_EYR40_001127/ge

Category	GOID	Description	geneID	geneName	Count	Up	Up_Gene_id	Down	Down_Gene_id
		activity, acting on glycosyl bonds	_007113/gene_EYR40_009219/gene_EYR40_004599/gene_EYR40_009076/gene_EYR40_002023/gene_EYR40_008545/gene_EYR40_005104/gene_EYR40_004072/gene_EYR40_009344/gene_EYR40_010850/gene_EYR40_009079/gene_EYR40_002335/gene_EYR40_009966/gene_EYR40_000965/gene_EYR40_005657/gene_EYR40_003253/gene_EYR40_008698/gene_EYR40_000714/gene_EYR40_007193/gene_EYR40_006395/gene	007113/NAG2/EYR40_004599/CBH1_11/EYR40_002023/CBH1_10/EYR40_005104/EYR40_004072/EYR40_009344/EYR40_010850/EYR40_009079/XYL5_2/EYR40_009966/EYR40_000965/CBH1_5/EYR40_003253/EYR40_008698/EXG3/EYR40_007193/EYR40_006395/EYR40_010195/MNS1B_2/XYL			ne_EYR40_009219/gene_EYR40_004599/gene_EYR40_002023/gene_EYR40_004072/gene_EYR40_009344/gene_EYR40_009966/gene_EYR40_003253/gene_EYR40_008698/gene_EYR40_000714/gene_EYR40_011010/gene_EYR40_002317/gene_EYR40_004279/gene_EYR40_003410/gene_EY		ne_EYR40_009076/gene_EYR40_008545/gene_EYR40_005104/gene_EYR40_010850/gene_EYR40_009079/gene_EYR40_002335/gene_EYR40_000965/gene_EYR40_005657/gene_EYR40_007193/gene_EYR40_006395/gene_EYR40_010195/gene_EYR40_010671/gene_EYR40_006750/gene_EYR4

Category	GOID	Description	geneID	geneName	Count	Up	Up_Gene_id	Down	Down_Gene_id
			_EYR40_010195/gene_EYR40_011010/gene_EYR40_002317/gene_EYR40_004279/gene_EYR40_010671/gene_EYR40_006750/gene_EYR40_003410/gene_EYR40_002651/gene_EYR40_004793/gene_EYR40_009086	5_1/CHT1_2/EYR40_010671/EYR40_006750/EYR40_003410/EYR40_002651/EYR40_004793/CBH1_13			R40_002651/gene_EYR40_009086		0_004793
MF	GO:0050662	coenzyme binding	gene_EYR40_000292/gene_EYR40_008395/gene_EYR40_003345/gene_EYR40_005082/gene_EYR40_006810/gene_EYR40_000516/gene_EYR40_002937/gene_EYR40_007767/gene_EYR40_002871/gene_EY	EYR40_000292/ERG1_1/EYR40_003345/EYR40_005082/EYR40_006810/LCB2/EYR40_002937/EYR40_007767/HMG1/ZWF1/EYR40_009367/	46	22	gene_EYR40_008395/gene_EYR40_003345/gene_EYR40_006810/gene_EYR40_000516/gene_EYR40_007767/gene_EYR40_002871/gene_EY	24	gene_EYR40_000292/gene_EYR40_005082/gene_EYR40_002937/gene_EYR40_009367/gene_EYR40_005602/gene_EYR40_006607/gene_EYR

Category	GOID	Description	geneID	geneName	Count	Up	Up_Gene_id	Down	Down_Gene_id
			R40_005655/gene_EYR40_009367/gene_EYR40_003938/gene_EYR40_005602/gene_EYR40_006607/gene_EYR40_008889/gene_EYR40_004548/gene_EYR40_001709/gene_EYR40_000266/gene_EYR40_002845/novel.671/gene_EYR40_005784/gene_EYR40_011048/gene_EYR40_007794/gene_EYR40_006278/gene_EYR40_006606/gene_EYR40_002772/gene_EYR40_000519/gene_EYR40_006307/gene_EYR40_010542/gene_EYR40_	EYR40_003938/EYR40_005602/EYR40_006607/EYR40_008889/EYR40_004548/EYR40_001709/EYR40_000266/HIS5/-/EYR40_005784/EYR40_011048/EYR40_007794/EYR40_006278/EYR40_006606/EYR40_002772/UGD1/EYR40_006307/EYR40_010542/EYR40_009683/EYR40_007536/EYR40_008			R40_005655/gene_EYR40_003938/gene_EYR40_008889/gene_EYR40_000519/gene_EYR40_008770/gene_EYR40_000871/gene_EYR40_008971/gene_EYR40_003361/gene_EYR40_002527/gene_EYR40_009394/gene_EYR40_009837/gene_EYR40_001704/gene_EYR40_002931/gene_EYR40_00		40_004548/gene_EYR40_001709/gene_EYR40_000266/gene_EYR40_002845/novel.671/gene_EYR40_005784/gene_EYR40_011048/gene_EYR40_007794/gene_EYR40_006278/gene_EYR40_006606/gene_EYR40_002772/gene_EYR40_006307/gene_EYR40_010542/gene_EYR40_009683/gene_EYR4

Category	GOID	Description	geneID	geneName	Count	Up	Up_Gene_id	Down	Down_Gene_id
			009683/gene_EYR40_007536/gene_EYR40_008770/gene_EYR40_000871/gene_EYR40_008971/gene_EYR40_003361/gene_EYR40_002527/gene_EYR40_004699/gene_EYR40_009394/gene_EYR40_009837/gene_EYR40_001704/gene_EYR40_008385/gene_EYR40_002931/gene_EYR40_001622/gene_EYR40_001133/gene_EYR40_004535/gene_EYR40_001487	770/EYR40_000871/EYR40_008971/EYR40_003361/EYR40_002527/GRE2_2/HST2/EYR40_009837/EYR40_001704/EYR40_008385/EYR40_002931/EYR40_001622/EYR40_001133/EYR40_004535/EYR40_001487			1622/gene_EYR40_001133/gene_EYR40_004535		0_007536/gene_EYR40_004699/gene_EYR40_008385/gene_EYR40_001487
MF	GO:0046914	transition metal ion binding	gene_EYR40_003611/gene_EYR40_005024/gene_EYR40_001296/ge	EYR40_003611/EYR40_005024/EYR40_001296/	62	27	gene_EYR40_003611/gene_EYR40_004362/gen	35	gene_EYR40_005024/gene_EYR40_001296/gen

Category	GOID	Description	geneID	geneName	Count	Up	Up_Gene_id	Down	Down_Gene_id
			ne_EYR40_004977/gene_EYR40_009103/gene_EYR40_006266/gene_EYR40_008893/gene_EYR40_004362/gene_EYR40_002397/gene_EYR40_006224/gene_EYR40_008227/gene_EYR40_004976/gene_EYR40_008328/gene_EYR40_004262/gene_EYR40_000960/gene_EYR40_008002/gene_EYR40_007209/gene_EYR40_007640/gene_EYR40_007498/gene_EYR40_010694/gene_EYR40_002425/novel.193/gene_EYR40_008062/gene_E	EYR40_004977/EYR40_009103/EYR40_006266/EYR40_008893/ERG5/EYR40_002397/EYR40_006224/EYR40_008227/EYR40_004976/LCC2_1/EYR40_004262/EYR40_000960/ERG11/EYR40_007209/ERG3/URE1/EYR40_010694/ERG25/-/EYR40_008062/EYR40_011040/EYR40_006207/EYR40_010			e_EYR40_002397/gene_EYR40_008227/gene_EYR40_000960/gene_EYR40_008002/gene_EYR40_007209/gene_EYR40_007640/gene_EYR40_007498/gene_EYR40_002425/gene_EYR40_010868/gene_EYR40_009812/gene_EYR40_005259/gene_EYR40_007800/gene_EYR40_001983/gene_EYR		e_EYR40_004977/gene_EYR40_009103/gene_EYR40_006266/gene_EYR40_008893/gene_EYR40_006224/gene_EYR40_004976/gene_EYR40_008328/gene_EYR40_004262/gene_EYR40_010694/novel.193/gene_EYR40_008062/gene_EYR40_011040/gene_EYR40_006207/gene_EYR40_004789/nov

Category	GOID	Description	geneID	geneName	Count	Up	Up_Gene_id	Down	Down_Gene_id
			YR40_011040/gene_EYR40_006207/gene_EYR40_010868/gene_EYR40_004789/gene_EYR40_009812/gene_EYR40_005259/gene_EYR40_007800/gene_EYR40_001983/gene_EYR40_002853/novel.311/gene_EYR40_001477/gene_EYR40_002483/gene_EYR40_001421/gene_EYR40_000298/gene_EYR40_003202/novel.80/gene_EYR40_006678/gene_EYR40_001458/gene_EYR40_003261/gene_EYR40_004873/gene_EYR40_002791/g	868/EYR40_004789/EYR40_009812/MET6/DIT2_3/EYR40_001983/FET3/-/EYR40_001477/EYR40_002483/EYR40_001421/EYR40_000298/EYR40_003202/-/EYR40_006678/EYR40_001458/EYR40_003261/EYR40_004873/EYR40_002791/EYR40_004361/EYR40_007106/EYR40_000308/EYR			40_002853/gene_EYR40_004873/gene_EYR40_004361/gene_EYR40_007106/gene_EYR40_000308/gene_EYR40_004760/gene_EYR40_002241/gene_EYR40_001793/gene_EYR40_009813/gene_EYR40_010824/gene_EYR40_000255/gene_EYR40_004341		el.311/gene_EYR40_001477/gene_EYR40_002483/gene_EYR40_001421/gene_EYR40_000298/gene_EYR40_003202/novel.80/gene_EYR40_006678/gene_EYR40_001458/gene_EYR40_003261/gene_EYR40_002791/gene_EYR40_002794/gene_EYR40_001926/gene_EYR40_005382/gene_EYR40_0

Category	GOID	Description	geneID	geneName	Count	Up	Up_Gene_id	Down	Down_Gene_id
			ene_EYR40_004361/gene_EYR40_007106/gene_EYR40_000308/gene_EYR40_002794/gene_EYR40_004760/gene_EYR40_001926/gene_EYR40_005382/gene_EYR40_002241/gene_EYR40_006394/gene_EYR40_003270/gene_EYR40_006414/gene_EYR40_001793/gene_EYR40_009813/novel.51/gene_EYR40_002917/gene_EYR40_010824/gene_EYR40_000255/gene_EYR40_004341	40_002794/EYR40_004760/EYR40_001926/EYR40_005382/EYR40_002241/EYR40_006394/EYR40_003270/EYR40_006414/EYR40_001793/EYR40_009813/-/EYR40_002917/ADH1_2/EYR40_000255/EYR40_004341					06394/gene_EYR40_003270/gene_EYR40_006414/novel.51/gene_EYR40_002917
MF	GO:0016705	oxidoreductase	gene_EYR40_008395/gene_EYR40	ERG1_1/EYR40_009103	35	17	gene_EYR40_008395/ge	18	gene_EYR40_009103/ge

Category	GOID	Description	geneID	geneName	Count	Up	Up_Gene_id	Down	Down_Gene_id
		activity, acting on paired donors, with incorporation or reduction of molecular oxygen	_009103/gene_EYR40_006266/gene_EYR40_004362/gene_EYR40_008227/gene_EYR40_004262/gene_EYR40_008002/gene_EYR40_007209/novel.193/gene_EYR40_010868/gene_EYR40_009812/gene_EYR40_001709/gene_EYR40_007800/gene_EYR40_001983/novel.311/gene_EYR40_001477/gene_EYR40_002483/gene_EYR40_000298/novel.80/gene_EYR40_006678/gene_EYR40_001458/gene_EYR40_003261/gene_EYR40_00279	/EYR40_006266/ERG5/EYR40_008227/EYR40_004262/ERG11/EYR40_007209/-/EYR40_010868/EYR40_009812/EYR40_001709/DIT2_3/EYR40_001983/-/EYR40_001477/EYR40_002483/EYR40_000298/-/EYR40_006678/EYR40_001458/EYR40_003261/EYR40_002791/EYR40_004361/EYR			ne_EYR40_004362/gene_EYR40_008227/gene_EYR40_008002/gene_EYR40_007209/gene_EYR40_010868/gene_EYR40_009812/gene_EYR40_007800/gene_EYR40_001983/gene_EYR40_004361/gene_EYR40_007106/gene_EYR40_000308/gene_EYR40_002241/gene_EYR40_001793/gene_EY		ne_EYR40_006266/gene_EYR40_004262/novel.193/gene_EYR40_001709/novel.311/gene_EYR40_001477/gene_EYR40_002483/gene_EYR40_000298/novel.80/gene_EYR40_006678/gene_EYR40_001458/gene_EYR40_003261/gene_EYR40_002791/gene_EYR40_002794/gene_EYR40_003270/gene_

Category	GOID	Description	geneID	geneName	Count	Up	Up_Gene_id	Down	Down_Gene_id
			1/gene_EYR40_004361/gene_EYR40_007106/gene_EYR40_000308/gene_EYR40_002794/gene_EYR40_002241/gene_EYR40_003270/gene_EYR40_006414/gene_EYR40_001793/gene_EYR40_009813/gene_EYR40_002917/gene_EYR40_010824/gene_EYR40_000255	40_007106/EYR40_000308/EYR40_002794/EYR40_002241/EYR40_003270/EYR40_006414/EYR40_001793/EYR40_009813/EYR40_002917/ADH1_2/EYR40_000255			R40_009813/gene_EYR40_010824/gene_EYR40_000255		EYR40_006414/gene_EYR40_002917
MF	GO:0020037	heme binding	gene_EYR40_009103/gene_EYR40_006266/gene_EYR40_004362/gene_EYR40_008227/gene_EYR40_004262/gene_EYR40_008002/gene_EYR40_007209/n	EYR40_009103/EYR40_006266/ERG5/EYR40_008227/EYR40_004262/ERG11/EYR40_007209/-/EYR40_010	35	17	gene_EYR40_004362/gene_EYR40_008227/gene_EYR40_008002/gene_EYR40_007209/gene_EYR40_010	18	gene_EYR40_009103/gene_EYR40_006266/gene_EYR40_004262/novel.193/novel.311/gene_EYR40_001477

Category	GOID	Description	geneID	geneName	Count	Up	Up_Gene_id	Down	Down_Gene_id
			ovel.193/gene_EYR40_010868/gene_EYR40_009812/gene_EYR40_007800/gene_EYR40_001983/novel.311/gene_EYR40_001477/gene_EYR40_002483/gene_EYR40_000298/novel.80/gene_EYR40_006678/gene_EYR40_008971/gene_EYR40_001458/gene_EYR40_003261/gene_EYR40_002791/gene_EYR40_004361/gene_EYR40_010740/gene_EYR40_007106/gene_EYR40_000308/gene_EYR40_002794/gene_EYR40_002241/gene_	868/EYR40_009812/DIT2_3/EYR40_001983/-/EYR40_001477/EYR40_002483/EYR40_000298/-/EYR40_006678/EYR40_008971/EYR40_001458/EYR40_003261/EYR40_002791/EYR40_004361/VPL2_3/EYR40_007106/EYR40_000308/EYR40_002794/EYR40_002241/EYR40_003270/EYR40_006414/EYR			868/gene_EYR40_009812/gene_EYR40_007800/gene_EYR40_001983/gene_EYR40_008971/gene_EYR40_004361/gene_EYR40_007106/gene_EYR40_000308/gene_EYR40_002241/gene_EYR40_001793/gene_EYR40_009813/gene_EYR40_010824/gene_EYR40_000255		/gene_EYR40_002483/gene_EYR40_000298/novel.80/gene_EYR40_006678/gene_EYR40_001458/gene_EYR40_003261/gene_EYR40_002791/gene_EYR40_010740/gene_EYR40_002794/gene_EYR40_003270/gene_EYR40_006414/gene_EYR40_002917

Category	GOID	Description	geneID	geneName	Count	Up	Up_Gene_id	Down	Down_Gene_id
			EYR40_003270/gene_EYR40_006414/gene_EYR40_001793/gene_EYR40_009813/gene_EYR40_002917/gene_EYR40_010824/gene_EYR40_000255	40_001793/EYR40_009813/EYR40_002917/ADH1_2/EYR40_000255					
MF	GO:0046906	tetrapyrrole binding	gene_EYR40_009103/gene_EYR40_006266/gene_EYR40_004362/gene_EYR40_008227/gene_EYR40_004262/gene_EYR40_008002/gene_EYR40_007209/novel.193/gene_EYR40_010868/gene_EYR40_009812/gene_EYR40_007800/gene_EYR40_001983/novel.311/gene_EYR40	EYR40_009103/EYR40_006266/ERG5/EYR40_008227/EYR40_004262/ERG11/EYR40_007209/--/EYR40_010868/EYR40_009812/DIT2_3/EYR40_001983/--/EYR40_001477/EYR40_002483/EYR	35	17	gene_EYR40_004362/gene_EYR40_008227/gene_EYR40_008002/gene_EYR40_007209/gene_EYR40_010868/gene_EYR40_009812/gene_EYR40_007800/gene_EYR40_001983/gene_EYR4	18	gene_EYR40_009103/gene_EYR40_006266/gene_EYR40_004262/novel.193/novel.311/gene_EYR40_001477/gene_EYR40_002483/gene_EYR40_000298/novel.80/gene_EYR40_006678/gene_E

Category	GOID	Description	geneID	geneName	Count	Up	Up_Gene_id	Down	Down_Gene_id
			_001477/gene_EYR40_002483/gene_EYR40_000298/novel.80/gene_EYR40_006678/gene_EYR40_008971/gene_EYR40_001458/gene_EYR40_003261/gene_EYR40_002791/gene_EYR40_004361/gene_EYR40_010740/gene_EYR40_007106/gene_EYR40_000308/gene_EYR40_002794/gene_EYR40_002241/gene_EYR40_003270/gene_EYR40_006414/gene_EYR40_001793/gene_EYR40_009813/gene_EYR40_002917/gene_EYR40_010	40_000298/-/EYR40_006678/EYR40_008971/EYR40_001458/EYR40_003261/EYR40_002791/EYR40_004361/VPL2_3/EYR40_007106/EYR40_000308/EYR40_002794/EYR40_002241/EYR40_003270/EYR40_006414/EYR40_001793/EYR40_009813/EYR40_002917/ADH1_2/EYR40_000255			0_008971/gene_EYR40_004361/gene_EYR40_007106/gene_EYR40_000308/gene_EYR40_002241/gene_EYR40_001793/gene_EYR40_009813/gene_EYR40_010824/gene_EYR40_000255		YR40_001458/gene_EYR40_003261/gene_EYR40_002791/gene_EYR40_010740/gene_EYR40_002794/gene_EYR40_003270/gene_EYR40_006414/gene_EYR40_002917

Category	GOID	Description	geneID	geneName	Count	Up	Up_Gene_id	Down	Down_Gene_id
			824/gene_EYR40_000255						
MF	GO:0043169	cation binding	gene_EYR40_003611/gene_EYR40_005024/gene_EYR40_001296/gene_EYR40_004977/gene_EYR40_009103/gene_EYR40_006266/gene_EYR40_008893/gene_EYR40_004362/gene_EYR40_004417/gene_EYR40_002397/gene_EYR40_006224/gene_EYR40_008227/gene_EYR40_004976/gene_EYR40_008328/gene_EYR40_004262/gene_EYR40_000960/gene_EYR40_008002/gene_EYR40_008089/g	EYR40_003611/EYR40_005024/EYR40_001296/EYR40_004977/EYR40_009103/EYR40_006266/EYR40_008893/ERG5/EYR40_004417/EYR40_002397/EYR40_006224/EYR40_008227/EYR40_004976/LCC2_1/EYR40_004262/EYR40_000960/ERG11/FAS2/EYR40_007209/ERG3/UR	74	33	gene_EYR40_003611/gene_EYR40_004362/gene_EYR40_002397/gene_EYR40_008227/gene_EYR40_000960/gene_EYR40_008002/gene_EYR40_008089/gene_EYR40_007209/gene_EYR40_007640/gene_EYR40_007498/gene_EYR40_002425/gene_EYR40_010868/gene_	41	gene_EYR40_005024/gene_EYR40_001296/gene_EYR40_004977/gene_EYR40_009103/gene_EYR40_006266/gene_EYR40_008893/gene_EYR40_004417/gene_EYR40_006224/gene_EYR40_004976/gene_EYR40_008328/gene_EYR40_004262/gene_EYR40_010694/novel.1

Category	GOID	Description	geneID	geneName	Count	Up	Up_Gene_id	Down	Down_Gene_id
			ene_EYR40_007209/gene_EYR40_007640/gene_EYR40_007498/gene_EYR40_010694/gene_EYR40_002425/novel.193/gene_EYR40_009367/gene_EYR40_008062/gene_EYR40_011040/gene_EYR40_006207/gene_EYR40_001293/gene_EYR40_010868/gene_EYR40_004789/gene_EYR40_009812/gene_EYR40_003896/gene_EYR40_010822/gene_EYR40_005259/gene_EYR40_007800/gene_EYR40_001983/gene_EYR40_002853/novel.	E1/EYR40_010694/ERG25/-/EYR40_009367/EYR40_008062/EYR40_011040/EYR40_006207/EYR40_001293/EYR40_010868/EYR40_004789/EYR40_009812/PYK1/EYR40_010822/MET6/DIT2_3/EYR40_001983/FET3/-/EYR40_001477/EYR40_002483/EYR40_001421/EYR40_000298/EYR40_			EYR40_009812/gene_EYR40_003896/gene_EYR40_005259/gene_EYR40_007800/gene_EYR40_001983/gene_EYR40_002853/gene_EYR40_000019/gene_EYR40_004873/gene_EYR40_004361/gene_EYR40_007106/gene_EYR40_003318/gene_EYR40_000308/gene_EYR40_004760/gene_EYR40_		93/gene_EYR40_009367/gene_EYR40_008062/gene_EYR40_011040/gene_EYR40_006207/gene_EYR40_001293/gene_EYR40_004789/gene_EYR40_010822/novel.311/gene_EYR40_001477/gene_EYR40_002483/gene_EYR40_001421/gene_EYR40_000298/gene_EYR40_003202/novel.80/gene_EYR

Category	GOID	Description	geneID	geneName	Count	Up	Up_Gene_id	Down	Down_Gene_id
			311/gene_EYR40_001477/gene_EYR40_002483/gene_EYR40_001421/gene_EYR40_000298/gene_EYR40_003202/novel.80/gene_EYR40_006678/gene_EYR40_001458/gene_EYR40_000019/gene_EYR40_003261/gene_EYR40_004873/gene_EYR40_002791/gene_EYR40_004361/gene_EYR40_008131/gene_EYR40_007106/gene_EYR40_003318/gene_EYR40_000308/gene_EYR40_002794/gene_EYR40_004760/gene_EYR40_001926/	003202/-/EYR40_006678/EYR40_001458/EYR40_000019/EYR40_003261/EYR40_004873/EYR40_002791/EYR40_004361/CAR1_2/EYR40_007106/EYR40_003318/EYR40_000308/EYR40_002794/EYR40_004760/EYR40_001926/EYR40_005382/EYR40_002241/MNS1B_2/EYR40_006394/EYR40_003			002241/gene_EYR40_011010/gene_EYR40_001793/gene_EYR40_009813/gene_EYR40_009487/gene_EYR40_010824/gene_EYR40_000255/gene_EYR40_004341		40_006678/gene_EYR40_001458/gene_EYR40_003261/gene_EYR40_002791/gene_EYR40_008131/gene_EYR40_002794/gene_EYR40_001926/gene_EYR40_005382/gene_EYR40_006394/gene_EYR40_003270/gene_EYR40_006414/novel.51/gene_EYR40_002917/gene_EYR40_004944

Category	GOID	Description	geneID	geneName	Count	Up	Up_Gene_id	Down	Down_Gene_id
			gene_EYR40_005382/gene_EYR40_002241/gene_EYR40_011010/gene_EYR40_006394/gene_EYR40_003270/gene_EYR40_006414/gene_EYR40_001793/gene_EYR40_009813/novel.51/gene_EYR40_002917/gene_EYR40_004944/gene_EYR40_009487/gene_EYR40_010824/gene_EYR40_000255/gene_EYR40_004341	270/EYR40_006414/EYR40_001793/EYR40_009813/-/EYR40_002917/EYR40_004944/COP3_2/ADH1_2/EYR40_000255/EYR40_004341					
MF	GO:0046872	metal ion binding	gene_EYR40_003611/gene_EYR40_005024/gene_EYR40_001296/gene_EYR40_004977/gene_EYR40_0	EYR40_003611/EYR40_005024/EYR40_001296/EYR40_004977/EYR40_	73	32	gene_EYR40_003611/gene_EYR40_004362/gene_EYR40_002397/gene	41	gene_EYR40_005024/gene_EYR40_001296/gene_EYR40_004977/gene

Category	GOID	Description	geneID	geneName	Count	Up	Up_Gene_id	Down	Down_Gene_id
			09103/gene_EYR40_006266/gene_EYR40_008893/gene_EYR40_004362/gene_EYR40_004417/gene_EYR40_002397/gene_EYR40_006224/gene_EYR40_008227/gene_EYR40_004976/gene_EYR40_008328/gene_EYR40_004262/gene_EYR40_000960/gene_EYR40_008002/gene_EYR40_008089/gene_EYR40_007209/gene_EYR40_007640/gene_EYR40_007498/gene_EYR40_010694/gene_EYR40_002425/novel.193/gene_EYR40_00936	009103/EYR40_006266/EYR40_008893/ERG5/EYR40_004417/EYR40_002397/EYR40_006224/EYR40_008227/EYR40_004976/LCC2_1/EYR40_004262/EYR40_000960/ERG11/FAS2/EYR40_007209/ERG3/URE1/EYR40_010694/ERG25/-/EYR40_009367/EYR40_008062/EYR40_011040/EYR40_006			_EYR40_008227/gene_EYR40_000960/gene_EYR40_008002/gene_EYR40_008089/gene_EYR40_007209/gene_EYR40_007640/gene_EYR40_007498/gene_EYR40_002425/gene_EYR40_010868/gene_EYR40_009812/gene_EYR40_003896/gene_EYR40_005259/gene_EYR40_007800/gene_EYR4		_EYR40_009103/gene_EYR40_006266/gene_EYR40_008893/gene_EYR40_004417/gene_EYR40_006224/gene_EYR40_004976/gene_EYR40_008328/gene_EYR40_004262/gene_EYR40_010694/novel.193/gene_EYR40_009367/gene_EYR40_008062/gene_EYR40_011040/gene_EYR40_006207/gene

Category	GOID	Description	geneID	geneName	Count	Up	Up_Gene_id	Down	Down_Gene_id
			7/gene_EYR40_008062/gene_EYR40_011040/gene_EYR40_006207/gene_EYR40_001293/gene_EYR40_010868/gene_EYR40_004789/gene_EYR40_009812/gene_EYR40_003896/gene_EYR40_010822/gene_EYR40_005259/gene_EYR40_007800/gene_EYR40_001983/gene_EYR40_002853/novel.311/gene_EYR40_001477/gene_EYR40_002483/gene_EYR40_001421/gene_EYR40_000298/gene_EYR40_003202/novel.80/gene_EYR40_	207/EYR40_001293/EYR40_010868/EYR40_004789/EYR40_009812/PYK1/EYR40_010822/MET6/DIT2_3/EYR40_001983/FET3/-/EYR40_001477/EYR40_002483/EYR40_001421/EYR40_000298/EYR40_003202/-/EYR40_006678/EYR40_001458/EYR40_003261/EYR40_004873/EYR40_002791/EYR			0_001983/gene_EYR40_002853/gene_EYR40_004873/gene_EYR40_004361/gene_EYR40_007106/gene_EYR40_003318/gene_EYR40_000308/gene_EYR40_004760/gene_EYR40_002241/gene_EYR40_011010/gene_EYR40_001793/gene_EYR40_009813/gene_EYR40_009487/gene_EYR40_01082		_EYR40_001293/gene_EYR40_004789/gene_EYR40_010822/novel.311/gene_EYR40_001477/gene_EYR40_002483/gene_EYR40_001421/gene_EYR40_000298/gene_EYR40_003202/novel.80/gene_EYR40_006678/gene_EYR40_001458/gene_EYR40_003261/gene_EYR40_002791/gene_EYR40_00

Category	GOID	Description	geneID	geneName	Count	Up	Up_Gene_id	Down	Down_Gene_id
			006678/gene_EYR40_001458/gene_EYR40_003261/gene_EYR40_004873/gene_EYR40_002791/gene_EYR40_004361/gene_EYR40_008131/gene_EYR40_007106/gene_EYR40_003318/gene_EYR40_000308/gene_EYR40_002794/gene_EYR40_004760/gene_EYR40_001926/gene_EYR40_005382/gene_EYR40_002241/gene_EYR40_011010/gene_EYR40_006394/gene_EYR40_003270/gene_EYR40_006414/gene_EYR40_001793/gene_	40_004361/CAR1_2/EYR40_007106/EYR40_003318/EYR40_000308/EYR40_002794/EYR40_004760/EYR40_001926/EYR40_005382/EYR40_002241/MNS1B_2/EYR40_006394/EYR40_003270/EYR40_006414/EYR40_001793/EYR40_009813/-/EYR40_002917/EYR40_004944/COP3_2/ADH1_2/EYR40_0			4/gene_EYR40_000255/gene_EYR40_004341		8131/gene_EYR40_002794/gene_EYR40_001926/gene_EYR40_005382/gene_EYR40_006394/gene_EYR40_003270/gene_EYR40_006414/novel.51/gene_EYR40_002917/gene_EYR40_004944

Category	GOID	Description	geneID	geneName	Count	Up	Up_Gene_id	Down	Down_Gene_id
			EYR40_009813/novel.51/gene_EYR40_002917/gene_EYR40_004944/gene_EYR40_009487/gene_EYR40_010824/gene_EYR40_000255/gene_EYR40_004341	00255/EYR40_004341					
**GO enrichment between 5 and B10 samples**									
CC	GO:0016021	integral component of membrane	gene_EYR40_009863/gene_EYR40_010044/gene_EYR40_009134/gene_EYR40_008326/gene_EYR40_007784/gene_EYR40_008395/gene_EYR40_007055/gene_EYR40_009862/gene_EYR40_006760/gene_EYR40_005038/gene_EYR40_001205/	EYR40_009863/EYR40_010044/EYR40_009134/EYR40_008326/ERG28/ERG1_1/EYR40_007055/EYR40_009862/EYR40_006760/EYR40_005038/EYR40_001205/EYR40_	43	22	gene_EYR40_009863/gene_EYR40_010044/gene_EYR40_008326/gene_EYR40_007784/gene_EYR40_007055/gene_EYR40_009862/gene_EYR40_005038/gene_EYR	21	gene_EYR40_009134/gene_EYR40_008395/gene_EYR40_006760/gene_EYR40_007606/gene_EYR40_006155/gene_EYR40_002854/gene_EYR40_002030/gene_EYR4

Category	GOID	Description	geneID	geneName	Count	Up	Up_Gene_id	Down	Down_Gene_id
			gene_EYR40_008	008424/EYR			40_001205/		0_004402/ge
			424/gene_EYR40	40_010842/			gene_EYR4		ne_EYR40_
			_010842/gene_E	EYR40_007			0_008424/ge		004757/gen
			YR40_007606/ge	606/EYR40_			ne_EYR40_		e_EYR40_0
			ne_EYR40_00653	006536/EYR			010842/gen		07431/gene
			6/gene_EYR40_0	40_006155/			e_EYR40_0		_EYR40_00
			06155/gene_EYR	FTR1/EYR4			06536/gene		3560/gene_
			40_002854/gene_	0_006361/E			_EYR40_00		EYR40_009
			EYR40_006361/g	YR40_00203			6361/gene_		492/gene_E
			ene_EYR40_0020	0/PHO84_2			EYR40_007		YR40_00540
			30/gene_EYR40_	/EYR40_004			818/gene_E		8/gene_EYR
			007818/gene_EY	402/EYR40_			YR40_00488		40_004232/
			R40_004402/gene	004880/EYR			0/gene_EY		gene_EYR4
			_EYR40_004880/	40_004303/			R40_004303		0_006647/ge
			gene_EYR40_004	EYR40_004			/gene_EYR		ne_EYR40_
			303/gene_EYR40	757/EYR40_			40_006133/		004839/gen
			_004757/gene_E	007431/EYR			gene_EYR4		e_EYR40_0
			YR40_007431/ge	40_003560/			0_003020/ge		08348/gene
			ne_EYR40_00356	MDR1_1/E			ne_EYR40_		_EYR40_00
			0/gene_EYR40_0	YR40_00302			008523/gen		5087/gene_
			06133/gene_EYR	0/EYR40_00			e_EYR40_0		EYR40_003
			40_003020/gene_	8523/EYR40			10360/gene		559/gene_E
			EYR40_008523/g	_009492/EY			_EYR40_00		YR40_00508
			ene_EYR40_0094	R40_005408			6360/gene_		6/gene_EYR
			92/gene_EYR40_	/EYR40_010			EYR40_002		40_008579

Category	GOID	Description	geneID	geneName	Count	Up	Up_Gene_id	Down	Down_Gene_id
			005408/gene_EYR40_010360/gene_EYR40_006360/gene_EYR40_004232/gene_EYR40_006647/gene_EYR40_002490/gene_EYR40_009740/gene_EYR40_004839/gene_EYR40_008348/gene_EYR40_005087/gene_EYR40_003559/gene_EYR40_005086/gene_EYR40_008579	360/EYR40_006360/EYR40_004232/EYR40_006647/EYR40_002490/EYR40_009740/EYR40_004839/EYR40_008348/EYR40_005087/EYR40_003559/EYR40_005086/EYR40_008579			490/gene_EYR40_009740		
CC	GO:0031224	intrinsic component of membrane	gene_EYR40_009863/gene_EYR40_010044/gene_EYR40_009134/gene_EYR40_008326/gene_EYR40_007784/gene_EYR40_008395/gene_EYR40_007055/g	EYR40_009863/EYR40_010044/EYR40_009134/EYR40_008326/ERG28/ERG1_1/EYR40_007055/EYR40_009	43	22	gene_EYR40_009863/gene_EYR40_010044/gene_EYR40_008326/gene_EYR40_007784/gene_EYR40_007	21	gene_EYR40_009134/gene_EYR40_008395/gene_EYR40_006760/gene_EYR40_007606/gene_EYR40_006

Category	GOID	Description	geneID	geneName	Count	Up	Up_Gene_id	Down	Down_Gene_id
			ene_EYR40_009862/gene_EYR40_006760/gene_EYR40_005038/gene_EYR40_001205/gene_EYR40_008424/gene_EYR40_010842/gene_EYR40_007606/gene_EYR40_006536/gene_EYR40_006155/gene_EYR40_002854/gene_EYR40_006361/gene_EYR40_002030/gene_EYR40_007818/gene_EYR40_004402/gene_EYR40_004880/gene_EYR40_004303/gene_EYR40_004757/gene_EYR40_007431/gene_EYR40_003560/gene_EYR40_0	862/EYR40_006760/EYR40_005038/EYR40_001205/EYR40_008424/EYR40_010842/EYR40_007606/EYR40_006536/EYR40_006155/FTR1/EYR40_006361/EYR40_002030/PHO84_2/EYR40_004402/EYR40_004880/EYR40_004303/EYR40_004757/EYR40_007431/EYR40_003560/MDR1_1/EYR40_00302			055/gene_EYR40_009862/gene_EYR40_005038/gene_EYR40_001205/gene_EYR40_008424/gene_EYR40_010842/gene_EYR40_006536/gene_EYR40_006361/gene_EYR40_007818/gene_EYR40_004880/gene_EYR40_004303/gene_EYR40_006133/gene_EYR40_003020/gene_EYR40_008523/gen		155/gene_EYR40_002854/gene_EYR40_002030/gene_EYR40_004402/gene_EYR40_004757/gene_EYR40_007431/gene_EYR40_003560/gene_EYR40_009492/gene_EYR40_005408/gene_EYR40_004232/gene_EYR40_006647/gene_EYR40_004839/gene_EYR40_008348/gene_EYR40_005087/gene_

Category	GOID	Description	geneID	geneName	Count	Up	Up_Gene_id	Down	Down_Gene_id
			06133/gene_EYR40_003020/gene_EYR40_008523/gene_EYR40_009492/gene_EYR40_005408/gene_EYR40_010360/gene_EYR40_006360/gene_EYR40_004232/gene_EYR40_006647/gene_EYR40_002490/gene_EYR40_009740/gene_EYR40_004839/gene_EYR40_008348/gene_EYR40_005087/gene_EYR40_003559/gene_EYR40_005086/gene_EYR40_008579	0/EYR40_008523/EYR40_009492/EYR40_005408/EYR40_010360/EYR40_006360/EYR40_004232/EYR40_006647/EYR40_002490/EYR40_009740/EYR40_004839/EYR40_008348/EYR40_005087/EYR40_003559/EYR40_005086/EYR40_008579			e_EYR40_010360/gene_EYR40_006360/gene_EYR40_002490/gene_EYR40_009740		EYR40_003559/gene_EYR40_005086/gene_EYR40_008579
MF	GO:0048037	cofactor binding	gene_EYR40_000292/gene_EYR40_009103/gene_EYR40_006266/ge	EYR40_000292/EYR40_009103/EYR40_006266/	78	41	gene_EYR40_000292/gene_EYR40_009103/gen	37	gene_EYR40_003392/gene_EYR40_008395/gen

Category	GOID	Description	geneID	geneName	Count	Up	Up_Gene_id	Down	Down_Gene_id
			ne_EYR40_000294/gene_EYR40_003392/gene_EYR40_008395/gene_EYR40_002871/gene_EYR40_005082/gene_EYR40_008227/gene_EYR40_009367/gene_EYR40_005655/gene_EYR40_008200/gene_EYR40_006307/gene_EYR40_002937/gene_EYR40_005526/gene_EYR40_011048/gene_EYR40_010848/gene_EYR40_010114/gene_EYR40_008889/gene_EYR40_004262/gene_EYR40_009812/gene_EYR40_010244/gene_EYR40_010	EYR40_000294/EYR40_003392/ERG1_1/HMG1/EYR40_005082/EYR40_008227/EYR40_009367/ZWF1/EYR40_008200/EYR40_006307/EYR40_002937/HEM1/EYR40_011048/EYR40_010848/EYR40_010114/EYR40_008889/EYR40_004262/EYR40_009812/ALO1/EYR40_010868/EYR40_002791/EYR			e_EYR40_006266/gene_EYR40_000294/gene_EYR40_005082/gene_EYR40_009367/gene_EYR40_006307/gene_EYR40_002937/gene_EYR40_011048/gene_EYR40_010848/gene_EYR40_010114/gene_EYR40_004262/gene_EYR40_010244/gene_EYR40_002791/gene_EYR40_000270/novel.671/		e_EYR40_002871/gene_EYR40_008227/gene_EYR40_005655/gene_EYR40_008200/gene_EYR40_005526/gene_EYR40_008889/gene_EYR40_009812/gene_EYR40_010868/gene_EYR40_001793/gene_EYR40_003938/gene_EYR40_001856/gene_EYR40_008971/gene_EYR40_001983/gene_EYR40_

Category	GOID	Description	geneID	geneName	Count	Up	Up_Gene_id	Down	Down_Gene_id
			868/gene_EYR40_002791/gene_EYR40_000270/novel.671/gene_EYR40_001793/gene_EYR40_010841/gene_EYR40_009683/gene_EYR40_003938/gene_EYR40_001856/gene_EYR40_006607/gene_EYR40_008971/gene_EYR40_001715/gene_EYR40_001458/gene_EYR40_010855/gene_EYR40_000266/gene_EYR40_001983/gene_EYR40_005602/gene_EYR40_003054/gene_EYR40_002527/gene_EYR40_006248/gene_EYR40_00667	40_000270/-/EYR40_001793/EYR40_010841/EYR40_009683/EYR40_003938/EYR40_001856/EYR40_006607/EYR40_008971/EYR40_001715/EYR40_001458/EYR40_010855/EYR40_000266/EYR40_001983/EYR40_005602/EYR40_003054/EYR40_002527/EYR40_006248/EYR40_006678/EYR40_000871/-			gene_EYR40_010841/gene_EYR40_009683/gene_EYR40_006607/gene_EYR40_001715/gene_EYR40_001458/gene_EYR40_010855/gene_EYR40_000266/gene_EYR40_005602/gene_EYR40_006678/novel.80/gene_EYR40_002485/gene_EYR40_006278/gene_EYR40_002483/gene_EYR40_00660		003054/gene_EYR40_002527/gene_EYR40_006248/gene_EYR40_000871/gene_EYR40_000255/gene_EYR40_002241/gene_EYR40_002733/gene_EYR40_009837/gene_EYR40_001622/gene_EYR40_000308/gene_EYR40_010085/gene_EYR40_006009/gene_EYR40_010824/gene_EYR40_001841/ge

Category	GOID	Description	geneID	geneName	Count	Up	Up_Gene_id	Down	Down_Gene_id
			8/gene_EYR40_000871/novel.80/gene_EYR40_002485/gene_EYR40_006278/gene_EYR40_002483/gene_EYR40_006606/gene_EYR40_008541/gene_EYR40_000255/gene_EYR40_002241/gene_EYR40_009800/gene_EYR40_002733/gene_EYR40_009837/gene_EYR40_001622/gene_EYR40_000308/gene_EYR40_010085/gene_EYR40_006009/gene_EYR40_010542/gene_EYR40_010824/gene_EYR40_001841/gene_EYR40_007536/ge	/EYR40_002485/EYR40_006278/EYR40_002483/EYR40_006606/EYR40_008541/EYR40_000255/EYR40_002241/EYR40_009800/EYR40_002733/EYR40_009837/EYR40_001622/EYR40_000308/EYR40_010085/CAT1/EYR40_010542/ADH1_2/6PGD/EYR40_007536/EYR40_002917/EYR40_006191/GL			6/gene_EYR40_008541/gene_EYR40_009800/gene_EYR40_010542/gene_EYR40_007536/gene_EYR40_002917/gene_EYR40_006191/gene_EYR40_003270/gene_EYR40_002805/gene_EYR40_008385/gene_EYR40_002748/gene_EYR40_001479		ne_EYR40_001374/gene_EYR40_004873/gene_EYR40_002470/gene_EYR40_002931/gene_EYR40_007106/gene_EYR40_009422/gene_EYR40_008225/gene_EYR40_006535

Category	GOID	Description	geneID	geneName	Count	Up	Up_Gene_id	Down	Down_Gene_id
			ne_EYR40_002917/gene_EYR40_006191/gene_EYR40_001374/gene_EYR40_004873/gene_EYR40_002470/gene_EYR40_002931/gene_EYR40_003270/gene_EYR40_007106/gene_EYR40_009422/gene_EYR40_002805/gene_EYR40_008385/gene_EYR40_008225/gene_EYR40_006535/gene_EYR40_002748/gene_EYR40_001479	R1/EYR40_004873/EYR40_002470/EYR40_002931/EYR40_003270/EYR40_007106/EYR40_009422/EYR40_002805/EYR40_008385/EYR40_008225/FDH1_2/EYR40_002748/EYR40_001479					
**GO enrichment between CK and A5 samples**									
BP	GO:0043603	cellular amide metabolic process	gene_EYR40_009786/gene_EYR40_001164/gene_EYR40_008609/novel.72/gene_EYR	RPL44/CDC60/PAN6/-/RPL12/RPL19C/RPS1/RPL34/RPS	43	4	gene_EYR40_001164/gene_EYR40_008609/gene_EYR40_0	39	gene_EYR40_009786/novel.72/gene_EYR40_006753/gene

Category	GOID	Description	geneID	geneName	Count	Up	Up_Gene_id	Down	Down_Gene_id
			40_006753/gene_EYR40_004739/gene_EYR40_005367/gene_EYR40_000160/gene_EYR40_005781/gene_EYR40_004053/novel.987/gene_EYR40_003681/novel.17/novel.672/gene_EYR40_000981/gene_EYR40_009787/gene_EYR40_003980/gene_EYR40_000760/gene_EYR40_003344/gene_EYR40_000364/gene_EYR40_005589/gene_EYR40_005275/gene_EYR40_000166/gene_EYR40_005223/gene_EYR40_005642/gene_EYR40_006	17/EYR40_004053/-/EYR40_003681/-/-/RPL26B/RPL25/EYR40_003980/EYR40_000760/EYR40_003344/RPL20/RPL3/EYR40_005275/RPL31/RPL5/RPL35/RPS26/RPL2A/RPS19/RPS16/EYR40_004866/RPL13/RPS29/EYR40_004013/EYR40_010083/URE1/RPS22/RPS2/EYR40_006263/MSW1			07498/gene_EYR40_001520		_EYR40_004739/gene_EYR40_005367/gene_EYR40_000160/gene_EYR40_005781/gene_EYR40_004053/novel.987/gene_EYR40_003681/novel.17/novel.672/gene_EYR40_000981/gene_EYR40_009787/gene_EYR40_003980/gene_EYR40_000760/gene_EYR40_003344/gene_EYR40_000364/gene_

Category	GOID	Description	geneID	geneName	Count	Up	Up_Gene_id	Down	Down_Gene_id
			315/gene_EYR40_005093/gene_EYR40_003570/gene_EYR40_003941/gene_EYR40_004866/gene_EYR40_003934/gene_EYR40_004430/gene_EYR40_004013/gene_EYR40_010083/gene_EYR40_007498/gene_EYR40_005782/gene_EYR40_006651/gene_EYR40_006263/gene_EYR40_001520/gene_EYR40_010814/gene_EYR40_000677/gene_EYR40_006538/gene_EYR40_008794	/SES1/TMA22/ILS1/RPS15					EYR40_005589/gene_EYR40_005275/gene_EYR40_000166/gene_EYR40_005223/gene_EYR40_005642/gene_EYR40_006315/gene_EYR40_005093/gene_EYR40_003570/gene_EYR40_003941/gene_EYR40_004866/gene_EYR40_003934/gene_EYR40_004430/gene_EYR40_004013/gene_EYR40_01

Category	GOID	Description	geneID	geneName	Count	Up	Up_Gene_id	Down	Down_Gene_id
									0083/gene_EYR40_005782/gene_EYR40_006651/gene_EYR40_006263/gene_EYR40_010814/gene_EYR40_000677/gene_EYR40_006538/gene_EYR40_008794
BP	GO:0006412	translation	gene_EYR40_009786/gene_EYR40_001164/novel.72/gene_EYR40_006753/gene_EYR40_004739/gene_EYR40_005367/gene_EYR40_000160/gene_EYR40_005781/gene_EYR40_004053/novel.	RPL44/CDC60/-/RPL12/RPL19C/RPS1/RPL34/RPS17/EYR40_004053/-/-/-/RPL26B/RPL25/EYR40_003980/EYR40_00076	39	2	gene_EYR40_001164/gene_EYR40_001520	37	gene_EYR40_009786/novel.72/gene_EYR40_006753/gene_EYR40_004739/gene_EYR40_005367/gene_EYR40_000160/gene_EYR

Category	GOID	Description	geneID	geneName	Count	Up	Up_Gene_id	Down	Down_Gene_id
			987/novel.17/novel.672/gene_EYR40_000981/gene_EYR40_009787/gene_EYR40_003980/gene_EYR40_000760/gene_EYR40_003344/gene_EYR40_000364/gene_EYR40_005589/gene_EYR40_005275/gene_EYR40_000166/gene_EYR40_005223/gene_EYR40_005642/gene_EYR40_006315/gene_EYR40_005093/gene_EYR40_003570/gene_EYR40_003941/gene_EYR40_004866/gene_EYR40_003934/gene_EYR40_004430/gene_EYR40	0/EYR40_003344/RPL20/RPL3/EYR40_005275/RPL31/RPL5/RPL35/RPS26/RPL2A/RPS19/RPS16/EYR40_004866/RPL13/RPS29/EYR40_004013/RPS22/RPS2/EYR40_006263/MSW1/SES1/TMA22/ILS1/RPS15					40_005781/gene_EYR40_004053/novel.987/novel.17/novel.672/gene_EYR40_000981/gene_EYR40_009787/gene_EYR40_003980/gene_EYR40_000760/gene_EYR40_003344/gene_EYR40_000364/gene_EYR40_005589/gene_EYR40_005275/gene_EYR40_000166/gene_EYR40_005223/gene_EYR40_

Category	GOID	Description	geneID	geneName	Count	Up	Up_Gene_id	Down	Down_Gene_id
			_004013/gene_EYR40_005782/gene_EYR40_006651/gene_EYR40_006263/gene_EYR40_001520/gene_EYR40_010814/gene_EYR40_000677/gene_EYR40_006538/gene_EYR40_008794						005642/gene_EYR40_006315/gene_EYR40_005093/gene_EYR40_003570/gene_EYR40_003941/gene_EYR40_004866/gene_EYR40_003934/gene_EYR40_004430/gene_EYR40_004013/gene_EYR40_005782/gene_EYR40_006651/gene_EYR40_006263/gene_EYR40_010814/gene_EYR40_000677/ge

Category	GOID	Description	geneID	geneName	Count	Up	Up_Gene_id	Down	Down_Gene_id
									ne_EYR40_006538/gene_EYR40_008794
BP	GO:0006260	DNA replication	gene_EYR40_001082/gene_EYR40_000562/gene_EYR40_001920/gene_EYR40_001337/gene_EYR40_000744/gene_EYR40_002255/gene_EYR40_000883/gene_EYR40_010303/gene_EYR40_004337/gene_EYR40_004298/gene_EYR40_000252/gene_EYR40_009378/gene_EYR40_010764/gene_EYR40_008614/gene_EYR40_008110/gene_EYR40_000153	EYR40_001082/MCM7/POL2/EYR40_001337/EYR40_000744/RFA1_2/RFA1_1/EYR40_010303/MCM3/MCM5/EYR40_000252/CDC22/EYR40_010764/RFC3/RFC5/POL30	16	15	gene_EYR40_001082/gene_EYR40_000562/gene_EYR40_001920/gene_EYR40_001337/gene_EYR40_000744/gene_EYR40_000883/gene_EYR40_010303/gene_EYR40_004337/gene_EYR40_004298/gene_EYR40_000252/gene_EYR40_009378/gene	1	gene_EYR40_002255

Category	GOID	Description	geneID	geneName	Count	Up	Up_Gene_id	Down	Down_Gene_id
							_EYR40_010764/gene_EYR40_008614/gene_EYR40_008110/gene_EYR40_000153		
**GO enrichment between CK and B5 samples**									
CC	GO:0016021	integral component of membrane	gene_EYR40_007606/gene_EYR40_006360/gene_EYR40_006760/gene_EYR40_002267/gene_EYR40_008579/gene_EYR40_006157/gene_EYR40_001574/gene_EYR40_009212/gene_EYR40_004512/gene_EYR40_001205/gene_EYR40_007351/gene_EYR40_009863/gene_EYR40_003499/gene_E	EYR40_007606/EYR40_006360/EYR40_006760/HXT1_1/EYR40_008579/EYR40_006157/EYR40_001574/EYR40_009212/EYR40_004512/EYR40_001205/EYR40_007351/EYR40_009863/EYR40_003499/EYR	46	25	gene_EYR40_006360/gene_EYR40_006157/gene_EYR40_001574/gene_EYR40_009212/gene_EYR40_001205/gene_EYR40_007351/gene_EYR40_009863/gene_EYR40_003993/gene_EYR40_006361/ge	21	gene_EYR40_007606/gene_EYR40_006760/gene_EYR40_002267/gene_EYR40_008579/gene_EYR40_004512/gene_EYR40_003499/gene_EYR40_009186/gene_EYR40_007431/gene_EYR40_005568/gen

Category	GOID	Description	geneID	geneName	Count	Up	Up_Gene_id	Down	Down_Gene_id
			YR40_003993/gene_EYR40_009186/gene_EYR40_006361/gene_EYR40_009862/gene_EYR40_007818/gene_EYR40_007431/gene_EYR40_005568/gene_EYR40_001771/gene_EYR40_009694/gene_EYR40_004757/gene_EYR40_010365/gene_EYR40_000179/gene_EYR40_002490/gene_EYR40_000254/gene_EYR40_009492/gene_EYR40_007944/gene_EYR40_003020/gene_EYR40_002854/gene_EYR40_007784/gene_EYR40_005038/	40_003993/EYR40_009186/EYR40_006361/EYR40_009862/PHO84_2/EYR40_007431/EYR40_005568/EYR40_001771/EYR40_009694/EYR40_004757/EYR40_010365/EYR40_000179/EYR40_002490/EYR40_000254/EYR40_009492/EYR40_007944/EYR40_003020/FTR1/ERG28/EYR40_005038/EYR40_00			ne_EYR40_009862/gene_EYR40_007818/gene_EYR40_000179/gene_EYR40_002490/gene_EYR40_007944/gene_EYR40_003020/gene_EYR40_007784/gene_EYR40_005038/gene_EYR40_007055/gene_EYR40_009912/gene_EYR40_010393/gene_EYR40_007546/gene_EYR40_000091/gene_EY		e_EYR40_001771/gene_EYR40_009694/gene_EYR40_004757/gene_EYR40_010365/gene_EYR40_000254/gene_EYR40_009492/gene_EYR40_002854/gene_EYR40_007792/gene_EYR40_005087/gene_EYR40_005086/gene_EYR40_004232/gene_EYR40_002030

Category	GOID	Description	geneID	geneName	Count	Up	Up_Gene_id	Down	Down_Gene_id
			gene_EYR40_007055/gene_EYR40_009912/gene_EYR40_010393/gene_EYR40_007546/gene_EYR40_007792/gene_EYR40_000091/gene_EYR40_005087/gene_EYR40_005086/gene_EYR40_008240/gene_EYR40_004232/gene_EYR40_007317/gene_EYR40_007618/gene_EYR40_002030	7055/EYR40_009912/EYR40_010393/EYR40_007546/EYR40_007792/EYR40_000091/EYR40_005087/EYR40_005086/EYR40_008240/EYR40_004232/EYR40_007317/EYR40_007618/EYR40_002030			R40_008240/gene_EYR40_007317/gene_EYR40_007618		
CC	GO:0031224	intrinsic component of membrane	gene_EYR40_007606/gene_EYR40_006360/gene_EYR40_006760/gene_EYR40_002267/gene_EYR40_008579/gene_EYR40_006157/gene_	EYR40_007606/EYR40_006360/EYR40_006760/HXT1_1/EYR40_008579/EYR40_006157/EYR40_	46	25	gene_EYR40_006360/gene_EYR40_006157/gene_EYR40_001574/gene_EYR40_009212/gene_	21	gene_EYR40_007606/gene_EYR40_006760/gene_EYR40_002267/gene_EYR40_008579/gene_

Category	GOID	Description	geneID	geneName	Count	Up	Up_Gene_id	Down	Down_Gene_id
			EYR40_001574/gene_EYR40_009212/gene_EYR40_004512/gene_EYR40_001205/gene_EYR40_007351/gene_EYR40_009863/gene_EYR40_003499/gene_EYR40_003993/gene_EYR40_009186/gene_EYR40_006361/gene_EYR40_009862/gene_EYR40_007818/gene_EYR40_007431/gene_EYR40_005568/gene_EYR40_001771/gene_EYR40_009694/gene_EYR40_004757/gene_EYR40_010365/gene_EYR40_000179/gene_EYR40_00249	001574/EYR40_009212/EYR40_004512/EYR40_001205/EYR40_007351/EYR40_009863/EYR40_003499/EYR40_003993/EYR40_009186/EYR40_006361/EYR40_009862/PHO84_2/EYR40_007431/EYR40_005568/EYR40_001771/EYR40_009694/EYR40_004757/EYR40_010365/EYR40_000179/EYR40_002			EYR40_001205/gene_EYR40_007351/gene_EYR40_009863/gene_EYR40_003993/gene_EYR40_006361/gene_EYR40_009862/gene_EYR40_007818/gene_EYR40_000179/gene_EYR40_002490/gene_EYR40_007944/gene_EYR40_003020/gene_EYR40_007784/gene_EYR40_005038/gene_EYR40_		EYR40_004512/gene_EYR40_003499/gene_EYR40_009186/gene_EYR40_007431/gene_EYR40_005568/gene_EYR40_001771/gene_EYR40_009694/gene_EYR40_004757/gene_EYR40_010365/gene_EYR40_000254/gene_EYR40_009492/gene_EYR40_002854/gene_EYR40_007792/gene_EYR40_00

Category	GOID	Description	geneID	geneName	Count	Up	Up_Gene_id	Down	Down_Gene_id
			0/gene_EYR40_000254/gene_EYR40_009492/gene_EYR40_007944/gene_EYR40_003020/gene_EYR40_002854/gene_EYR40_007784/gene_EYR40_005038/gene_EYR40_007055/gene_EYR40_009912/gene_EYR40_010393/gene_EYR40_007546/gene_EYR40_007792/gene_EYR40_000091/gene_EYR40_005087/gene_EYR40_005086/gene_EYR40_008240/gene_EYR40_004232/gene_EYR40_007317/gene_EYR40_007618/gene_EYR40	490/EYR40_000254/EYR40_009492/EYR40_007944/EYR40_003020/FTR1/ERG28/EYR40_005038/EYR40_007055/EYR40_009912/EYR40_010393/EYR40_007546/EYR40_007792/EYR40_000091/EYR40_005087/EYR40_005086/EYR40_008240/EYR40_004232/EYR40_007317/EYR40_007618/EYR40_002			007055/gene_EYR40_009912/gene_EYR40_010393/gene_EYR40_007546/gene_EYR40_000091/gene_EYR40_008240/gene_EYR40_007317/gene_EYR40_007618		5087/gene_EYR40_005086/gene_EYR40_004232/gene_EYR40_002030

Category	GOID	Description	geneID	geneName	Count	Up	Up_Gene_id	Down	Down_Gene_id
			_002030	030					
CC	GO:0044425	membrane part	gene_EYR40_007606/gene_EYR40_006360/gene_EYR40_006760/gene_EYR40_002267/gene_EYR40_008579/gene_EYR40_006157/gene_EYR40_001574/gene_EYR40_009212/gene_EYR40_004512/gene_EYR40_001205/gene_EYR40_007351/gene_EYR40_009863/gene_EYR40_003499/gene_EYR40_003993/gene_EYR40_009186/gene_EYR40_006361/gene_EYR40_009862/gene_EYR40_007818/gene_EYR40_0074	EYR40_007606/EYR40_006360/EYR40_006760/HXT1_1/EYR40_008579/EYR40_006157/EYR40_001574/EYR40_009212/EYR40_004512/EYR40_001205/EYR40_007351/EYR40_009863/EYR40_003499/EYR40_003993/EYR40_009186/EYR40_006361/EYR40_009862/PHO84_2/EYR40_00743	46	25	gene_EYR40_006360/gene_EYR40_006157/gene_EYR40_001574/gene_EYR40_009212/gene_EYR40_001205/gene_EYR40_007351/gene_EYR40_009863/gene_EYR40_003993/gene_EYR40_006361/gene_EYR40_009862/gene_EYR40_007818/gene_EYR40_000179/gene_EYR40_002	21	gene_EYR40_007606/gene_EYR40_006760/gene_EYR40_002267/gene_EYR40_008579/gene_EYR40_004512/gene_EYR40_003499/gene_EYR40_009186/gene_EYR40_007431/gene_EYR40_005568/gene_EYR40_001771/gene_EYR40_009694/gene_EYR40_004757/gene_EYR40_01036

Category	GOID	Description	geneID	geneName	Count	Up	Up_Gene_id	Down	Down_Gene_id
			31/gene_EYR40_005568/gene_EYR40_001771/gene_EYR40_009694/gene_EYR40_004757/gene_EYR40_010365/gene_EYR40_000179/gene_EYR40_002490/gene_EYR40_000254/gene_EYR40_009492/gene_EYR40_007944/gene_EYR40_003020/gene_EYR40_002854/gene_EYR40_007784/gene_EYR40_005038/gene_EYR40_007055/gene_EYR40_009912/gene_EYR40_010393/gene_EYR40_007546/gene_EYR40_007792/gene_EYR	1/EYR40_005568/EYR40_001771/EYR40_009694/EYR40_004757/EYR40_010365/EYR40_000179/EYR40_002490/EYR40_000254/EYR40_009492/EYR40_007944/EYR40_003020/FTR1/ERG28/EYR40_005038/EYR40_007055/EYR40_009912/EYR40_010393/EYR40_007546/EYR40_007792/EYR40_000091/			490/gene_EYR40_007944/gene_EYR40_003020/gene_EYR40_007784/gene_EYR40_005038/gene_EYR40_007055/gene_EYR40_009912/gene_EYR40_010393/gene_EYR40_007546/gene_EYR40_000091/gene_EYR40_008240/gene_EYR40_007317/gene_EYR40_007618		5/gene_EYR40_000254/gene_EYR40_009492/gene_EYR40_002854/gene_EYR40_007792/gene_EYR40_005087/gene_EYR40_005086/gene_EYR40_004232/gene_EYR40_002030

Category	GOID	Description	geneID	geneName	Count	Up	Up_Gene_id	Down	Down_Gene_id
			40_000091/gene_EYR40_005087/gene_EYR40_005086/gene_EYR40_008240/gene_EYR40_004232/gene_EYR40_007317/gene_EYR40_007618/gene_EYR40_002030	EYR40_005087/EYR40_005086/EYR40_008240/EYR40_004232/EYR40_007317/EYR40_007618/EYR40_002030					

Category： GO database classification, including Biological Process BP, Cellular Component CC, Molecular Function MF,GOID： GO number,Description： Functional description corresponding to GO number,geneID： Differential gene ID annotated to GO number,geneName： Differential gene name annotated to GO number,Count： Number of differential genes annotated to GO number,COUNT： Number of differential genes annotated to GO number. COUNT: the number of differential genes annotated to the GO number Up: number of up-regulated differential genes associated with the Term,Up_Gene_id: ID of up-regulated differential genes associated with the Term,Down: number of down-regulated differential genes associated with the Term,Down_Gene_id: ID of down-regulated differential genes associated with the Term.

Group	fresh weight (g)	dry weight (g)	moisture content (%)	stem diameter (mm)	stem length (mm)	cap thickness (mm)	cap length (mm)	cap width (mm)
CK	124.74±22.35ab	14.42±1.66a	14.42±1.66a	12.05±0.25a	52.39±5.77a	3.44±0.28a	28.65±1.20ab	27.95±1.79a
A5	82.54 ±4.68bc	9.18±0.10b	13.05±2.00a	10.83±1.02ab	38.12±1.25a	4.97±0.81a	23.80±0.22b	20.89±0.20b
A10	147.04±19.62a	14.31±0.76a	12.13±0.71a	10.91±0.88ab	49.75±5.95a	4.39±0.33a	30.90±2.39a	29.42±2.50a
A20	45.15±12.74c	5.15±0.98c	10.77±0.95a	9.09±0.91b	38.36±3.97a	4.04±0.29a	27.85±1.67ab	26.09±1.95ab
CK	124.74±22.35a	14.42±1.66a	0.88±0.01a	12.05±0.25a	52.39±5.77a	3.44±0.28b	28.65±1.20a	27.95±1.79a
B5	126.72±12.87a	13.05±2.00a	0.90±0.01a	10.25±0.74a	47.94±2.00a	4.90±0.30a	30.15±1.36a	28.63±0.93a
B10	122.27±9.13a	12.13±0.71a	0.90±0.00a	12.12±0.27a	48.72±2.73a	5.02±0.18a	31.62±2.12a	30.86±1.91a
B20	110.17±6.78a	10.77±0.95a	0.90±0.01a	10.52±1.31a	45.55±2.01a	3.86±0.42b	28.13±0.32a	28.13±1.14a

Table S4. Summary Table of Agronomic Parameters
